# Constrained catecholamines gain β_2_AR selectivity through allosteric effects on pocket dynamics

**DOI:** 10.1038/s41467-023-37808-y

**Published:** 2023-04-14

**Authors:** Xinyu Xu, Jeremy Shonberg, Jonas Kaindl, Mary J. Clark, Anne Stößel, Luis Maul, Daniel Mayer, Harald Hübner, Kunio Hirata, A. J. Venkatakrishnan, Ron O. Dror, Brian K. Kobilka, Roger K. Sunahara, Xiangyu Liu, Peter Gmeiner

**Affiliations:** 1 State Key laboratory of Membrane Biology, Tsinghua-Peking Center for Life Sciences, School of Pharmaceutical Sciences, Tsinghua University, Beijing, 100084 China; 2 Beijing Frontier Research Center for Biological Structure, Beijing Advanced Innovation Center for Structural Biology, Tsinghua University, Beijing, 100084 China; 3 Department of Chemistry and Pharmacy, Medicinal Chemistry, Friedrich-Alexander University Erlangen-Nurnberg, Nikolaus-Fiebiger-Straße 10, 91058 Erlangen, Germany; 4 Department of Pharmacology, University of California San Diego School of Medicine, 9500 Gilman Drive, La Jolla, California 92093 USA; 5 Advanced Photon Technology Division, Research Infrastructure Group, SR Life Science Instrumentation Unit, RIKEN/SPring-8 Center, 1-1-1 Kouto Sayo-cho Sayo-gun, Hyogo, 679-5148 Japan; 6 Precursory Research for Embryonic Science and Technology (PRESTO), Japan Science and Technology Agency, 4-1-8 Honcho, Kawaguchi, Saitama 332-0012 Japan; 7 Department of Computer Science, Stanford University, Stanford, CA 94305 USA; 8 Department of Molecular and Cellular Physiology, Stanford University School of Medicine, Stanford, CA 94305 USA; 9 Department of Structural Biology, Stanford University School of Medicine, Stanford, CA 94305 USA; 10 Institute for Computational and Mathematical Engineering, Stanford University, Stanford, CA 94305 USA; 11 Beijing Key Laboratory of Cardiovascular Receptors Research, Peking University, Beijing, China

**Keywords:** X-ray crystallography, Small molecules, G protein-coupled receptors

## Abstract

G protein-coupled receptors (GPCRs) within the same subfamily often share high homology in their orthosteric pocket and therefore pose challenges to drug development. The amino acids that form the orthosteric binding pocket for epinephrine and norepinephrine in the β_1_ and β_2_ adrenergic receptors (β_1_AR and β_2_AR) are identical. Here, to examine the effect of conformational restriction on ligand binding kinetics, we synthesized a constrained form of epinephrine. Surprisingly, the constrained epinephrine exhibits over 100-fold selectivity for the β_2_AR over the β_1_AR. We provide evidence that the selectivity may be due to reduced ligand flexibility that enhances the association rate for the β_2_AR, as well as a less stable binding pocket for constrained epinephrine in the β_1_AR. The differences in the amino acid sequence of the extracellular vestibule of the β_1_AR allosterically alter the shape and stability of the binding pocket, resulting in a marked difference in affinity compared to the β_2_AR. These studies suggest that for receptors containing identical binding pocket residues, the binding selectivity may be influenced in an allosteric manner by surrounding residues, like those of the extracellular loops (ECLs) that form the vestibule. Exploiting these allosteric influences may facilitate the development of more subtype-selective ligands for GPCRs.

## Abstract (web summary)

Constrained catecholamines gain β_2_AR selectivity. Although the orthosteric pockets are identical in β_1_AR and β_2_AR, surrounding residues allosterically modify the pockets and contribute to the β_2_AR selectivity of the constrained catecholamines.

## Introduction

G protein-coupled receptors (GPCRs) are of great interest as therapeutic targets^1^. One of the major challenges in drug discovery efforts is to minimize off-target side effects, and to achieve greater subtype selectivity of candidate drugs^2^. Many pharmaceutically important GPCRs have multiple closely related subtypes that fulfill different physiologic roles, but are all activated by the same hormone or neurotransmitter. For example, the nine adrenergic receptor subtypes have different roles in regulating central and sympathetic nervous system functions, but can all be activated by epinephrine (Epi) and norepinephrine (NorEpi), as they share a highly homologous orthosteric binding pocket.

The β_1_ and β_2_ adrenergic receptors (ARs) have been among the most extensively studied G protein-coupled receptors (GPCRs) due to their roles in the regulation of cardiac and pulmonary function by the autonomic nervous system. βAR antagonists were among the first GPCR drugs to be developed and β_1_AR-selective antagonists are used for the treatment of heart failure, arrhythmias, and hypertension^3^. β_2_AR-selective agonists have been used in the treatment of asthma and chronic obstructive pulmonary disease^4^. β_1_AR and β_2_AR are equally responsive to Epi, primarily secreted by the adrenal gland, in contrast to NorEpi, which is primarily released from sympathetic nerve terminals, and is approximately 10-fold more selective for the β_1_AR^5^.

**Fig. 1:**
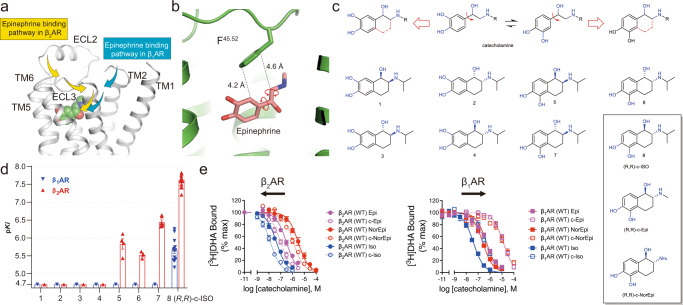
Design and synthesis of conformationally-constrained catecholamine. **a** Epi favors different binding pathways to enter the orthosteric pockets in the β_1_AR and β_2_AR^7^. **b** There is space between Epi and F^45.52^ to allow the receptor to accommodate for the conformational restriction of the catecholamine (PDB: 4LDO). **c** The design and synthesis flow of eight possible conformationally-constrained isoprenaline isomers and the chemical structures of (R,R)-c-Epi and (R,R)-c-NorEpi. **d** The (R,R)-isomer of c-ISO showed the highest affinity to both the β_1_AR and β_2_AR among all the eight possible isomers. Data were given as mean ± SEM of *n* = 3 (for **1**-**4**,**6**), *n* = 4 (for **5**), *n* = 5 (β_1_AR for **7**), *n* = 6 (β_2_AR for **7**), *n* = 13 (β_2_AR for **8**), and *n* = 14 (β_1_AR for **8**) independent experiments. **e** c-ISO, c-Epi, and c-NorEpi showed β_2_AR selectivity in a radioligand competition binding assay. Data were given as mean ± SEM of *n* = 3 (β_1_AR for Epi), *n* = 3(β_2_AR for Epi), *n* = 3 (β_1_AR for NorEpi), *n* = 5(β_2_AR for NorEpi), *n* = 3 (β_1_AR for ISO), *n* = 3(β_2_AR for ISO), *n* = 6 (β_1_AR for c-Epi), *n* = 6 (β_2_AR for c-Epi), *n* = 5 (β_1_AR for c-NorEpi), *n* = 5(β_2_AR for c-NorEpi), *n* = 3 (β_1_AR for cISO), *n* = 3(β_2_AR for cISO) independent experiments. Source data are provided as a Source Data file.

Previous molecular dynamics (MD) simulations of alprenolol and other ligands binding to the β_2_AR^6^ reveal that ligands typically access the orthosteric binding pocket through a portal formed by a narrow cleft, lined by residues in the extracellular vestibule. Recent studies suggest that the preferred pathway may differ for the β_1_AR and β_2_AR^7^ (Fig. 1a). MD simulations suggest that ligand flexibility might be important for navigating through the extracellular vestibule. Ligand flexibility, however, also yields an entropic penalty when the ligand arrives at the orthosteric pocket, where it is constrained to a single or limited set of conformations. The principle sites of flexibility in catecholamines are contributed by two rotatable bonds, one between the catechol ring and the ethanolamine moiety and one within the ethanolamine itself (see Fig. 1b). A key finding revealed in structural studies of Epi-, NorEpi- and isoproterenol (ISO)-bound β-ARs is the selection of one specific set of rotamers of the catecholamines^8,9^.

In this work, to examine the effect of ligand flexibility and the entropic contribution to catecholamine binding to β_1_AR and β_2_AR, we generated conformationally-constrained forms of Epi, NorEpi, and ISO to restrict or constrain the rotatable bonds and characterized their pharmacology and signaling behavior. We show that the constrained catecholamines gain β_2_AR selectivity over the β_1_AR despite the orthosteric ligand binding sites are identical in these two receptors. We further show that allosteric effects from surrounding residues of the orthosteric pockets alter the shape of the pockets and contribute to the β_2_AR selectivity of the constrained catecholamines.

## Results

### Design and characterization of constrained catecholamines

**Fig. 2:**
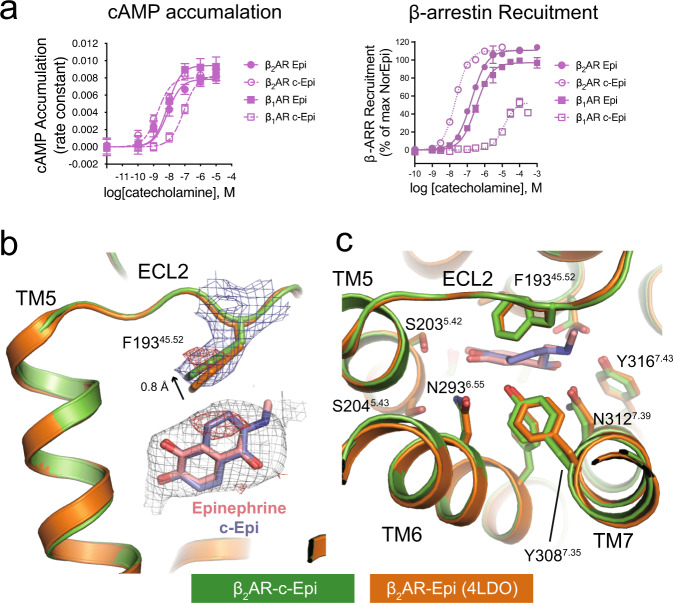
c-Epi is selective towards the β_2_AR and binds to the same position as Epi does. **a** c-Epi shows β_2_AR selectivity in both cAMP recruitment and arrestin signaling assays. Data were given as mean ± SEM of *n* = 6 (β_1_AR for Epi, arrestin), *n* = 15 (β_1_AR for c-Epi, arrestin), *n* = 17 (β_2_AR for Epi, arrestin), and *n* = 21 (β_2_AR for c-Epi, arrestin) independent experiments. Source data are provided as a Source data file. **b** c-Epi binds in a nearly identical position as Epi in the β_2_AR as revealed by the 2fofc density (blue mesh, contoured at 1.0 σ). The extra two carbons of c-Epi compared to Epi as well as the upward movement of F^45.52^ are revealed by the isomorphous difference map (red mesh, contoured at 3.0 σ). **c** The binding pocket residues are in similar positions upon Epi or c-Epi binding.

Two bridging carbons were introduced to constrain the conformation of the catecholamines. Because there are two possible ways to rigidify the compound and each constrained compound has two chiral carbons, each catecholamine will have eight possible constrained isomers (Fig. 1c). Compounds 1–4 would not be expected to fit into the previously reported Epi and NorEpi binding pose; nevertheless, we decided to synthesize and test them for completeness (Supplementary Notes). The synthesis of a subset of similar compounds has previously been described; however, these compounds were not enantiomerically pure, and their pharmacology was not fully characterized^10^. We synthesized all the eight possible isomers of ISO (Supplementary Notes). Of which, the *(R,R)* form (compound 8, c-ISO) showed the highest affinity for both the β_1_AR and the β_2_AR, but not the αARs, in radioligand competition binding assay (Fig. 1d and Supplementary Table 1). Interestingly, the *(R,R)*-isomer also showed selectivity towards the β_2_AR over β_1_AR (Fig. 1e and Supplementary Table 2). Consequently, we synthesized the constrained *(R,R)*-isomers of Epi and NorEpi (hereby named c-Epi or c-NorEpi) (Supplementary Notes) and characterized their pharmacological properties side-by-side with the non-constrained forms. Compared to Epi, which has a similar EC_50_ in G protein activation, adenylyl cyclase activation, and arrestin recruitment for both the β_1_AR and the β_2_AR, c-Epi exhibited both an increase in potency at the β_2_AR and a decrease at the β_1_AR. On the β_2_AR, c-Epi has a threefold lower EC_50_ for G protein activation and adenylyl cyclase stimulation and a 7.5-fold reduction in EC_50_ in an arrestin recruitment assay, compared to the non-rigidified native hormone (Fig. 2a, Supplementary Fig. 1, Supplementary Tables 2,3). In striking contrast, c-Epi at the β_1_AR yields a ~14-fold increase in the EC_50_ in cyclase stimulation and a ~19-fold increase in the EC_50_ in arrestin recruitment. Radioligand competition binding assays indicate that constrained Epi bound eightfold better on the β_2_AR but 34-fold worse on the β_1_AR, compared to unconstrained Epi (Fig. 1e and Supplementary Table 4). Thus, while Epi does not display a preference for either β_1_AR or β_2_AR in an arrestin recruitment or radioligand binding assays, c-Epi recruits β-arrestin with an ~600-fold selectivity for β_2_AR over β_1_AR. We also note a substantial reduction (~40%) in the E_max_ for c-Epi-induced arrestin recruitment to β_1_AR (Fig. 2a and Supplementary Table 3).

Perhaps more interesting is the complete switch in β_1_AR-β_2_AR selectivity of NorEpi upon constraining the catecholamine. In β-arrestin recruitment assays NorEpi has ~16-fold selectivity for β_1_AR over β_2_AR, whereas c-NorEpi displays a 52-fold selectivity for β_2_AR over β_1_AR (Supplementary Fig. 2 and Supplementary Table 3). For isoproterenol, where the EC_50_ for β_1_AR and β_2_AR are nearly identical (19 and 16 nM, respectively) and thus, no subtype selectivity. Constrained ISO, however, displays greater than a 70-fold selectivity for β_2_AR afforded by a greater than a 100-fold decrease in the potency at β_1_AR for recruiting β-arrestin (Supplementary Fig. 2 and Supplementary Table 3). Radioligand binding analysis suggests that this dramatic increase in β_2_AR selectivity was contributed by a tenfold decrease in affinity for β_1_AR, with a ~3-fold improvement in β_2_AR affinity. (Fig. 1e and Supplementary Table 4).

An additional side note is that neither c-NorEpi, c-Epi or c-ISO displayed any appreciable activity for the α_1_AR or α_2_AR subtypes, suggesting that constraining the catecholamines garners the agonists β_2_AR-selective over all adrenergic receptors (Supplementary Fig. 3 and Supplementary Table 2).

### Structure determination of the β_2_AR–c-Epi complex

What is most striking about the enhanced potency of these constrained catecholamines on β_2_AR is the marked decrease in potency at the β_1_AR. In an attempt to understand the structural basis for this subtype selectivity, we solved the crystal structures of the β_2_AR in complex with c-Epi and c-ISO at 3.2 and 3.4 Å resolution, respectively. The structures were obtained with a previously described nanobody Nb6B9 and a β_2_AR construct with T4 lysozyme fused to the N-terminus (T4L-β_2_AR)^8^. Nb6B9 binds to the intracellular surface of the β_2_AR, the G protein binding site, and stabilizes its active conformation.

The c-Epi binding site is clearly revealed by a simulated omit map suggesting that c-Epi and Epi bind in nearly identical positions (Fig. 2b). The bridging carbons that constrain Epi in c-Epi are clearly revealed by an isomorphous difference map between the β_2_AR–c-Epi data and the β_2_AR-Epi data (Fig. 2b, red mesh). When comparing the β_2_AR-Epi structure and β_2_AR-c-Epi structure, all the orthosteric pocket residues are in similar positions (Fig. 2c). The F193^45.52^ side chain is displaced slightly upward (~0.8 Å) in the c-Epi bound structure compared to the Epi bound structure, supported by the 2fofc map and isomorphous difference map between the β_2_AR-c-Epi and β_2_AR-Epi data (Fig. 2b). Similar upward movement of F193^45.52^ is observed in the β_2_AR-c-ISO structure (Supplementary Fig. 4). Even though the β_2_AR-c-Epi and β_2_AR-Epi structures are remarkably similar (RMSD of 0.3 Å for all Cα atoms), structure analysis suggests that c-Epi binding provides additional stabilization of ECL3 and the C-terminal end of ECL2, as revealed by a reduction in normalized b-factors (Supplementary Fig. 5).

As previously mentioned, all residues that form the orthosteric pocket (as defined within 4 Å of the ligand) of Epi are conserved between the β_2_AR and the β_1_AR. One residue that appears within 3.5–4.5 Å away from bridging carbons of c-Epi is Y308^7.35^. This residue is phenylalanine in the β_1_AR (F359^7.35^), suggesting that the Y308^7.35^’s hydroxyl moiety could account for the affinity difference between β_2_AR and the β_1_AR. Substituting Y308^7.35^ to phenylalanine in β_2_AR, however, exhibited only a modest decrease in c-Epi affinity (Supplementary Fig. 6). Likewise, the β_1_AR-F359^7.35^Y mutant displayed an almost identical affinity for c-Epi compared to the wild-type β_1_AR. Taken together, these data suggest that the amino acid difference of Y/F^7.35^ is not the key determinant of selectivity.

**Fig. 3:**
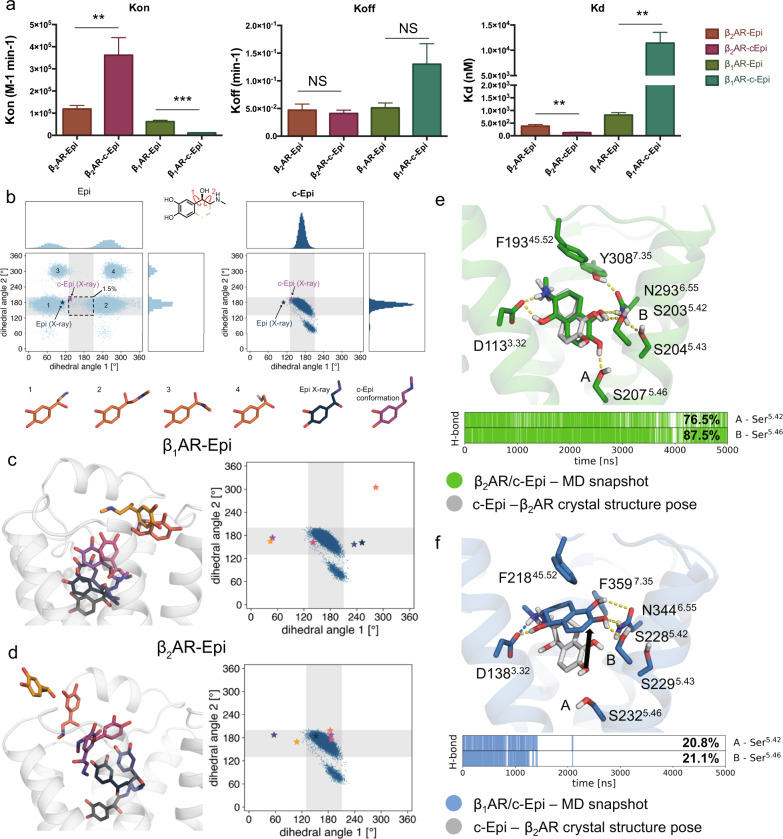
The selectivity of c-Epi toward the β_2_AR reflects differences in the binding pathways and less stable orthosteric binding pocket interactions in the β_1_AR. **a** Binding kinetics studies of Epi and c-Epi towards the β_1_AR and β_2_AR. Statistic analysis were performed using two-way ANOVA analysis. (*P < 0.05, **P < 0.005, ***P < 0.0005). Data were given as mean ± SEM of *n* = 5 (β_2_AR for Epi), *n* = 5 (β_2_AR for c-Epi), *n* = 3 (β_1_AR for Epi), *n* = 3 (β_1_AR for c-Epi), and independent experiments. Source data are provided as a Source data file. **b** MD simulations suggest that Epi adopts a wide spectrum of conformations in the solution that rarely matches its bioactive conformation, while the conformational restriction limits the catecholamine in c-Epi to the bioactive conformation. The values of two dihedral angles of the aliphatic part of Epi adopted in simulations are plotted against each other. The stars indicate the values for Epi and c-Epi as present in the β_2_AR crystal structures. The dashed rectangle is a rough measure for the conformational space occupied by c-Epi in solution. The percentage value indicates the frequency by which Epi entered this space. **c**, **d** Epi adopts different conformations during the binding process to the β_1_AR (**c**) and β_2_AR (**d**)^33^. The conformational restriction of c-Epi is incompatible with this proposed path for β_1_AR, but is less so for β_2_AR. The different poses of Epi represent a potential path of binding suggested by metadynamics simulations. The plot displays the dihedral angles adopted by c-Epi in solution as described for **b**. The stars show the dihedral adopted by Epi in the stages of association shown in the same color on the respective left side. **e** c-Epi maintains its crystallographic pose in the β_2_AR in simulations. A representative frame of the simulations is displayed in green and the crystallographic pose of c-Epi in gray. **f** In β_1_AR-c-Epi does not maintain the expected bioactive conformation in simulations. A representative frame of the simulations is displayed in blue and the crystallographic pose of c-Epi in the β_2_AR in gray. **e**, **f** The trace plot, indicated the presence of the canonical hydrogen bonds to Ser^5.42^ and Ser^5.46^. The trace is displayed for one representative trajectory. The percentage value is the mean over six simulations, 5 µs each.

The differences in affinity observed for constrained and non-constrained catecholamines could be due to differences in agonist on-rates, off-rates, or both. We, therefore, analyzed the kinetics of c-Epi and c-NorEpi binding to both β_1_AR and β_2_AR. c-Epi exhibits a ~3-fold faster association rate with the β_2_AR than Epi, suggesting that conformational restriction facilitates ligand binding, probably due to entropic advantages and perhaps influenced by the increased hydrophobicity offered by the addition of the cyclic ring (Fig. 3a). Indeed, MD simulations suggest that Epi adopts a wide spectrum of conformations in the solution that rarely match its bioactive conformation, the conformation observed in the active state structure of the β_2_AR bound to Epi (Fig. 2b). In contrast, c-Epi appears to exist almost exclusively in the bioactive conformation (Fig. 3b). The entropic gain that results from the conformational restriction could account for the faster association rate of c-Epi compared to Epi with the β_2_AR. c-NorEpi also displays a modest ~2-fold faster association rate than NorEpi to the β_2_AR, suggesting a common mechanism for differences in the association rate (Supplementary Fig. 7). The dissociation rates of both c-Epi and c-NorEpi from β_2_AR were not statistically different compared to Epi or NorEpi, respectively (Fig. 3a and Supplementary Fig. 7).

In contrast, the association rates at β_1_AR were significantly slower for c-Epi (~5-fold) and even slower for c-NorEpi (~15-fold) compared to the non-constrained catecholamines. The slower on-rate of c-Epi and c-NorEpi suggests that the portal to the orthosteric site on β_1_AR may be less compatible with the constrained catecholamines. The faster on-rate kinetics on β_2_AR are consistent with metadynamics simulations sampling of possible ligand binding pathways. While Epi adopts various compatible conformations during the process of binding to β_1_AR, c-Epi does not (Fig. 3c). This is in contrast to β_2_AR, where c-Epi’s limited conformations appear more compatible during the process of binding to the orthosteric site (Fig. 3d). This may contribute to the slower on-rate of c-Epi compared to Epi for the β_1_AR.

c-Epi or c-NorEpi also have a 2–3-fold increased dissociation rate for the β_1_AR compared to Epi or NorEpi (Fig. 3a and Supplementary Fig. 7), although this difference did not reach statistical significance, it is consistent with MD simulations of c-Epi binding to the orthosteric site of β_1_AR. Hydrogen bonding of the meta and para-hydroxyl on the catechol ring of catecholamines to two Ser residues on TM5 are critical for binding and efficacy on all catecholamine receptors. MD simulations show that c-Epi is much less stable in the binding pocket of the β_1_AR, as revealed by a large conformational shift of the ligand in simulations and a loss of H-bonding with TM5 (Fig. 3e, f). The canonical H bond between meta-hydroxyl of c-Epi and Ser^5.42^, as well as the H bond between para-hydroxyl of c-Epi and Ser^5.46^ occur 76.5 and 87.5% of the time between c-Epi and the β_2_AR, while the same H bonds only occur 20.8 and 21.1% of the time between c-Epi and the β_1_AR (Fig. 3e, f). Thus, the lower affinity of c-Epi or c-NorEpi for the β_1_AR compared to the β_2_AR is thus likely due to a combined effect of ligand entropy, less compatible conformations during the binding process, and reduced stability of the ligand binding pocket.

It is important to note that the binding site modeled from crystallographic data reflects the binding site of the active conformation, which is stabilized by the G protein-mimicking nanobody. Differences in access to the orthosteric site may likely change in the active conformation compared to the inactive state. Data from pharmacological studies suggest that access to (association rate) or escape from (dissociation rate) the β_2_AR orthosteric site differs significantly in the inactive and active states^11^.

### The role of the vestibule formed by the ECLs

The MD simulations on alprenolol binding to the β_2_AR show transient interactions within the vestibule before ligand entry in the orthosteric site^6^. This was also observed in simulations following ligand binding to the muscarinic M_3_ and M_4_ acetylcholine receptor^12^. The vestibule on β_2_AR, located directly above the orthosteric site, is composed of residues contributed by the ECLs. We previously demonstrated that replacing the ECLs of β_2_AR with that of β_1_AR could confer a complete switch to high-affinity NorEpi binding by accelerating the association rate^7^. With the exception of Y308^7.35^ (F359^6.58^ in the β_1_AR), the residues within orthosteric site of β_2_AR and β_1_AR that coordinate Epi binding are identical. As noted above we observed very little differences in Y^6.58^ or F^6.58^ in c-Epi binding. The conserved aromatic residue F^45.52^ which contributes toward the formation of a lid over the orthosteric pocket drew our attention. We and others have shown that residue 45.52 affects the association and dissociation rates of ligands and arrestin signaling in other class A GPCRs^13,14^. Our structures reveal that F193^45.52^ on β_2_AR needs to move up by ~0.8 Å in order to accommodate the two carbons that rigidifies c-Epi. We previously showed that F^45.52^, with contributions from different surrounding residues, has significant effects on NorEpi or Epi affinity^7^. The F^45.52^A mutation has a larger effect in reducing Epi and NorEpi’s affinity for the β_1_AR than β_2_AR (~250-fold increase in Ki values for Epi and NorEpi for the β_1_AR, but only a 50-fold increase for Epi and 3-fold for NorEpi for the β_2_AR)^7^. In contrast, the same mutation has a larger effect in reducing c-Epi affinity for the β_2_AR than β_1_AR (Supplementary Fig. 8).

Analysis of the pharmacological properties of the constrained catecholamines using β_1_AR-β_2_AR chimeras, where the extracellular vestibules (consisting of the ECLs as well as the extracellular ends of TMs) of β_1_AR and β_2_AR were exchanged (Supplementary Fig. 9)^7^, strongly suggests that residues within the vestibule surrounding the orthosteric pocket confer the majority of subtype selectivity of the c-Epi and c-NorEpi. As summarized in Supplemental Table 5 and Supplementary Fig. 10, replacing the extracellular vestibule of the β_2_AR with that of β_1_AR (β_2_AR_in_/β_1_AR_out_) eliminates the enhanced on-rate and accelerates the off-rates of the constrained catecholamines yielding rate constants comparable to wild-type β_1_AR. Likewise, replacing the extracellular vestibule of β_1_AR with that of β_2_AR (β_1_AR_in_/β_2_AR_out_) results in a minimal change in on-rates and reduced off-rates yielding rate constants comparable to wild-type β_2_AR. In agreement with the binding kinetics data, replacing the extracellular vestibule also results in the change of EC_50_ for all three constrained catecholamines in the arrestin recruitment assay (Supplementary Fig. 11).

**Fig. 4:**
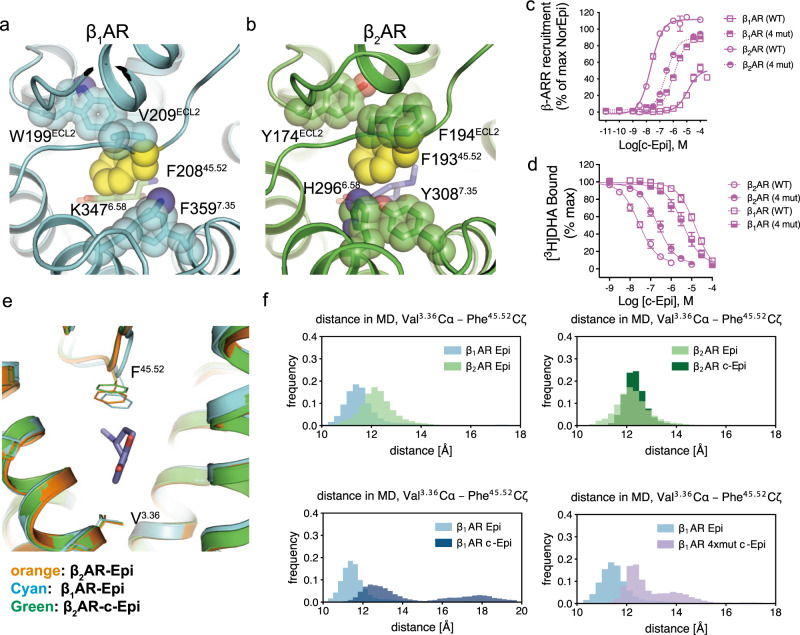
Mutating four residues around the F^45.52^ affect c-Epi affinities in the β_1_AR and β_2_AR. **a**, **b** F^45.52^ (yellow spheres) is conserved in the β_1_AR and β_2_AR, but is surrounded by differing residues. The different surrounding residues are W199^ECL2^, V209^ECL2^, K347^6.58^, and F359^7.35^ in the β_1_AR (**a**) and Y174^ECL2^, F194^ECL2^, H296^6.58^, and Y308^7.35^ in the β_2_AR (**b**). **c**, **d** Mutating the four residues surrounding F^45.52^ reduced c-Epi affinity to the β_2_AR and increased c-Epi affinity to the β_1_AR, in a β-arrestin recruitment assay (**c**) and in a competition binding assay (**d**). Data were given as mean ± SEM of *n* = 7 (β_1_AR (4 mut), β_2_AR (4 mut), arrestin), *n* = 15 (β_1_AR, arrestin), and *n* = 21 (β_2_AR, arrestin), *n* = 6 (β_1_AR, β_2_AR, β_1_AR (4 mut), β_2_AR (4 mut),DHA binding) independent experiments. Source data are provided as a Source data file. **e** F^45.52^ has a slightly different conformation in the β_2_AR-c-Epi, β_2_AR-Epi, and β_1_AR-Epi structures, and as a result, the distance between V^3.36^ and F^45.52^ is longer in the β_2_AR-c-Epi structure than in the β_2_AR-Epi structure, while the distance is the shortest in the β_1_AR-Epi structure. **f** The distribution for the distance between the Cα atom of V^3.36^ and the Cζ atom of F^45.52^ in MD simulations are displayed for the different simulation conditions. The distribution is shown in light blue for β_1_AR-Epi, dark blue for β_1_AR-c-Epi, light green for β_2_AR-Epi, dark green for β_2_AR-c-Epi and purple for β_1_AR_4mut-c-Epi. Cζ of F^45.52^ is located deeper in the pocket for β_1_AR-Epi compared to β_2_AR-Epi, and β_1_AR-c-Epi shows a similar distribution as β_1_AR-Epi. The deeper position in β_1_AR cannot accommodate for c-Epi and leads to a distortion of the F^45.52^ conformation and, therefore, to higher distances. For β_1_AR_4mut-c-Epi, distances at a β_2_AR-like level are observed, suggesting that the β_2_AR-like surrounding of F^45.52^ leads to conformations of F^45.52^ that are located less deep in the pocket as for β_1_AR-Epi.

In an attempt to pinpoint key residues within the extracellular vestibule that may contribute to the subtype selectivity offered by the constrained catecholamines, we performed mutagenesis studies to determine the role of key residues that surround F^45.52^, but are not directly involved in agonist binding. Out of the residues within a 5 Å distance to F^45.52^, four are different between the β_1_AR and β_2_AR. These are W199^ECL2^, V209^ECL2^, K347^6.58^, and F359^7.35^ in the β_1_AR (Fig. 4a), and Y174^ECL2^, F194^ECL2^, H296^6.58^, and Y308^7.35^ in the β_2_AR (Fig. 4b). Single point substitutions of each of the four residues on β_2_AR with those of β_1_AR decreased c-Epi affinity, with F194^ECL2^V having the largest effect. The reverse mutation in β_1_AR, V219^ECL2^F, is the only substitution out of the four that displays slightly increased c-Epi affinity. (Supplementary Fig. 12). Of note, previous studies showed that this pair of mutations (β_1_AR-V219^ECL2^F and β_2_AR-F194^ECL2^V) had little effect on NorEpi affinity^7^, suggesting that the selectivity mechanism of c-Epi for the β_2_AR is different from that of NorEpi for the β_1_AR.

While the individual single substitutions of the β_1_AR (W199^ECL2^Y, K347^6.58^H, and F359^7.35^Y) do not increase the receptor’s affinity for c-Epi, a combination of all these three mutations together with the V219^ECL2^F (β_1_AR-4mut) showed an increased c-Epi affinity qualitatively similar to the β_1_AR_in_/β_2_AR_out_ chimera (Fig. 4c, d, Supplementary Fig. 10, and Table 2). The results suggest that cooperation between different residues within the vestibule is required in order to establish a high-affinity c-Epi binding pocket in the β_1_AR. Combining all four mutations in the β_2_AR (β_2_AR-4mut) shows decreased c-Epi affinity compared to wild-type β_2_AR and is quantitively similar to the β_2_AR_in_/β_1_AR_out_ chimera (Supplementary Fig. 11 and Table 2). Interestingly, the reduced affinity of c-Epi on β_2_AR-4mut was not as large as the single substitution β_2_AR-F194^ECL2^V, highlighting the complex behavior of the allosteric interactions between residues. We further examined the effects of the four-residue substitutions on c-NorEpi and c-ISO. In arrestin recruitment assays, the β_2_AR-4mut displays decreased c-NorEpi and c-ISO potency compared to the β_2_AR, while β_1_AR-4mut shows increased c-NorEpi and c-ISO potency compared to the β_1_AR (Supplementary Fig. 13 and Supplementary Table 2).

To investigate the effect of the four-residue mutations on F^45.52^ conformation and dynamics, we simulated the binding of c-Epi to β_1_AR-4mut and compared the results with the MD simulations of Epi or c-Epi binding to wild-type β_1_AR and β_2_AR. As previously mentioned, c-Epi is not stable in the orthosteric pocket of β_1_AR and the canonical H bonds between the catecholamine and TM5 are seldom observed in the simulations. With β_1_AR-4mut, however, the c-Epi binding pose is more stable where canonical H bonds occur 43.9% of the time for the meta-hydroxyl and Ser^5.42^ and 55.4% of the time for para-hydroxyl and Ser^5.46^ (Supplementary Fig. 14a), all in good agreement with the functional data.

As observed in the crystal structure, the upward 0.8 Å movement of the F193^45.52^ side chain is required in order to accommodate the extra two carbon atoms in c-Epi. In the β_2_AR-Epi structure, the distance between the Cα atom of V117^3.36^, which is located at the bottom of the orthosteric site, and the Cζ atom of F193^45.52^ is 11 Å. In contrast, this V^3.36^/Cα – F^45.52^/Cζ distance is 11.7 Å in the β_2_AR-c-Epi structure and 10.6 Å in the β_1_AR-Epi structure. Similarly, MD simulations suggest that the V^3.36^/Cα – F^45.52^/Cζ distance is slightly longer in the β_2_AR-c-Epi complex compared to the β_2_AR-Epi complex, and slightly shorter in the β_1_AR-Epi complex compared to the β_2_AR-Epi complex (Fig. 4e, f). This small difference does not appear to have a major effect on the affinity of the smaller Epi, whereas the addition of the two carbons in c-Epi, requires the additional space created by the upward movement of F193^45.52^. It is likely that the allosteric effects of the residues surrounding F^45.52^ in β_1_AR do not permit its upward movement to the same extent as in β_2_AR without a rotamer change. MD simulations suggest that the side chain of F^45.52^ in the β_1_AR needs to flip away in order to avoid a clash with the c-Epi. This would require more flexibility of F^45.52^ and the surrounding region of the ECL2 in the β_1_AR-c-Epi simulation compared to the β_2_AR-c-Epi. Substituting the four surrounding residues, as with β_1_AR-4mut, results in reduced flexibility of F^45.52^ and ECL2 (Supplementary Fig. 14b), displaying a similar V^3.36^/Cα – F^45.52^/Cζ distance distribution as observed in the β_2_AR-c-Epi structure (Fig. 4e, f).

Of note, the F^45.52^ that is crucial for high-affinity binding of the β_2_AR to c-Epi is not conserved as Phe, but as branched-chain amino acids (leucine, isoleucine, valine) in the α_1_AR or α_2_AR subtypes (Supplementary Fig. 15a). Comparison with the α_2B_AR structure (PDB code: 6K41)^15^ suggests the sidechain of leucine may clash with c-Epi (Supplementary Fig. 15b). Binding studies also suggest F^45.52^V mutation decreases c-Epi affinity by ~100-fold in the β_2_AR (Supplementary Fig. 8b). The difference in position 45.52 may explain the lack of activity of constrained catecholamine to the αAR subtypes.

### Signaling and signal bias

In agreement with MD simulations and radioligand binding assays, the constrained catecholamines display enhanced potency on β_2_AR in adenylyl cyclase assays while decreasing their potency through β_1_AR stimulation (Fig. 2a). Similar enhanced effects of c-Epi and c-NorEpi were observed in β-arrestin recruitment, ie. enhanced potency on β_2_AR and diminished potency at β_1_AR, but with their magnitude of differences appear to be magnified in comparison to their responses in cyclase assays. Moreover, the diminished potency of the constrained catecholamines in β_1_AR-promoted β-arrestin recruitment assays (19- and 25-fold decreased potency of c-Epi and c-NorEpi, respectively) is accompanied by a decreased efficacy (~50% decreased efficacy of both c-Epi and c-NorEpi). It is likely that the β_1_AR, known to be a relatively poor arrestin-2 recruiter^16^ for catecholamines, exhibits an even poorer response to constrained catecholamines due to a combination of suboptimal binding at the hormone-binding site, as described above, and the relative instability of the vestibule and ECLs associated with F^45.52^. Thus, c-Epi and c-NorEpi confer a combined effect of lowering activity at β_1_AR while improving potency at β_2_AR, yielding an overall improvement in β_2_AR-β_1_AR selectivity.

To compare the relative effectiveness of the constrained catecholamines in cAMP and β-arrestin assays, we analyzed the dose-response relationships using an Operational Model of Bias, proposed by ref. ^17^. In brief, we first calculated the “transduction coefficient” of each ligand in cAMP and arrestin recruitment assays. The transduction coefficient is defined as log(τ/Ka), where τ represents the efficacy of the agonist and Ka represents the dissociation constant of the ligand (Supplementary Table 6a). Then the relative effectiveness (RE) between the two agonists were calculated as the inverse logarithm of Δlog(τ/Ka) between these two ligands. An RE >1 implies that a new agonist, relative to a control agonist, displays superior pharmacological properties based on efficacy and affinity. The bias factors, or the comparison between the RE of the two agonists in two separate signaling assays, was calculated as the inverse logarithm of ΔΔlog(τ/Ka) of the same pair of agonists in two different signaling pathways (Supplementary Table 6b). In cAMP assays with β_2_AR, c-Epi (RE~4), and c-NorEpi (RE ~20), compared to Epi and NorEpi, respectively, were both much higher than on β_1_AR (Supplemental Table 6). In β-arrestin recruitment assays, both c-Epi and c-NorEpi appeared dramatically more effective (c-Epi: RE~21, c-NorEpi: RE ~65) compared to Epi and NorEpi, respectively. Taken together, these data suggest that both c-Epi (bias factor ~5.4) and c-NorEpi (bias factor ~3.2) on β_2_AR display a signaling bias toward β-arrestin recruitment over adenylyl cyclase activity. It is important to note that although c-NorEpi displays a larger bias factor of ~ 51 on β_1_AR, the relative effectiveness (RE) for c-NorEpi on β_1_AR was ~2400-fold lower than NorEpi in cyclase assays and ~47-fold lower in β-arrestin recruitment assays. Interestingly, constraining isoproterenol had a major impact on β-arrestin recruitment through β_1_AR (100-fold decrease in potency compared to ISO) with little enhancing effect on β-arrestin recruitment by β_2_AR. This was accompanied by only modest effects on β_1_AR potency in cAMP assays and subtle effects on β_2_AR-mediated efficacy. Taken together, these data suggest that constraining isoproterenol appears as a β_1_AR-selective diminution, yielding a large effect on the relative effectiveness and hence biasing the signaling away from β-arrestin recruitment.

## Discussion

A common goal in drug development is to target specific receptor subtypes and avoid off-target binding events that often lead to adverse side effects. However, distinguishing specific receptor subtypes that natively bind the same hormone has been a major challenge in drug discovery. Indeed, advances in the structural biology of GPCRs have provided some insight into strategies to identify subtype-selective compounds. We previously reported the development of selective antagonists for the M_3_ muscarinic acetylcholine receptor and an orexin 1 receptor-selective antagonist based on single amino acid differences between these subtypes (M_3_AChR and M_2_AChR or OX_1_R and OX_2_R, respectively)^18,19^. In this study, we further show that even for GPCR subtypes like β_1_AR and β_2_AR that share identical orthosteric pockets, the shape and stability of the orthosteric pocket can be influenced by surrounding residues resulting in marked differences in ligand affinity.

Previous studies by ref. ^20^ revealed that entropy plays an important role in agonist binding to the turkey β_1_AR. The authors found that isoproterenol binding to turkey red blood cell membranes was highly dependent on temperature, with higher affinity observed at lower temperatures. Antagonist binding was unaffected by temperature. The authors concluded that agonist binding is associated with an unfavorable decrease in entropy. In this current study, we designed constrained catecholamines to determine the effect of reducing the entropic penalty of binding. Of the eight possible constrained enantiomers of iso (Fig. 1c), the *(R,R)*-enantiomer bound to the β_2_AR with the highest affinity. The constrained catecholamines c-NorEpi and c-Epi exhibited a faster association rate for binding to the β_2_AR compared to the non-constrained agonists (Fig. 3a and Supplementary Fig. 7), consistent with a reduced entropic penalty upon binding. However, we were surprised to find that conformationally-constrained catecholamine exhibited a high degree of β_2_AR selectivity, with a marked decrease in affinity for the β_1_AR as well as α_1_ARs and α_2_ARs (Supplementary Fig. 3). This selectivity for β_2_AR over β_1_AR was not due to differences in the residues that directly interact with the ligand in the orthosteric binding site. Binding kinetics studies and MD simulations suggest that the difference in affinity is due to a combined effect of the loss of flexibility needed to access the orthosteric pocket of the β_1_AR, and a less stable binding pose of c-Epi in the β_1_AR. When comparing the β_1_AR and β_2_AR, we found that the position of F^45.52^, common to both receptors, is slightly displaced in the β_1_AR relative to the β_2_AR. This difference in the orientation of F^45.52^ (a smaller V^3.36^/Cα – F^45.52^/Cζ distance), which leads to a change in the shape of the binding pocket and accounting for the apparent subtype selectivity, can largely be attributed to as few as four aromatic amino acids in β_2_AR ECLs that surround F^45.52^.

Entropic gains with the constrained catecholamines enhanced β_2_AR affinity with a simultaneous decrease in binding affinity for β_1_AR, observable in ligand binding and G protein coupling assays. What was unanticipated was the greater magnitude of differences in responses in β-arrestin recruitment where the constrained catecholamines appear to have a diminutive effect on β_1_AR, while enhancing the potency at β_2_AR, in the case of c-Epi and c-NorEpi. Thus, constraining NorEpi completely switched the β_1_AR-β_2_AR selectivity from β_1_AR-selective NorEpi responses to β_2_AR-selective arrestin recruitment with c-NorEpi. The mechanism for the magnitude differences in β-arrestin recruitment compared to G protein or cyclase assays, ie. enhancement for β_2_AR and diminution with β_1_AR, is unclear but potentially related to one clear structural difference in the catecholamine-bound β-ARs versus the constrained counterparts. Constraining the catecholamines through the addition of two carbon atoms and the formation of a second cyclic ring, impacts F^45.52^ in both β_1_AR and β_2_AR, altering the structures of the ECLs and vestibule located above the orthosteric, hormone-binding site. Accordingly, c-Epi binding to β_1_AR would require significant changes in the conformation of F^45.52^ and ECL2, whereas c-Epi binding to β_2_AR appears to stabilize F^45.52^ and ECL2 in a conformation that favors arrestin recruitment. Indeed, previous studies on the serotonin 5-HT_2B_ receptor suggest that ligands that influence the structure of ECLs appear to influence G protein and arrestin bias^13,14^. Structural data on β_2_AR-arrestin complexes stabilized with c-Epi or c-NorEpi might provide some insight into how the ECLs may be involved in arrestin recruitment and hence help to delineate the basis for the observed effect on signaling bias.

Orthosteric pockets being allosterically modified by surrounding residues, such as those in the ECLs, are unlikely to be a unique case for adrenergic receptors. This study suggests that it may be possible to develop subtype-selective drugs for other GPCRs by carefully exploring the dynamics of the orthosteric pocket differences between subtypes. In this regard, high-resolution structures and MD simulations provide valuable information to guide drug development.

## Methods

### Protein expression and purification

A previously reported human β_2_AR – T4 lysozyme fusion construct was used in this study^8^. A FLAG epitope was fused to the N-terminus. T4 lysozyme was connected to the β_2_AR at position D29^1.28^ with two alanine residues. The flexible ICL3 (S236-K263) was removed and the C-terminus of the receptor was truncated at position K348. The resulting T4L-β_2_AR construct was expressed in *Sf*9 insect cells with BestBac expression systems. Purification of T4L-β_2_AR and Nb6B9 were performed according to the methods described previously^8^. Briefly, Nb6B9 was purified by nickel affinity chromatography followed by size exclusion chromatography. T4L-β_2_AR was solubilized with dodecylmaltoside and purified by M1-FLAG affinity chromatography followed by alprenolol functional chromatography. The elution of the alprenolol column was loaded to the M1-FLAG affinity column for ligand exchange to c-Epi and detergent exchange to L-MNG. The purified T4L-β_2_AR was incubated with Nb6B9 overnight with a 1:1.5 molar ratio. The excess Nb6B9 was removed by a final size exclusion chromatography with the buffer containing 20 mM HEPES, pH 7.5, 100 mM NaCl, 0.01% MNG, 0.001% CHS, and 100 μM c-Epi. The purified T4L-β_2_AR-Nb6B9-c-Epi was concentrated at 40 mg/mL and aliquoted.

### Crystallization

The T4L-β_2_AR-Nb6B9-c-Epi complex was reconstituted into the lipidic cubic phase with a 1:1 mass ratio of protein to lipid as previously reported^21^. The lipid stock consisted of a 10:1 mass ratio of 7.7 MAG with cholesterol. Crystals were grown using 30–100 nL drops with 1 μL of precipitant solution using a GryphonLCP robot. The crystal condition consisted of 26–31% PEG400, 100 mM MES, pH 6.2–6.7, 75–125 mM ammonium phosphate dibasic, and 1 mM c-Epi. Crystals grew after 1–3 days and were harvested for data collection.

### Data collection and structure determination

The diffraction data were collected at SPring-8 beamline BL32XU. The micro-focused beam with 10 μm × 15 μm size and 1.0 Å wavelength was used for automatic data collection^22^. For each crystal, a 10° dataset was collected with 0.1° oscillation per frame. Automatic data processing was performed by KAMO^23^. Fifty-eight crystals were merged to generate the final 3.1 Å T4L-β_2_AR-Nb6B9-c-Epi dataset and 43 crystals were merged to generate the final 3.4 Å T4L-β_2_AR-Nb6B9-c-ISO dataset. The structure of the T4L-β_2_AR-Nb6B9-c-Epi complex was solved by molecular replacement method with PHENIX^24^ and T4L-β_2_AR-Nb6B9-Epinephrine structure (PDE code: 4LDO) as the search model. Structure refinement was carried out with PHENIX and COOT. Molprobity^25^ was used to validate the final structure. The statistics for data collection and structure refinement were summarized in Supplementary Table 7. The structure figures were prepared using PyMol (The PyMOL Molecular Graphics System, Schrodinger, LLC).

The isomorphous difference map was calculated using FFT^26^ in CCP4^27^. In brief, the T4L-β_2_AR-Nb6B9-c-Epi data and the T4L-β_2_AR-Nb6B9-Epi data were set to the same scale and a Fo-Fo difference map was calculated by subtracting the T4L-β_2_AR-Nb6B9-Epi from the T4L-β_2_AR-Nb6B9-c-Epi data.

Normalized b-factor was calculated by dividing the b-factor of each residue by the overall b-factor of the receptor. To visualize the b-factor change, the residue is colored blue if the normalized b-factor is smaller in the β_2_AR-Nb6B9-c-Epi structure than in the β_2_AR-Nb6B9-Epi structure, and colored in red if the normalized b-factor is larger in the β_2_AR-Nb6B9-c-Epi structure than in the β_2_AR-Nb6B9-Epi structure. The darkness of blue or red color correlates with how large the normalized b-factor difference is between the two structures.

### Radioligand binding

The wild-type and mutated human β_2_AR and β_1_AR constructs were cloned into pFastbac vector and expressed in Sf9 cells with a Bac-to-Bac expression system. The cell membrane was isolated and resuspended with a binding buffer consisting of 20 mM HEPES, pH 7.5, and 100 mM NaCl. For competition binding assays, the diluted membrane was incubated for 2 h with various concentrations of cold ligands and 2 nM [^3^H]DHA in the cold binding buffer containing 0.5% BSA to a final volume of 500 μL. The membrane filtration was performed with Brandel 48-well harvester and the collected filter papers and membranes were incubated with OptiPhase HiFafe 3 liquid scintillation cocktail. The Microbeta2 scintillation counter was used for radioactivity counting. The competition binding curves were fitted by GraphPad Prism 6.0 (GraphPad LLC, CA).

### Cloning

The human β_1_AR, β_2_AR, α1A, α1B, β_1_AR_mut4, β_2_AR_mut4, and the murine α2A receptor were fused to the PK1 sequence, the α2B and α2C receptor to ARMS2-PK2 and all cloned to pCMV (DiscoverX, Eurofins) for β-arrestin-2 recruitment assays, respectively, using polymerase chain reaction and Gibson Assembly (New England Biolabs)^28^. Sequence integrity was verified by DNA sequencing (Eurofins Genomics). The β_1_AR/β_2_AR chimeras were generated by switching the N-terminus of the receptors as well as 55 residues between W^1.31^ and the C-terminus of the receptor. The detailed sequences were shown in Supplementary Figure 9, which is modified from a previous publication from our group^7^.

### Radioligand binding assay with membranes from HEK cells

Binding affinities towards the human β_1_AR and β_2_AR were determined as described previously^29,30^. In brief, membranes were prepared from HEK293T cells transiently transfected with the cDNA for β_1_AR and β_2_AR (obtained from the cDNA resource center, www.cdna.org). Receptor densities (B_max_ value) and specific binding affinities (*K*_*D*_ value) for the radioligand [³H]CGP12,177 (specific activity 51 Ci/mmol, PerkinElmer, Rodgau, Germany) were determined as 4.3 ± 1.1 pmol/mg protein and 0.125 ± 0.032 nM for β_1_AR and 2.7 ± 0.4 pmol/mg protein and 0.080 ± 0.011 nM for β_2_AR, respectively. Competition binding experiments were performed by incubating membranes in binding buffer (25 mM HEPES, 5 mM MgCl_2_, 1 mM EDTA, and 0.006% bovine serum albumin at pH 7.4) at a final protein concentration of 2–6 µg/well, together with the radioligand (final concentration 0.2–0.3 nM) and varying concentrations of the competing ligands for 60 min at 37 °C. Non-specific binding was determined in the presence of unlabeled CGP12,177 at a final concentration of 10 µM. Protein concentration was established using the method of ref. ^31^.

The resulting competition curves were analyzed by nonlinear regression using the algorithms implemented in PRISM 8.0 (GraphPad Software, San Diego, CA) to provide an IC_50_ value, which was subsequently transformed into a *K*_*i*_ value employing the equation of Cheng and Prusoff ^32^. Mean *K*_*i*_ values (±SEM) were derived from 3 to 14 experiments, each performed in triplicates.

### The binding kinetics assay

The binding kinetics assays were performed as previously described in ref. ^7^. In brief, off-rate measurements in membranes containing the target receptors (the human β_1_AR, β_2_AR as well as the β_1_AR_in_/β_2_AR_out_ and β_2_AR_in_/β_1_AR_out_ chimeras) were pre-incubated with 0.1–0.5 nM [^3^H]DHA for 1 h at RT. [^3^H]DHA dissociation rates were initiated with the addition of excess propranolol (50 μM) and samples were subjected to rapid filtration at various times. Association rates were determined by incubating membranes with [^3^H]DHA (ranging from 0.1–0.5 nM concentration) in the absence or presence of three different concentrations of (3, 10, and 30 μM NorEpi for β_2_AR; 0.3, 1, and 3 μM NorEpi for β_1_AR; 3, 10, and 30 μM NorEpi for β_1_AR_in_/β_2_AR_out_; 0.3, 1, and 3 μM NorEpi for β_2_AR_in_/β_1_AR_out_; 1, 3, and 10 μM c-NorEpi on β_2_AR; 3, 10, and 30 μM c-NorEpi for β_1_AR; 1, 3, and 10 μM c-NorEpi on β_1_AR and β_2_AR; 3, 10, and 30 μM c-NorEpi for β_1_AR_in_/β_2_AR_out_; 1, 3, and 10 μM c-NorEpi on β_2_AR_in_/β_1_AR_out_; 0.3, 1, and 3 μM Epi for β_2_AR; 1, 3, and 10 μM Epi on β_1_AR; 0.3, 1, and 3 μM Epi for β_2_AR_in_/β_1_AR_out_; 3, 10, and 30 μM Epi for β_1_AR_in_/β_2_AR_out_; 3, 10, and 30 μM; c-Epi for β_1_AR; 0.1, 0.3, and 1 μM; c-Epi for β_2_AR; 3, 10, and 30 μM c-Epi for β_2_AR_in_/β_1_AR_out_; 3, 10, and 30 μM c-Epi for β_1_AR_in_/β_2_AR_out_) the catecholamine. Aliquots were removed at various times and subjected to rapid filtration. Membranes containing bound [^3^H]DHA were harvested by filtering through GF/C Unifilter^TM^ (Perkin Elmer) and counted on a Top Count^TM^ (Perkin Elmer) scintillation counter. Binding kinetics were calculated using the Kinetics of Competitive Binding fit in GraphPad Prism 6.0 (GraphPad LLC, CA). The kinetics parameters of [^3^H]DHA used in the analysis were derived from a previous study^7^.

### β-Arrestin-2 recruitment and G protein IP-one assay

Determination of receptor-stimulated β-arrestin-2 recruitment was performed applying the PathHunter assay (DiscoverX, Birmingham, UK), which is based on the measurement of fragment complementation of β-galactosidase as described in ref. ^33^. In detail, HEK293T cells stably expressing the enzyme acceptor (EA) tagged β-arrestin-2 fusion protein were transfected with the cDNA for β_1_AR, β_2_AR, β_1_AR_mut4, β_2_AR_mut4, α1A, α1B, or α2A receptor each fused to the ProLink-PK1 fragment for enzyme complementation and transferred into 384 well microplates. α2B and α2C receptors fused to the ProLink-ARMS2-PK2 fragment were treated analogously. Measurement started by incubating cells with agonist for 90 min (β_1_AR, β_2_AR, β_2_AR_mut4, α2B, or α2C, respectively), 180 min (β_1_AR_mut4, α1B, or α2A, respectively), or 300 min (α1A), respectively. Chemoluminescence was monitored with a Clariostar plate reader (BMG, Ortenberg, Germany) and analyzed by normalizing the raw data relative to basal activity (0%) and the maximum effect of Norepi (100%). Four to 21 repeats in duplicate were analyzed by applying the algorithms for four-parameter nonlinear regression implemented in Prism 8.0 (GraphPad LLC, CA) to get dose-response curves representing EC_50_ and E_max_ value. Bias calculations were performed as described by ref. ^17^.

The determination of receptor-mediated G protein signaling by β_1_AR and β_2_AR was performed by applying an IP accumulation assay (IP-One HTRF®, Cisbio, Codolet, France) according to the manufacturer’s protocol and in analogy to previously described protocols^18^. In brief, HEK 293 T cells were co-transfected with the cDNA for β_1_AR or β_2_AR and the hybrid G protein Gαqs (Gαq protein with the last five amino acids at the C-terminus replaced by the corresponding sequence of Gαs (gift from The J. David Gladstone Institutes, San Francisco, CA), respectively and transferred into 384 well microplates. Cells were incubated with an agonist for 120 min and accumulation of the second messenger was stopped by adding detection reagents (IP1-d2 conjugate and Anti-IP1cryptate TB conjugate). After 60 min, TR-FRET was measured with a Clariostar plate reader. FRET-signals from four to 14 repeats in duplicates were normalized to vehicle (0%) and the maximum effect of Norepi (100%) and analyzed to get EC_50_ and E_max_ values.

### cAMP accumulation assay

Initial cAMP assays on β_2_AR were performed using clonal selected HEK293 ΔGNAS cell line^34^, stably expressing the cAMP biosensor pink flamido^35^ (encoded on a pcDNA4.0/TO/Zeocin plasmid, generously provided by Dr. Jin Zhang, UCSD). Briefly, cells were treated with 4 μg/mL doxycycline to induce overexpression of the cAMP biosensor 48 h prior to measurement of cAMP. Cells were gently harvested, washed in Hanks’ Balanced Salt Solution (HBSS, Sigma), and seeded onto clear bottom poly-d-lysine coated, black 96-well polystyrene assay plate (Costar) at ~2 × 10^6^ cells per well. Cells were treated with a dilution series of catecholamine (10^−4^–10^−10^ M) in HBSS buffer with 20 mM HEPES, pH 7.8, 600 μM 3-Isobutyl-1-methylxanthine (IBMX), and 3 mM ascorbic acid. Real-time fluorescence measurements were collected immediately following agonist application: excitation 535 nm, emission 612 nm, integration time 40 µs, bottom read, a kinetic interval of 13 s, and a delay of 10 ms using a fluorescence plate reader (BioTek, Winooski, VT). The cAMP accumulation was monitored for 17.5 min. The fluorescence emission data were fitted to a single exponential to obtain rate constants (from *t* = 5 s to *t* = 305 s) for each agonist concentration using Prizm (GraphPad LLC, CA). Rate constants were plotted as a function of catecholamine concentration and fitted to a logistics curve using Prism.

Subsequent cAMP assays on β_1_AR and β_2_AR were performed in *Sf9* cells. Briefly, cells were infected with baculoviruses for the cAMP biosensor pink flamido, together with either β_1_AR or β_2_AR. Twenty-four hours following infection, cells were gently harvested, washed in HBSS, and seeded onto clear bottom poly-d-lysine coated, black 96-well polystyrene assay plate (Costar) at ~1 × 10^6^ cells per well. Cells were treated with a dilution series of catecholamine (10^−4^–10^−10^ M) in HBSS buffer with 20 mM HEPES, pH 7.8, 600 μM IBMX, and 3 mM ascorbic acid. Real-time fluorescence measurements were collected immediately following agonist application: excitation 535 nm, emission 612 nm, integration time 40 µs, bottom read, a kinetic interval of 13 s, and a delay of 10 ms using a fluorescence plate reader (SpectraMax M5, Molecular Devices, CA). The cAMP accumulation was monitored for 10 min. The fluorescence emission data were fitted to a single exponential to obtain rate constants (from *t* = 5 s to *t* = 305 s) for each agonist concentration using Prizm (GraphPad LLC, CA). Rate constants were plotted as a function of catecholamine concentration and fitted to a logistics curve using Prism. All statistical analysis for EC_50_ and E_max_, as well as relative effectiveness and bias, was performed using Prism. Relative effectiveness and Bias calculations were performed as described by ref. ^17^.

### [^35^S]GTPγS binding assays

Membranes were prepared from *Sf*9 cells expressing Gα_s_β_1_γ_2_ and β_2_AR. Membranes (~15 μg) were pretreated with GDP (final assay concentration of 1 μM) in a GTPγS assay buffer (20 mM HEPES, pH 7.4, 100 mM NaCl, 10 mM MgCl_2_, and 1 mM ascorbic acid) and different concentrations of agonist (Epi, c-Epi, NorEpi, c-NorEpi, Iso or c-Iso) for 10 min at room temperature before adding [^35^S]GTPγS (for a final concentration of 0.1 nM). The assay was incubated at 30 ^o^C for 30 min before stopping by rapid filtration through GF/B Unifilter plates (Whatman) and washing with ice-cold assay buffer. Filter plates were dried before adding Microscint 0™ and counting bound [^35^S]GTPγS (Perkin Elmer) using a TopCount™ (Perkin Elmer). Data were analyzed using Prizm 6.0 (GraphPad, LLC, CA). Figures show the combined results from three separate experiments performed in duplicate.

### Unbiased simulations of receptor-ligand complexes

Simulations of β_2_AR were based on the c-Epi bound crystal structure described in this manuscript and the epinephrine-bound crystal structure (PDB entry 4LDO)^8^. Simulations of β_1_AR were based on a BI-167107 bound crystal structure of β_1_AR (PDB entry 7BU7)^7^. For the simulations of β_1_AR, BI-167107 was replaced with either Epi or c-Epi by structurally aligning the crystal structures β_1_AR and the respective β_2_AR and transferring the coordinates of the ligands. Mutations to β_1_AR were introduced utilizing Maestro (Schrödinger, LCC, New York, NY, 2018) while selecting the rotamers with the highest probability and at the same time, resembling the respective conformation in the c-Epi bound β_2_AR crystal structure.

Coordinates were prepared by removing the nanobody and the T4L fusion protein. Only crystal waters within or close to the receptor were retained. Prime (Schrödinger, LCC, New York, NY, 2018) was used to model missing side chains. Hydrogen atoms were added, and the protein chain termini were capped with the neutral acetyl and methyl amide groups.

Except for D^2.50^, E^3.41^, and D^3.49^, all titratable residues were left in their dominant protonation state at pH 7.0. Previous studies suggested that residues D^2.50^ and D^3.49^ for β_2_AR are protonated in the active state^36,37^, and residue E^3.41^ directly contacts the lipid interface and, therefore, will also exist predominantly in its protonated state^38,39^. We thus protonated these three residues in our simulations. Epi and c-Epi were protonated at the secondary amine allowing the formation of the canonical salt bridge to D^3.32^ conserved in aminergic GPCRs.

The prepared protein structures were then aligned to the Orientation of Proteins in Membranes (OPM)^40^ structure of active β_2_AR (PDB entry 3SN6) and internal water was added utilizing Dowser^41^. Each complex was inserted into a pre-equilibrated bilayer of palmitoyl-oleoyl-phosphatidylcholine (POPC) lipids using Dabble, a simulation preparation software (Betz, R. Dabble (v.2.6.3). 10.5281/zenodo.836914 (2017)). Sodium and chloride ions were added to neutralize each system at a concentration of approximately 150 mM. The final box dimensions were approximately 85 × 75 × 85 Å^3^.

We used the CHARMM36m^42–46^ parameter set for protein molecules, lipid molecules, and salt ions and the CHARMM TIP3P model for water. Parameters for Epi and c-Epi were generated using the CHARMM General Force Field (CGenFF)^47–49^ with the ParamChem server (paramchem.org) version 1.0.0.

We performed the simulations using the CUDA version of PMEMD (particle-mesh Ewald molecular dynamics) in AMBER16^50^. We performed six independent simulations under each condition. Each simulation was equilibrated independently as follows: Three rounds of minimization were performed, each consisting of 500 iterations of steepest descent minimization, followed by 500 iterations of conjugate gradient descent minimization, with harmonic restraints of 10.0, 5.0, and 1.0 kcal mol^−1^ Å^−2^ placed on the protein and lipids, respectively. Systems were heated from 0 to 100 K in the NVT ensemble over 12.5 ps and then from 100 to 310 K in the NPT ensemble over 125 ps, using 10.0 kcal mol^−1^ Å^−2^ harmonic restraints applied to lipid and protein-heavy atoms. Systems were then equilibrated at 310 K in the NPT ensemble at 1 bar, with harmonic restraints on all protein-heavy atoms for 10 ns. Starting at 5.0 kcal mol^−1^ Å^−2^, the restraints were reduced in a stepwise fashion by 1.0 kcal mol^−1^ Å^−2^ every 2 ns. This was followed by additional 20 ns of equilibration with again harmonic restraints on all protein-heavy atoms. Starting at 1.0 kcal mol^−1^ Å^−2^, the restraints were reduced in a stepwise fashion by 0.1 kcal mol^−1^ Å^−2^ every 2 ns. Production simulations were performed in the NPT ensemble at 310 K and 1 bar, using a Langevin thermostat for temperature coupling and a Monte Carlo barostat for pressure coupling. These simulations used a 4 fs time step with hydrogen mass repartitioning^51^. Bond lengths to hydrogen atoms were constrained using SHAKE^52^. Periodic boundary conditions were applied. Non-bonded interactions were cut off at 9.0 Å, and long-range electrostatic interactions were computed using particle-mesh Ewald (PME)^53^ with an Ewald coefficient of approximately 0.31 Å and an interpolation order of four. The fast Fourier transform (FFT) grid size was chosen such that the width of a grid cell was ~1 Å.

During production simulations, all residues within 5 Å of the G protein interface at β_2_AR were restrained to the initial structure using 5.0 kcal mol^−1^ Å^−2^ harmonic restraints applied to non-hydrogen atoms. Using such restraints instead of the intracellular binding partner reduces the overall system size, enabling faster simulation, while ensuring that the receptor maintains an active conformation throughout the simulation.

Trajectory snapshots were saved every 200 ps during production simulations. Analysis of the trajectories was performed using Visual Molecular Dynamics (VMD)^54^, CPPTRAJ^55^, and GetContacts (https://getcontacts.github.io/). Root-mean-square fluctuations (rmsf) were calculated to an average structure omitting the first 500 ns of each simulation trajectory to avoid including any initial relaxation or equilibration of the system. Trajectories were aligned to the initial crystal structure on all transmembrane helix alpha carbons. For each simulation condition, an average structure was generated by considering trajectory snapshots from all simulations under that condition. The rmfs for each alpha carbon was then calculated for each trajectory under that condition relative to this average position using CPPTRAJ. Visualization was performed using the PyMOL Molecular Graphics System, Version 2.1.1 (Schrödinger, LLC). Plots were created using Matplotlib 3.0.2^56^.

### Unbiased simulations of ligands in solution

Parameter topology and coordinate files were built up using the tleap module of AMBER18^57^. For the simulations, the general AMBER force field (GAFF)^58^ was used for Epi and c-Epi. The ligands were solvated in a truncated octahedron with a minimum solute-to-wall distance of 25 Å. The systems were neutralized with one chloride ion. The TIP3P water model^59^ was applied. Parameters for Epi and c-Epi were assigned using antechamber^57^. The structure of Epi and c-Epi were optimized using Gaussian 16^60^ at the B3LYP/6-31 G(d) level of theory and charges were calculated at HF/6-31 G(d) level of theory. Subsequently, atom point charges were assigned according to the RESP procedure^61^. A formal charge of +1 was assigned to Epi and c-Epi.

We performed the simulations using the CUDA version of PMEMD in AMBER18^57^. Each simulation system was energy minimized 2500 iterations of steepest descent minimization, followed by 7500 iterations of conjugate gradient descent minimization and equilibrated in the NVT ensemble at 310 K for 1 ns followed by the NPT ensemble for 1 ns with harmonic restraints of 10.0 kcal mol^–1^ on the ligand. In the NVT ensemble, the Langevin thermostat was used. In the NPT ensemble, the Monte Carlo barostat was applied. The system was further equilibrated for 12 ns with restraints on the ligand atoms. Here, the restraints were reduced every 3 ns in a stepwise fashion to be 5.0, 1.0, 0.5, and 0.1 kcal mol^−1^, respectively. Productive simulations were performed using periodic boundary conditions and a time step of 2 fs. Bond lengths to hydrogen atoms were constrained using SHAKE^52^. Long-range electrostatic interactions were computed using the PME^53^ method. Non-bonded interactions were cut off at 8.0 Å.

Trajectory snapshots were saved every 50 ps during production simulations. Trajectories were visualized using VMD^54^ and cluster based on the ligand atoms using the CPPTRAJ^55^ module of AMBER18. Visualization was performed using PyMOL Molecular Graphics System, Version 2.1.1 (Schrödinger, LLC). Plots were created using Matplotlib 3.0.2^56^.

### Reporting summary

Further information on research design is available in the Nature Portfolio Reporting Summary linked to this article.

## Supplementary information

Supplementary Information

Peer Review File

Reporting Summary

## Data Availability

The coordinates and structural factors of T4L-β_2_AR/NB6B9/c-Epi and T4L-β_2_AR/NB6B9/c-ISO structures have been deposited into Protein Data Bank under the accession code 7XKA (T4L-β_2_AR/NB6B9/c-Epi structure) and 7XK9 (T4L-β_2_AR/NB6B9/c-ISO structure), respectively. Source data are provided with this paper.

## Source data

Source Data
