# Author Correction: Constrained catecholamines gain β_2_AR selectivity through allosteric effects on pocket dynamics

**DOI:** 10.1038/s41467-023-38820-y

**Published:** 2023-05-24

**Authors:** Xinyu Xu, Jeremy Shonberg, Jonas Kaindl, Mary J. Clark, Anne Stößel, Luis Maul, Daniel Mayer, Harald Hübner, Kunio Hirata, A. J. Venkatakrishnan, Ron O. Dror, Brian K. Kobilka, Roger K. Sunahara, Xiangyu Liu, Peter Gmeiner

**Affiliations:** 1 State Key laboratory of Membrane Biology, Tsinghua-Peking Center for Life Sciences, School of Pharmaceutical Sciences, Tsinghua University, Beijing, 100084 China; 2 Beijing Frontier Research Center for Biological Structure, Beijing Advanced Innovation Center for Structural Biology, Tsinghua University, Beijing, 100084 China; 3 Department of Chemistry and Pharmacy, Medicinal Chemistry, Friedrich-Alexander University Erlangen-Nurnberg, Nikolaus-Fiebiger-Straße 10, 91058 Erlangen, Germany; 4 Department of Pharmacology, University of California San Diego School of Medicine, 9500 Gilman Drive, La Jolla, California 92093 USA; 5 Advanced Photon Technology Division, Research Infrastructure Group, SR Life Science Instrumentation Unit, RIKEN/SPring-8 Center, 1-1-1 Kouto Sayo-cho Sayo-gun, Hyogo, 679-5148 Japan; 6 Precursory Research for Embryonic Science and Technology (PRESTO), Japan Science and Technology Agency, 4-1-8 Honcho, Kawaguchi, Saitama 332-0012 Japan; 7 Department of Computer Science, Stanford University, Stanford, CA 94305 USA; 8 Department of Molecular and Cellular Physiology, Stanford University School of Medicine, Stanford, CA 94305 USA; 9 Department of Structural Biology, Stanford University School of Medicine, Stanford, CA 94305 USA; 10 Institute for Computational and Mathematical Engineering, Stanford University, Stanford, CA 94305 USA; 11 Beijing Key Laboratory of Cardiovascular Receptors Research, Peking University, Beijing, China

**Keywords:** X-ray crystallography, Small molecules, G protein-coupled receptors

Correction to: *Nature Communications* 10.1038/s41467-023-37808-y, published online 14 April 2023

The original version of this Article contained an error in Fig. 1c, in which the chemical structure of (R,R)-c-NorEpi was the same as that of the (R,R)-c-Epi. This has been corrected in both the PDF and HTML versions of the Article.

