# Supplementary material for: Constrained catecholamines gain β2AR selectivity through allosteric effects on pocket dynamics
Source: Nat Commun. 2023 Apr 14;14:2138. doi: 10.1038/s41467-023-37808-y (PMC10104803; doi:10.1038/s41467-023-37808-y)
Supplement: Supplementary file 1 — Supplementary Information [file 41467_2023_37808_MOESM1_ESM.pdf]

Supplementary Figures

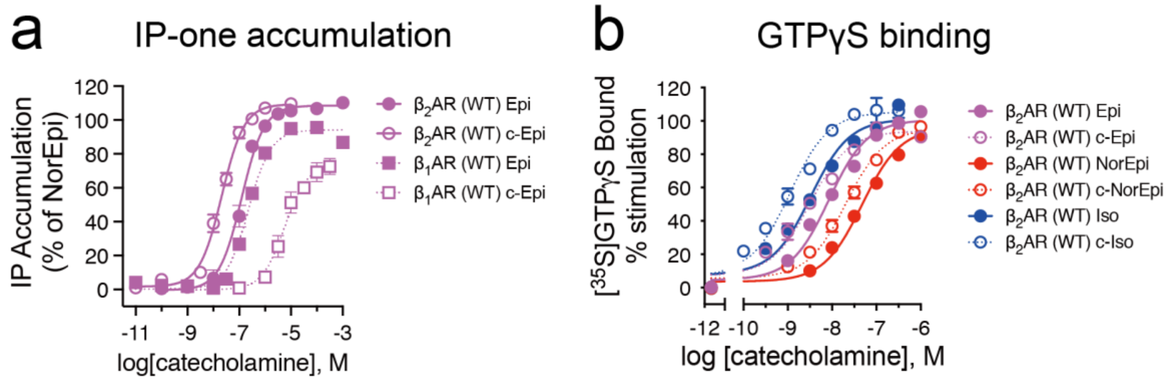

Supplementary Figure 1 | Constrained catecholamines show increased potency to activate the β_2AR compared to non-constrained forms. a, c-Epi has a smaller EC_{50} than Epi for the β_2AR and has a larger EC_{50} than Epi for the β_1AR in the IP-one G protein activation assay. Data are given as mean ± SEM of n=4 (β_1AR c-Epi), n=6 (β_2AR Epi), and n=7 (β_2AR Epi, β_2AR c-Epi) independent experiments. **b**, c-Epi, c-NorEpi and c-ISO show increased potency than Epi, NorEpi and ISO for the β_2AR in $[S^{35}]$GTPγS binding assay. Data are given as mean ± SEM of n=3 (for all samples) independent experiments. Source data are provided as a Source data file.

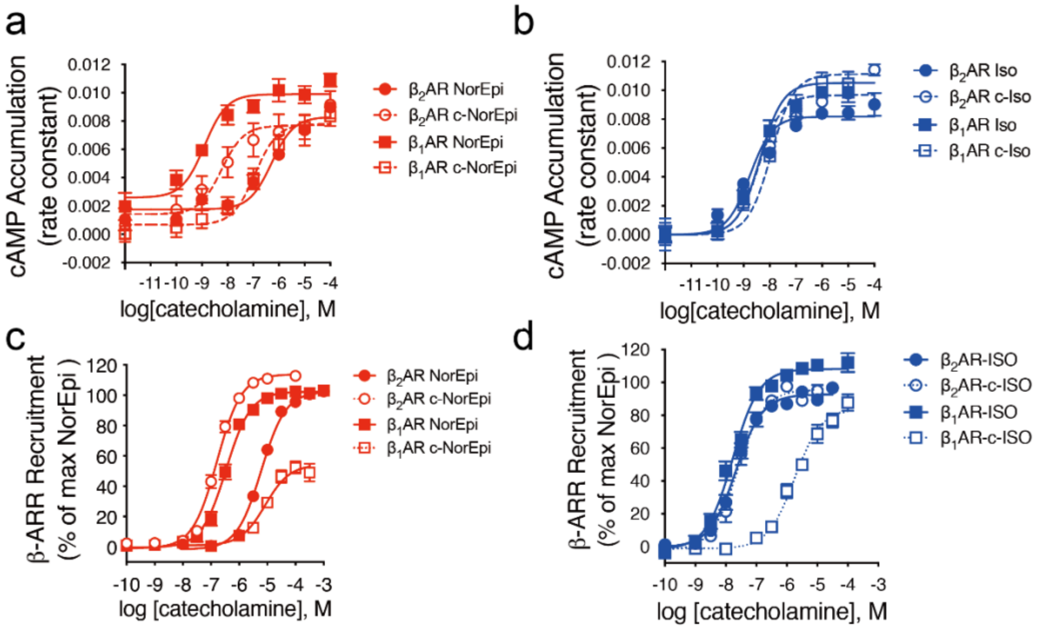

Supplementary Figure 2 | Comparison of NorEpi, c-NorEpi, ISO and c-ISO in cAMP accumulation and arrestin recruitment assay. a, c-NorEpi shows β_2AR selectivity in a cAMP accumulation assay. **b,** ISO and c-ISO shows similar EC_{50} in a cAMP accumulation assay. **c**, while NorEpi is β_1AR-selective in arresin recruitment, c-NorEpi is β_2AR-selective in the same assay. **d**, Compared to ISO, c-ISO shows decreased potency to activate the β_1AR but not β_2AR in arrestin recruitment assay. For a,b, data are given as mean ± SEM of n=3 (for NorEpi, ISO and c-ISO), and n=9 (for c-NorEpi) independent experiments. For **c,** data are given as mean ± SEM of n=9 (for NorEpi), and n=13 (for c-NorEpi); for **d,** data are given as mean ± SEM of n=9 (β_1AR Iso), n=12 (β_2AR Iso), n= 14 (β_1AR c-Iso), and n= 21 (β_2AR c-Iso). Source data are provided as a Source data file.

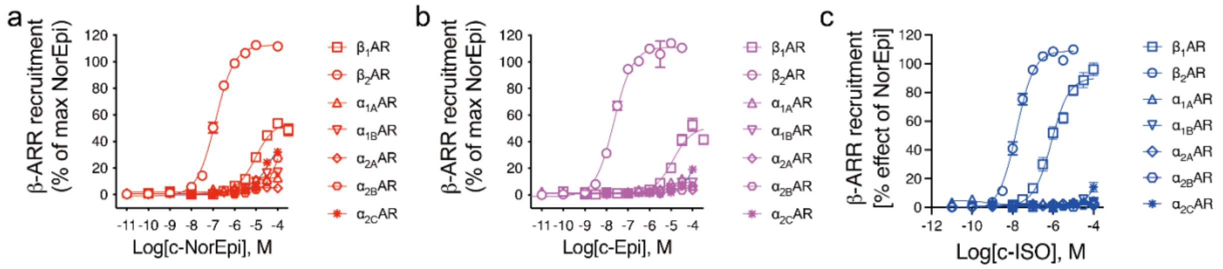

Supplementary Figure 3 | The three constained catecholamines show β_2AR selectivity in a β-arrestin recruitment assay among β_1AR and αARs. Of the 7 adrenoceptors tested, the β_2AR shows highest potency to recruit β-arrestin for c-NorEpi (a), c-Epi (b) and c-ISO (c). Data are given as mean ± SEM of n=4 (for α_{1B}AR, α_{2B}AR, α_{2C}AR), n=6 (α_{1A}AR, α_{2A}AR, for c-NorEpi), n=8 (α_{1A}AR, α_{2A}AR, for c-Epi and c-Iso), n=13 (β_1AR, β_2AR, for c-NorEpi), n=14 (β_1AR for c-Iso), n=15 (β_1AR for c-Epi), and n=21 (β_2AR for c-Epi, c-Iso) independent experiments. Source data are provided as a Source data file.

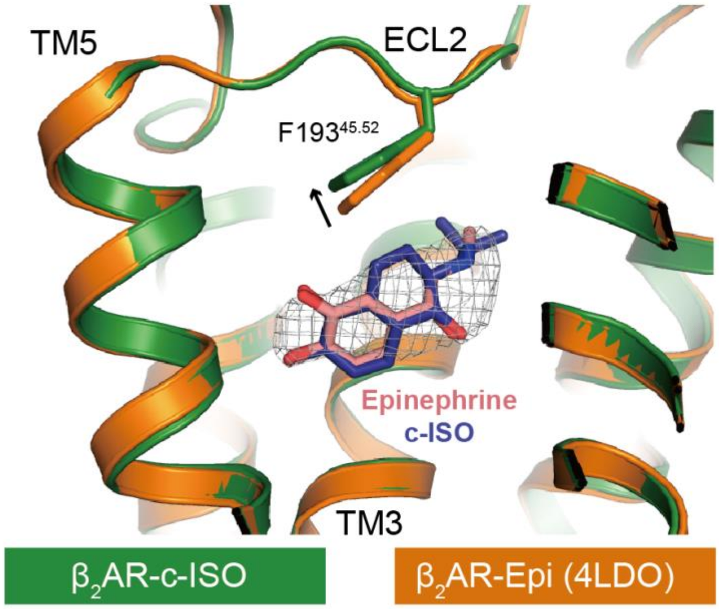

Supplementary Figure 4 | F$^{45.52}$ shows upward movement upon c-ISO binding compared to Epi binding. The grey mesh shows the simulated omit fo-fc density of the c-ISO countered at 3.0 σ.

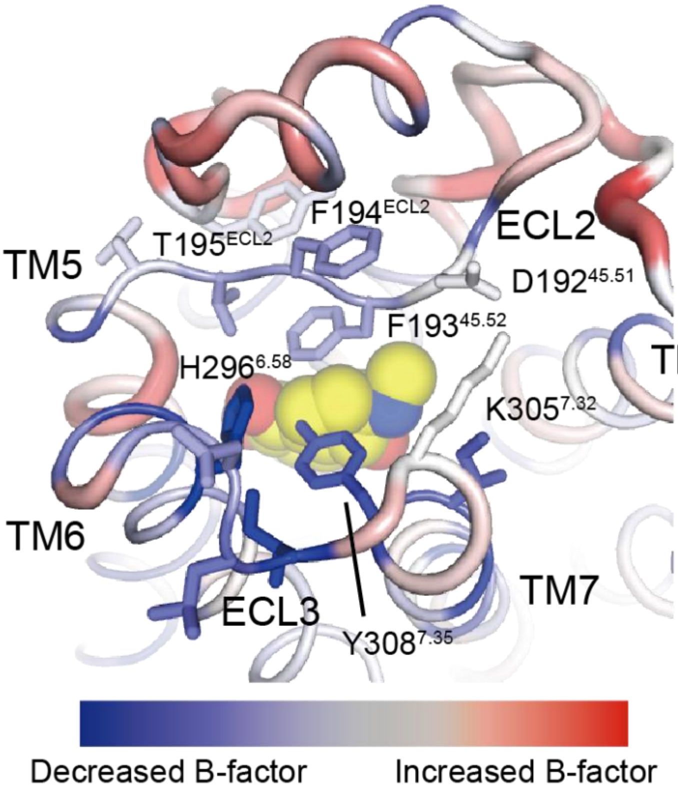

Supplementary Figure 5 | c-Epi binding results in reduced flexibility of the ECL2 and ECL3 compared to Epi bound β_2AR as revealed by decreased normalized B-factors (blue color).

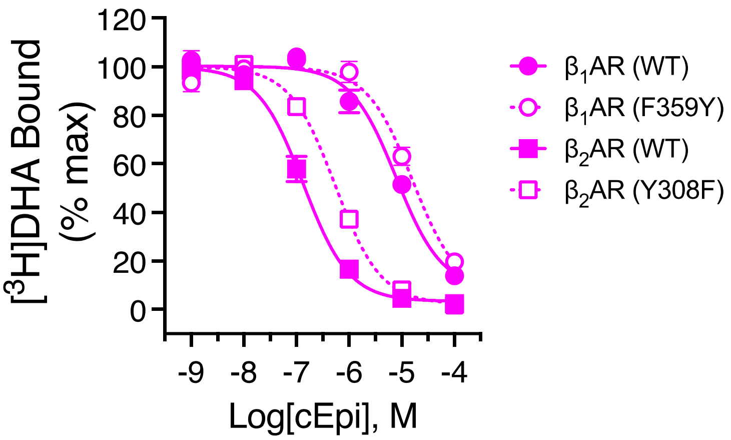

Supplementary Figure 6 | The Y/F$^{7.35}$ difference is not responsible for the c-Epi selectivity towards the β$_2$AR. The Y308$^{7.35}$F mutation slightly decreased c-Epi affinity in the β$_2$AR, while the F359$^{7.35}$Y mutation also slightly decreased c-Epi affinity in the β$_1$AR. Data are given as mean ± SEM of n=4 (for all samples) independent experiments. Source data are provided as a Source data file.

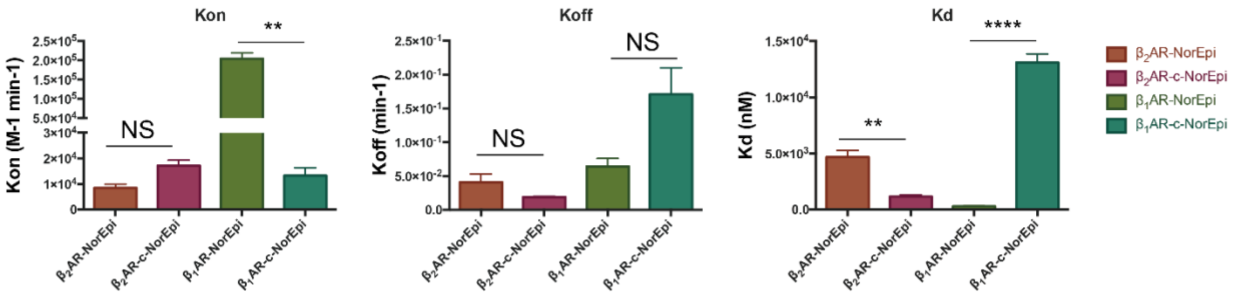

Supplementary Figure 7 | Binding kinetics studies of c-NorEpi and NorEpi on the β_1AR and β_2AR. Statistic analysis of the binding kinetics values for NorEpi and c-NorEpi were performed using 2-way ANOVA analysis. (*: P<0.05, **: P<0.005, ***: P<0.0005, ****: P<0.0001). Data are given as mean ± SEM of n=4 (β_2AR, for NorEpi and c-NorEpi), n=8 (β_1AR, for norEpi), n=5 (β_1AR, for cNorEpi) independent experiments. Source data are provided as a Source data file.

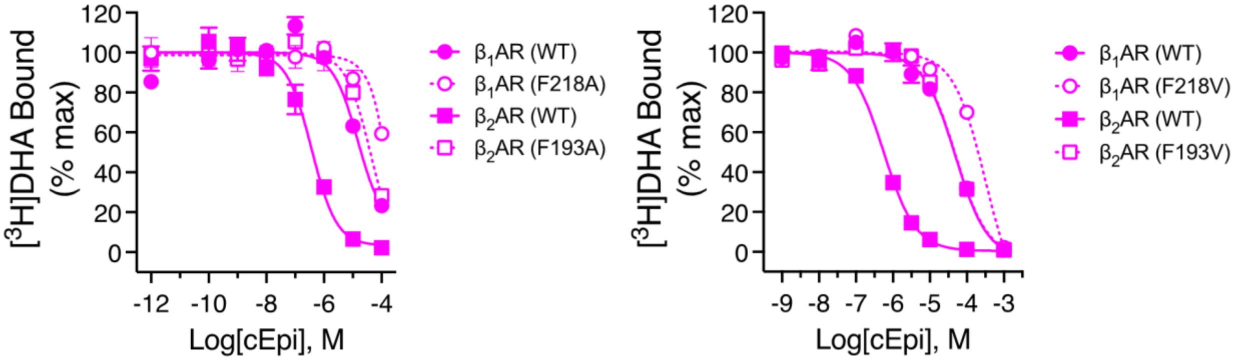

Supplementary Figure 8 | $F^{45.52}$A or $F^{45.52}$V mutation decreases c-Epi affinity in both β_1AR and β_2AR. Data are given as mean ± SEM of n=6 (β_1AR and β_1AR(F218A) and β_2AR and β_2AR(F193A), n=6 (β_1AR and β_1AR(F218V) and β_2AR and β_2AR(F193V) independent experiments. Source data are provided as a Source data file.

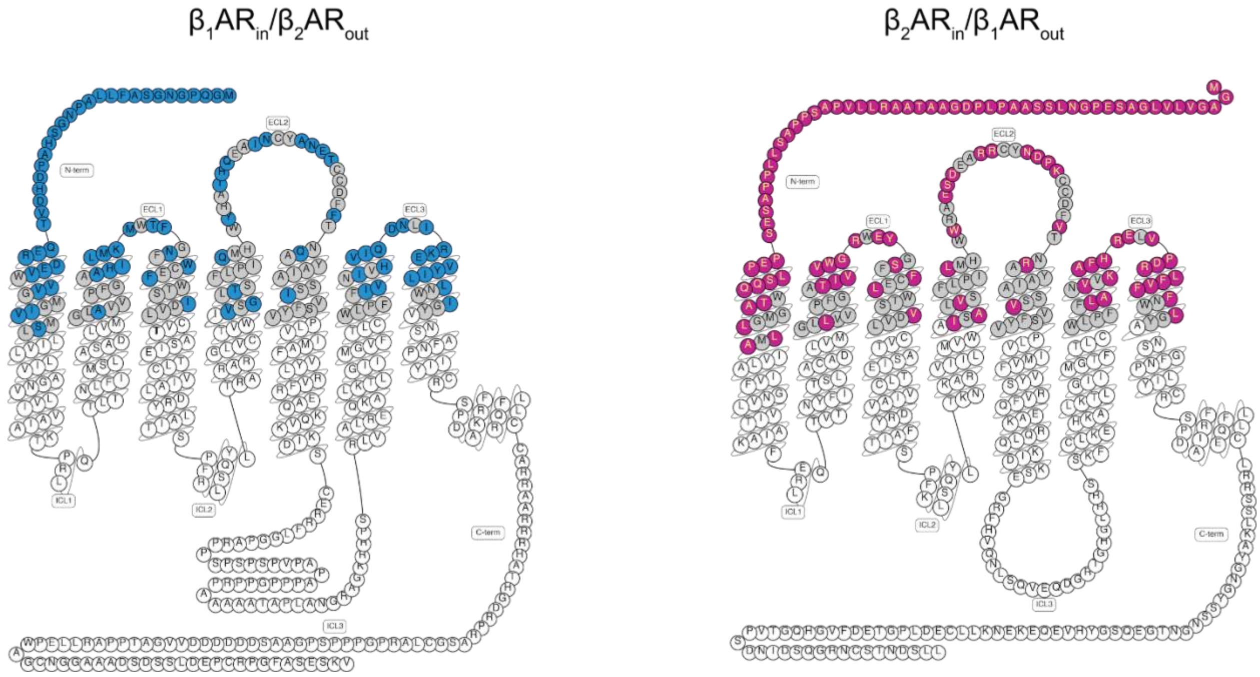

Supplementary Figure 9| Construct design of $\beta_1AR_{in}/\beta_2AR_{out}$ and $\beta_2AR_{in}/\beta_1AR_{out}$ chimeras. The figure is modified from a previous publication[1].

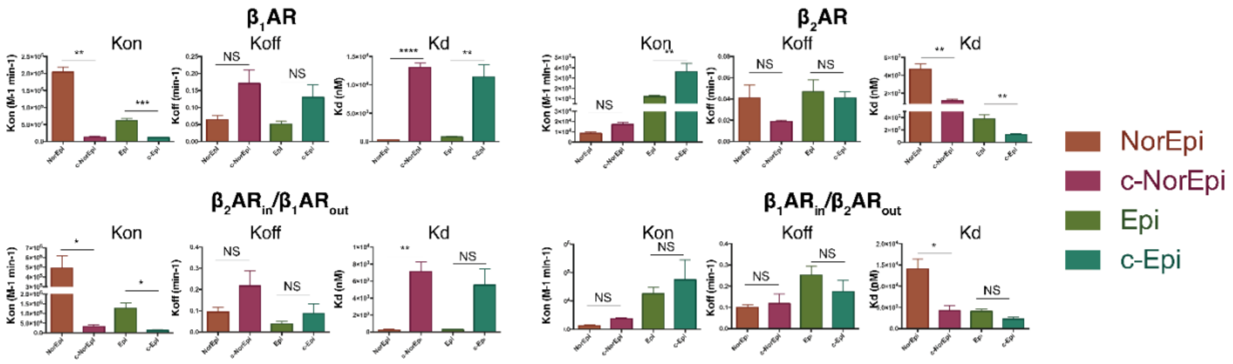

Supplementary Figure 10| Comparison of binding kinetics of Epi, c-Epi, NorEpi, c-NorEpi on β_1AR, β_2AR as well as the $\beta_1AR_{in}/\beta_2AR_{out}$ and $\beta_2AR_{in}/\beta_1AR_{out}$ chimeras. Statistic analysis of the binding kinetics values were performed using 2-way ANOVA analysis. (*: P<0.05, **: P<0.005, ***: P<0.0005, ****: P<0.0001). Data are given as mean ± SEM of n=3 (β_1AR for Epi), n=3(β_2AR for Epi), n=3 (β_1AR for NorEpi), n=5(β_2AR for NorEpi), n=6 (β_1AR for cEpi), n=6 (β_2AR for cEpi), n=5 (β_1AR for cNorEpi), n=5(β_2AR for cNorEpi), n=4 (β_2AR_{in}/ β_1AR_{out}), n=4 (β_1AR_{in}/ β_2AR_{out}) independent experiments. Source data are provided as a Source data file.

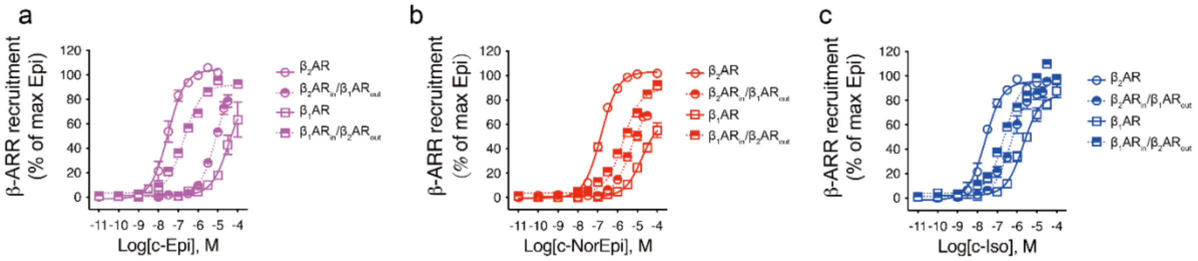

Supplementary Figure 11| Exchanging the extracellular vestibule between the β_1AR and β_2AR results in the change of the EC_{50} values for the constrained catecholamines in a β-arrestin recruitment assay. The β_1AR_{in}/ β_2AR_{out} has smaller EC_{50} than the β_1AR for c-NorEpi (a), c-Epi (b) and c-ISO (c), while the $\beta_2AR_{in}/\beta_1AR_{out}$ has larger EC_{50} than the β_2AR for all three constrained catecholamines. Data are given as mean ± SEM of n=4 (β_1AR for c-Epi), n=5 (β_AAR for c-NorEpi, c-Iso), n=6 (β_2AR for c-Epi, c-Iso), n=8 ($\beta_1AR_{in}/\beta_2AR_{out}$ for c-NorEpi, c-Iso), n=9 (β_2AR for c-NorEpi), n=10 ($\beta_2AR_{in}/\beta_1AR_{out}$ for c-NorEpi, c-Iso), n=11 ($\beta_2AR_{in}/\beta_1AR_{out}$ for c-Epi), and n=12 ($\beta_1AR_{in}/\beta_2AR_{out}$ for c-Epi) independent experiments. Source data are provided as a Source data file.

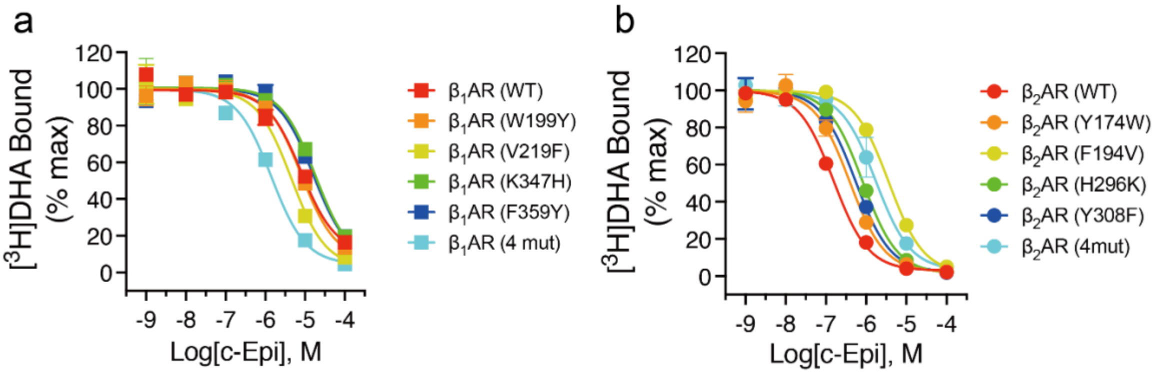

Supplementary Figure 12 | Agonist competition binding curves of c-Epi to β_1AR and β_2AR mutations. a, c-Epi competition curves show leftward shift for β_1AR-V219F and β_1AR-4mut compared to WT β_1AR. **b**, c-Epi competition curves show rightward shift for all four single mutations as well as β_2AR-4mut compared to WT β_2AR. Data are given as mean ± SEM of n=4 (all samples) independent experiments. Source data are provided as a Source data file.

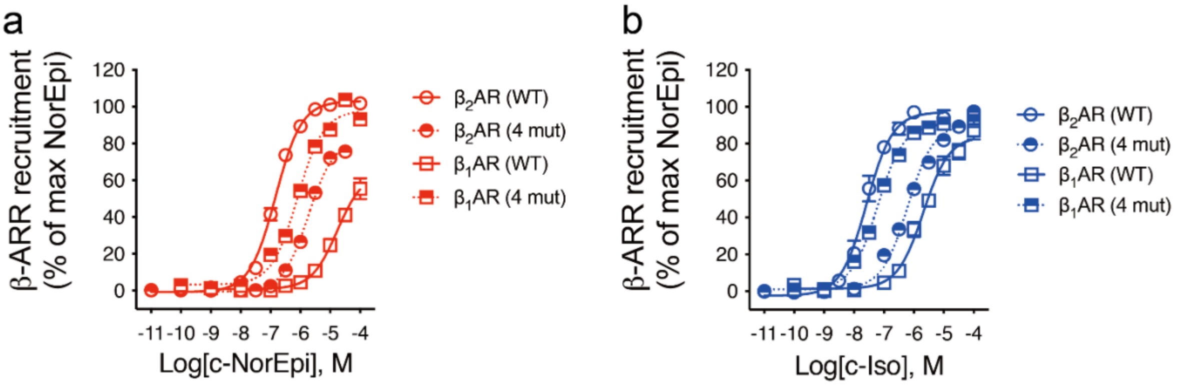

Supplementary Figure 13 | Mutating 4 residues around the $F^{45.52}$ affect c-NorEpi and c-ISO affinities in the β_1AR and β_2AR. a, Mutating the 4 residues surrounding $F^{45.52}$ reduced c-NorEpi affinity for the β_2AR and increased c-NorEpi affinity for the β_1AR in a β-arrestin recruitment assay. **b,** Mutating the 4 residues has similar effects on β_1AR and β_2AR's affinity for c-ISO in β-arrestin recruitment assay. Data are given as mean ± SEM of n=5 (β_1AR (WT), β_1AR (4 mut)), n=6 (β_2AR (WT) for c-Iso), n=7 (β_2AR (4 mut) for c-NorEpi), n=8 (β_2AR (4 mut) for c-Iso), and n=9 (β_2AR (WT) for c-NorEpi) independent experiments. Source data are provided as a Source data file.

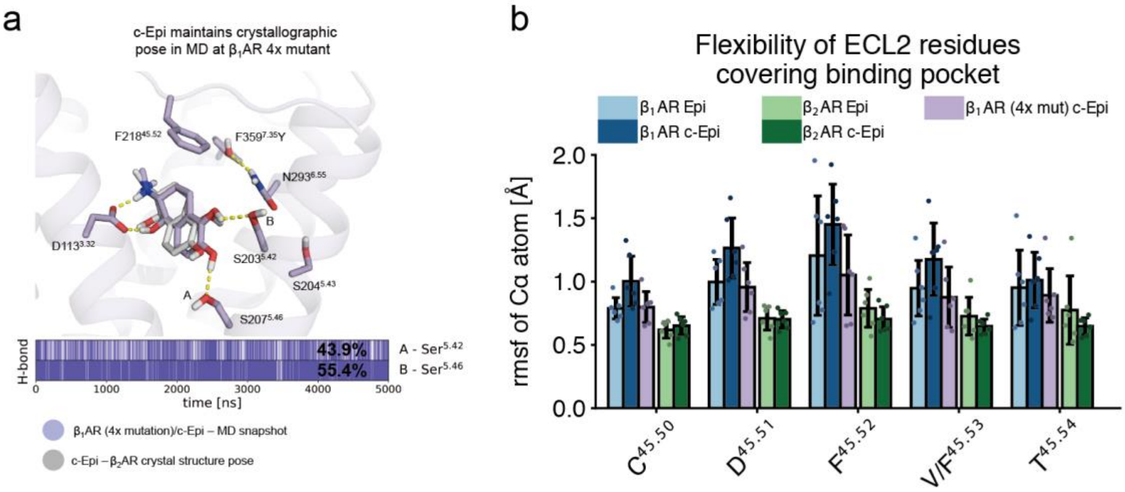

Supplementary Figure 14 | c-Epi maintains its pose in simulations in the β_1AR_4mut and the simulations show reduced ECL2 movement in β_1AR_4mut compared to β_1AR. a, c-Epi maintains its crystallographic pose in the β_1AR_4mut in simulations. A representative frame of the simulations is displayed in purple and the crystallographic pose of c-Epi in gray. The trace plot indicated the presence of the canonical hydrogen bonds to Ser$^{5.42}$ and Ser$^{5.46}$. If colored, the interaction is present, if left blank it is not. The trace is displayed for one representative trajectory. The percentage value is the mean over six simulations, 5 µs each. **b,** The rmsf of the Cα atoms F$^{45.52}$ and its surrounding residues is displayed. The ECL2 flexibility is reduced for the β_1AR_4mut c-Epi condition compared to β_1AR-c-Epi. Data are given as mean ± SEM of 5 independent experiments.

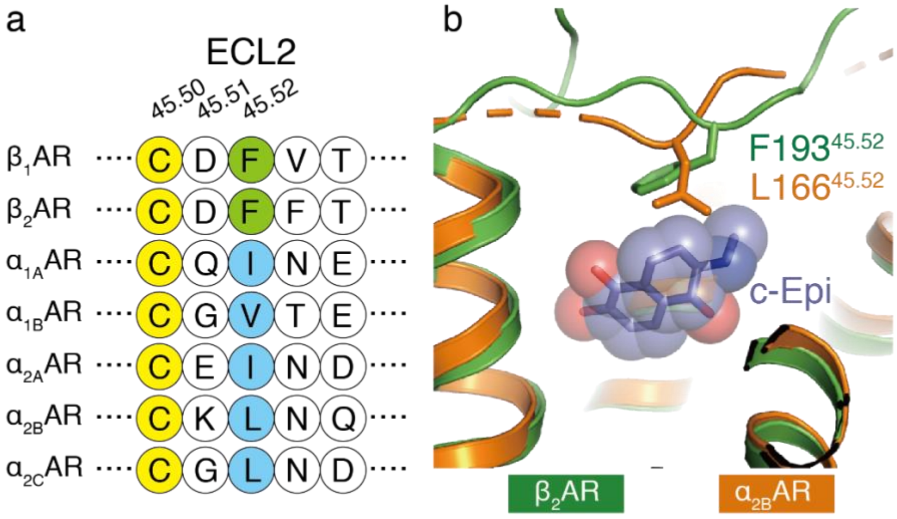

Supplementary Figure 15 | The $F^{45.52}$ is not conserved in the αARs. a, sequence alignment of ECL2 between β_1AR, β_2AR and 5 α-adrenergic receptors (αARs). $F^{45.52}$ is branched-chain amino acids (valine, leucine or isoleucine) in the αARs. **b**, Structure alignment of β_2AR-c-Epi (this study) and α_{2B}AR (PDB: 6K41) suggest a leucine residue at position 45.52 may clash with the connecting carbons of c-Epi.

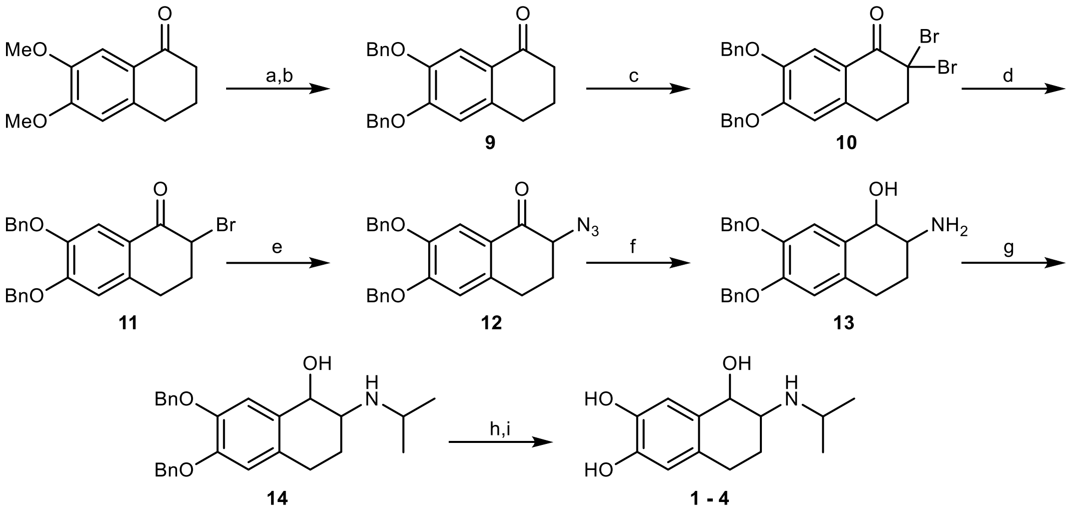

Supplementary Figure 16 | Synthesis of the conformationally restricted catecholamines 1-4. The conformationally restricted catecholamines **1-4** were synthesized from 6,7-dimethoxy-1-tetralone. The reaction sequence started with an exchange of the *O*-methyl substituents by benzyl groups using $AlCl_3$ in toluene[3] and subsequent reaction of the resulting catechol with benzyl bromide in acetone in presence of sodium iodide to give dibenzyloxyketone **9**. Bromination of **9** gave the α,α-dibromo derivative **10**, which could be selectively reduced to the α-bromoketone **11**. Treatment of the synthetic intermediate **11** with sodium azide in DMF gave access to the azidoketone **12**, which was directly reduced with lithium aluminum hydride in 1,2-dichloroethane to afford the aminoalcohol **13** (*cis*/*trans* 1:1). Introduction of an isopropyl group was performed by reductive alkylation. Separation of all four isomers of the resulting products (**14)** on chiral HPLC and subsequent hydrogenolytic debenzylation yielded the four stereoisomers of the final products **1-4**[4]. Reagents and conditions: a) $AlCl_3$, toluene, reflux, 1 h; b) benzyl bromide, K_2CO_3, NaI, acetone, reflux, 16 h; c) Br_2, Et_2O, r.t., 5 min; d) diethyl phosphite, Et_3N, THF, 0 °C to r.t., 72 h; e) NaN_3, HOAc, DMF, r.t., 1 h; f) $LiAlH_4$ in THF, 1,2-DCE, r.t., 16 h; g) acetone, $NaBH(OAc)_3$, 1,2-DCE, r.t., 96 h; h) Chromatography (ChiralPak IC column); i) H_2, Pd/C, MeOH, r.t., 30-90 min.

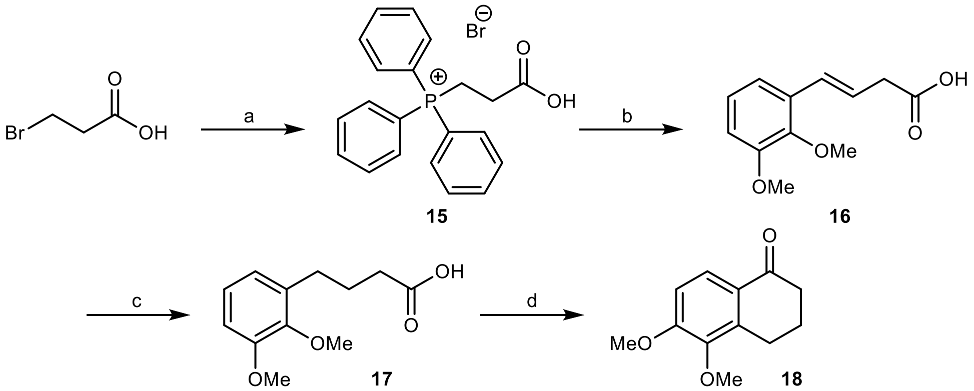

Supplementary Figure 17 | Synthesis of 5,6-dimethoxy-1-tetralone. 5,6-Dimethoxy-1-tetralone was synthesized starting from 3-bromopropanoic acid following a modified literature protocol[5]. Alkylation of triphenylphosphine with 3-bromopropanoic acid in acetonitrile gave the phosphonium bromide **15**, which was subjected to a Wittig-reaction with 2,3-dimethoxybenzaldehyde. Subsequent hydrogenation afforded the cyclization precursor **17**. Upon treatment of **17** with polyphosphoric acid, ring closure was promoted resulting in formation of the building block **18**. Reagents and conditions: a) triphenylphosphine, MeCN, reflux, 12 h; b) NaH, THF, DMSO, r.t., 5 min, then 2,3-dimethoxybenzaldehyde, r.t., 2 h; c) H_2, Pd/C, MeOH, r.t., 2 h; d) polyphosphoric acid, 70 °C, 1 h.

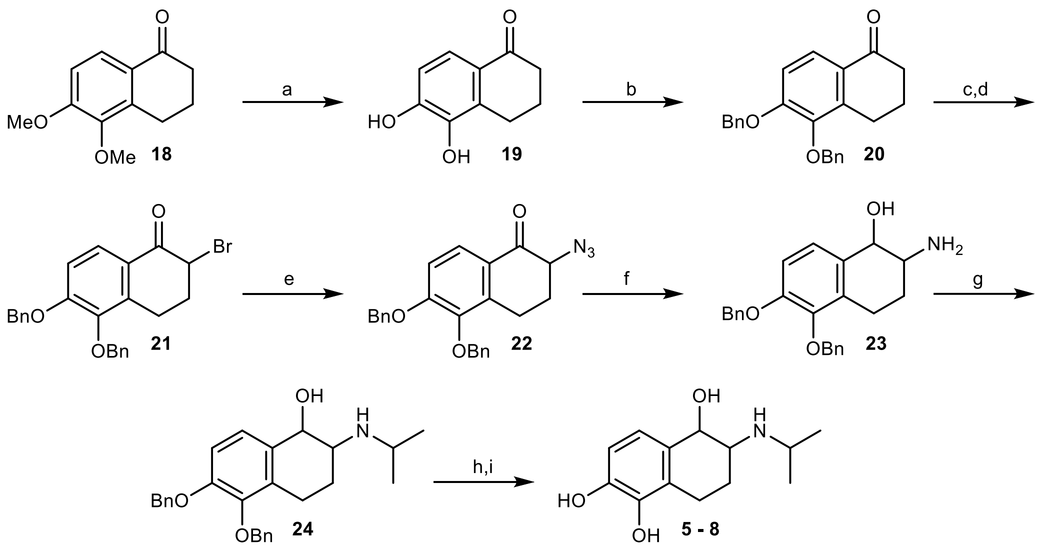

Supplementary Figure 18 | Synthesis of the conformationally restricted catecholamines 5-8. The conformationally restricted catecholamines **5-8** were synthesized from 5,6-dimethoxy-1-tetralone[6,7]. The reaction sequence started by an exchange of the *O*-methyl substituents by benzyl groups using $AlCl_3$ in toluene[3] and subsequent reaction of the resulting catechol **19** with benzyl bromide in acetone in presence of sodium iodide to give dibenzyloxyketone **20**. Bromination of **20** gave the α,α-dibromo intermediate, which could be selectively reduced to the α-bromoketone **21**. Treatment of the synthetic intermediate **21** with sodium azide in DMF gave access to the azidoketone **22**. Reduction of **22** with lithium aluminum hydride resulted in formation of all four isomers of **23** (2:3 *cis*/*trans* ratio). After reductive alkylation of **23**, the four isomers of the resulting isopropyl amines (**24**) were resolved on chiral HPLC to give three fractions: (*S*,*S*)-isomer, a mixture of (*R*,*R*)- and (*R*,*S*)-isomers, and the (*S*,*R*)-isomer. The individual fractions were deprotected by catalytic hydrogenolysis to yield the stereoisomers **7** (*S*,*S*) and **6** (*S*,*R*) as well as a mixture of **8** (*R*,*R*) and **5** (*R*,*S*), which could be separated by preparative HPLC giving access to the pure stereoisomers. Without further information on the stereochemistry, the designation c-ISO refers to the 5,6-(*R*,*R*) isomer. Reagents and conditions: a) $AlCl_3$, toluene, reflux, 1 h; b) benzyl bromide, K_2CO_3, NaI, acetone, reflux, 2 h; c) Br_2, Et_2O, r.t., 1 h; d) diethyl phosphite, Et_3N, THF, 0 °C to r.t., 16 h; e) NaN_3, HOAc, DMF, 0 °C, 3 h; f) $LiAlH_4$ in THF, 1,2-DCE, r.t., 4 h; g) acetone, $NaBH(OAc)_3$, 1,2-DCE, r.t., 16 h; h) preparative HPLC (ChiralPak IC column); i) H_2, Pd/C, MeOH, r.t., 30-90 min, preparative HPLC (C-8 column).

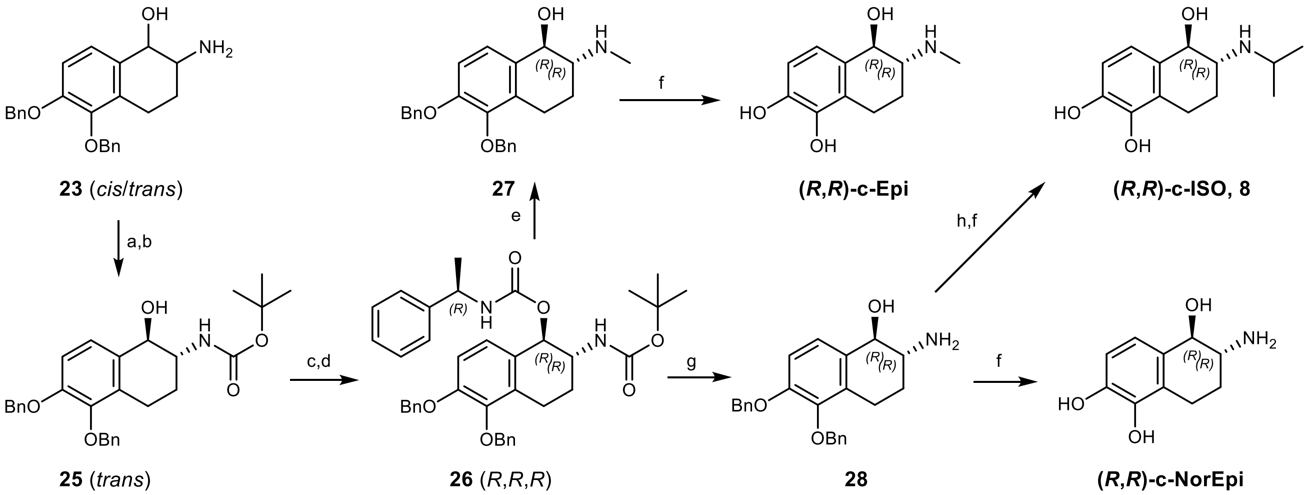

Supplementary Figure 19 | Synthesis of (R,R)-c-Epi, (R,R)-c-NorEpi and (R,R)-c-ISO. (*R*,*R*)-c-ISO, (*R*,*R*)-c-Epi and (*R*,*R*)-c-NorEpi were synthesized from the central building block **26**, which could be readily prepared on a multigram scale. Hence, the aminoalcohol **23** (mixture of four stereoisomers) was *N*-alkoxycarbonylated to give the Boc-protected intermediate **25**. Column chromatography and subsequent recrystallization allowed complete removal of *cis* isomers. Racemic *trans*-**25** was subjected to an organotin-catalyzed addition reaction[8] with chiral methylbenzyl isocyanate yielding a 1:1 mixture of (*R*,*R*,*R*)- and (*S*,*S*,*R*)-dicarbamate (**26**). Repeated recrystallization from toluene/isohexane yielded pure (*R*,*R*,*R*)-**26** (>99.5% *de*), as confirmed by chiral HPLC. TBAF-promoted deprotection of (*R*,*R*,*R*)-**26** yielded the primary amine[9] **28** which could be *N*-isopropylated and *O*-deprotected to afford (*R*,*R*)-c-ISO (**8**) (alternative synthesis). Hydrogenolysis of (*R*,*R*)-**28** resulted in formation of (*R*,*R*)-c-NorEpi. (*R*,*R*)-c-Epi was prepared by lithium aluminum hydride promoted reduction and subsequent debenzylation of (*R*,*R*,*R*)-**26** (enantiomeric excess >98%). Reagents and conditions: a) Boc_2O, *N*,*N*-diisopropylethylamine, CH_2Cl_2, r.t., 18 h; b) recrystallization; c) (*R*)-methylbenzyl isocyanate, Bu_2Sn dilaurate, CH_2Cl_2, r.t., 7 d; d) recrystallization; e) $LiAlH_4$, THF, 90 °C, 1 h ; f) H_2, Pd/C, EtOH, r.t., 1 h; g) TBAF, THF, 90 °C, 3-6 h; h) acetone, $NaBH(OAc)_3$, CH_2Cl_2, r.t., 4 h.

Supplementary Tables

compound		β_1AR[a]		β_2AR[a]		α_{1A}AR[b]		α_{1B}AR[b]		α_{2A}AR[c]		α_{2B}AR[c]		α_{2C}AR[c]	
	sterochemistry	K_i [nM ± S.E.M.]	n[d]	K_i [nM ± S.E.M.]	n[d]	K_i [nM ± S.E.M.]	n[d]	K_i [nM ± S.E.M.]	n[d]	K_i [nM ± S.E.M.]	n[d]	K_i [nM ± S.E.M.]	n[d]	K_i [nM ± S.E.M.]	n[d]
**1**	(R,S)-cis	>20000	3	>20000	3	>50,000	3	>50,000	3	26,000 ± 2,100	3	11,000 ± 1,400	3	7,100 ± 2,600	3
**2**	(S,R)-cis	>20000	3	>20000	3	>50,000	3	>50,000	3	>50,000	3	>50,000	3	14,000 ± 2,600	3
**3**	(S,S)-trans	>20000	3	>20000	3	>50,000	3	>50,000	3	>50,000	3	>50,000	3	26,000 ± 1,500	3
**4**	(R,R)-trans	>20000	3	>20000	3	18,000 ± 4,000	3	19,000 ± 2,600	3	4,800 ± 1,800	3	9,100 ± 2,500	3	1,900 ± 320	3
**5**	(R,S)-cis	>20000	4	1,800 ± 660	4	>50,000	3	>50,000	3	29,000 ± 7,300	3	31,00 ± 4,600	3	>50,000	3
**6**	(S,R)-cis	>20000	3	3,000 ± 410	3	>50,000	3	>50,000	3	15,000 ± 1,100	3	9,700 ± 2,000	3	33,000 ± 10,000	3
**7**	(S,S)-trans	>20000	5	370± 44	6	>50,000	3	>50,000	3	15,000 ± 2,300	3	12,000 ± 1,900	3	44,000 ± 13,000	3
**8 (=c-ISO)**	(R,R)-trans	4,000 ± 1,300	14	27± 3.9	13	>50,000	3	>50,000	3	12,000 ± 2,400	3	20,000 ± 5,600	3	34,000 ± 7,500	3

Supplementary Table 1 | Receptor binding data for different isomers of the conformationally restricted N-isoprenaline (1-8) at adrenergic receptor subtypes β_1AR , β_2AR, α_{1A}AR, α_{1B}AR, α_{2A}AR, α_{2B}AR, and α_{2C}AR using membranes from HEK293T cells transfected with the appropriate receptor. Binding assay performed with a radioligand displacement assay using the [a] radioligand [^{3}H]CGP12,177, [b] radioligand [^{3}H]prazosin and [c] radioligand [^{3}H]RX821002. [d] Number of individual experiments each done in triplicates.

receptor	c-Epi			c-NorEpi			c-ISO			Epi			NorEpi			ISO		
	EC_{50} [nM][a]	E_{max} [%][b]	n[c]	EC_{50} [nM][a]	E_{max} [%][b]	n[c]	EC_{50} [nM][a]	E_{max} [%][b]	n[c]	EC_{50} [nM][a]	E_{max} [%][b]	n[c]	EC_{50} [nM][a]	E_{max} [%][b]	n[c]	EC_{50} [nM][a]	E_{max} [%][b]	n[c]
arrestin recruitment[d]																		
β_1AR_WT	15,000 ± 1,900	57 ± 6	15	12,000 ± 2,700	58 ± 4	13	1,500 ± 290	100 ± 4	14	360 ± 43	98 ± 2	6	150 ± 12	100	9	22 ± 5.5	106 ± 2	10
β_1AR_4 mut	1,200 ± 140	93 ± 2	7	780 ± 110	100 ± 3	5	60 ± 2.8	93 ± 2	5	-	-	-	1400 ± 90	100	7			
$\beta_1AR_{in}/\beta_2AR_{out}$	220 ± 23	98 ± 3[e]	12	3600 ± 860	106 ± 5[e]	8	250 ± 39	104 ± 3[e]	8	4600 ± 780	100[e]	15	28000 ± 15000	43 ± 4[e]	6	1600 ± 310	103 ± 2[e]	13
β_2AR_WT	24 ± 2.2	112 ± 2	21	130 ± 15	111 ± 2	13	19 ± 3.1	109 ± 1	21	180 ± 13	110 ± 1	17	8100 ± 580	100	9	67 ± 14	105 ± 2	12
β_2AR_4 mut	310 ± 28	93 ± 4	7	2,400 ± 460	86 ± 5	7	1,100 ± 370	101 ± 3	8	-	-	-	490 ± 38	100	9			
$\beta_2AR_{in}/\beta_1AR_{out}$	8300 ± 840	99 ± 7[e]	11	7400 ± 2700	87 ± 5[e]	10	580 ± 110	101 ± 3[e]	10	80 ± 18	100[e]	6	29 ± 3	103 ± 1[e]	8	5.3 ± 0.36	112 ± 3[e]	7
α_{1A}AR_WT	11,000 ± 7,600	12 ± 2	7	2,300 ± 1,500	18 ± 5	5	na	<5	8	-	-	-	620 ± 67	100	13	-	-	-
α_{1B}AR_WT	8,400 ± 3,000	11 ± 2	4	7,500 ± 760	19 ± 2	4	na	<5	4	-	-	-	83 ± 17	100	5	-	-	-
α_{2A}AR_WT	na	<5	8	na	<5	5	na	<5	8	-	-	-	620 ± 67	100	13	-	-	-
α_{2B}AR_WT	na	<5	4	na	<27 (100µM)[f]	4	na	<5	4	-	-	-	150 ± 27	100	11	-	-	-
α_{2C}AR_WT	na	<18 (100µM)[f]	4	20,000 ± 1,100	33 ± 2	4	na	<13 (100µM)[f]	4	-	-	-	110 ± 50	100	5	-	-	-
G-protein signaling[g]																		
β_1AR_WT + $G\alpha_{qs}$	5,900 ± 890	75 ± 3	4	-	-	-	-	-	-	240 ± 40	97 ± 1	6	110 ± 16	100	9	27 ± 5	99 ± 2	6
β_2AR_WT + $G\alpha_{qs}$	23 ± 4.8	109 ± 2	7	-	-	-	-	-	-	180 ± 54	108 ± 3	7	1900 ± 290	100	14	43 ± 10	107 ± 3	7

Supplementary Table 2 | Functional data for the restricted agonists c-Epi, c-NorEpi, c-ISO, and Epi and NorEpi in arrestin recruitment for the adrenergic receptors α_{1A}AR, α_{1B}AR, α_{2A}AR, α_{2B}AR, β_1AR and β_2AR, as well as the $\beta_1AR_{in}/\beta_2AR_{out}$ and $\beta_2AR_{in}/\beta_1AR_{out}$ chimeras and the β_1AR_4mut and β_2AR_4mut mutants. [a] Potency displayed as mean EC_{50} value in nM ± SEM. [b] Maximum effect in % ± SEM relative to the effect of the reference agonist norepinephrine. [c] Number of individual experiments each done in duplicates. [d] Determined with a fragment complementation based assay (PathHunter®) applying the appropriate receptor fused to the PK1 fragment or the ARMS2-PK2 fragment for enzyme complememtation in HEK cells stably expressing the enzyme acceptor (EA) tagged β-arrestin-2 fusion protein. [e] Maximum effect in % ± SEM relative to the effect of Epi. [f] No complete dose-response curve; maximum efficacy at the indicated concentration (in brackets). [g] Measurement of G-protein signaling by applying a homologous TR-FRET assay for IP detection (IP-One®) after co-transfection of the appropriate receptor and the hybrid G-protein $G\alpha_{qs}$ in HEK293T cells. na: No EC_{50} value could be calculated.

a

Cyclase		Epi	c-Epi	Fold c-Epi/ Epi	NorEpi	c-NorEpi	Fold c-NorEpi/ NorEpi	ISO	c-ISO	Fold c-ISO/ ISO
Log(EC_{50}), M	β_1AR	-8.2 ± 0.2	-7.1 ± 0.1	~14x***	-8.9 ± 0.2	-6.9 ± 0.2	~92x***	-8.4 ± 0.1	-7.9 ± 0.1	2.7x***
	β_2AR	-8.2 ± 0.2	-8.7 ± 0.1	~0.3x*	-7.0 ± 0.2	-8.8 ± 0.2	~0.02x***	-8.8 ± 0.1	-8.4 ± 0.1	NS
	Selectivity over β_1AR	NS	47x***		0.002x***	20x***		2.5x*	2.9x*	
Emax ($x10^{-3}$)	β_1AR	9.3 ± 0.5	8.3 ± 0.4	~0.9x*	9.9 ± 0.5	7.8 ± 0.3	~0.8x***	11 ± 0.4	11 ± 0.5	NS
	β_2AR	7.7 ± 0.4	8.0 ± 0.4	NS	8.3 ± 0.5	7.7 ± 0.7	NS	8.2 ± 0.3	9.7 ± 0.4	~1.2x**
	β_2AR/β_1AR	0.8x*	NS		0.8x***	NS		0.8x***	0.9x*	

b

Arrestin Recruitment		Epi	c-Epi	Fold c-Epi/ Epi	NorEpi	c-NorEpi	Fold c-NorEpi/ NorEpi	ISO	c-ISO	Fold c-ISO/ ISO
Log(EC_{50}), M	β_1AR	-6.2 ± 0.1	-4.9 ± 0.2	19x**	-6.5 ± 0.1	-5.1 ± 0.1	25x***	-7.8 ± 0.1	-5.7 ± 0.1	107x***
	β_2AR	-6.4 ± 0.1	-7.7 ± 0.1	0.05x***	-5.2 ± 0.1	-6.8 ± 0.1	0.03x***	-7.7 ± 0.1	-7.6 ± 0.1	NS
	Selectivity over β_1AR	1.4x*	594x***		0.06x***	52x*		NS	73x***	
Emax (AU)	β_1AR	101 ± 2	53 ± 3	0.5x**	102 ± 2	54 ± 2	0.53x***	93 ± 1	96 ± 2	NS
	β_2AR	112 ± 2	120 ± 3	1.1x*	102 ± 2	115 ± 2	1.1x**	108 ± 2	84 ± 3	0.8x***
	β_2AR/β_1AR	1.1x**	2.3x**		NS	2.1x***		0.9x***	1.1x**	

Supplementary Table 3 | Adenylyl cyclase activation and arrestin recruitment by non-constrained and constrained catecholamines . Summary of the dose-response relationship of catecholamine-stimulated cAMP accumulation (a) and β-arrestin recruitment assay (b) on β_1AR or β_2AR. Log(EC_{50})s and maximal activities were determined to a single site logistics curve using Prism (GraphPad, La Jolla, CA). Data are representative of n=3-6 individual experiments. Statistics represent paired comparisons of either non-constrained vs constrained catecholamines on either β_1AR or β_2AR (relative to non-constrained), or, β_1AR vs β_2AR stimulated by either non-constrained or constrained catecholamines (relative to β_2AR). P values were determined using the Extra Sum of Squares F test (GraphPad Prism 9, CA) and are indicated for the Log (EC_{50}) and Vmax values (*p<0.05, ** p<0.001, ***p<0.0001, NS not statistically different).

		Epi	c-Epi	Ki(c-Epi)/ Ki(Epi)	NorEpi	c-NorEpi	Ki(c-NorEpi)/ Ki(NorEpi)	ISO	c-ISO	Ki(c-ISO)/ Ki(ISO)
β_1AR	wt	-6.4 ± 0.1	-4.9 ± 0.1	33.7***	-6.7 ± 0.1	-5.0 ± 0.1	51.1***	-7.5 ± 0.1	-6.5 ± 0.1	9.8***
	4mut	-6.0 ± 0.1	-5.5 ± 0.1	2.9**	-5.6 ± 0.1	-4.8 ± 0.1	5.8***	-7.2 ± 0.1	-7.1 ± 0.1	1.3*
	Ki(4mut)/ Ki(wt)	2.8***	0.2***		12.7***	1.4**		2.0***	0.3**	
β_2AR	wt	-7.0 ± 0.1	-7.9 ± 0.1	0.1***	-5.8 ± 0.1	-6.2 ± 0.1	0.4***	-7.6 ± 0.1	-8.1 ± 0.1	0.3***
	4mut	-7.4 ± 0.1	-6.9 ± 0.1	3.2***	-6.5 ± 0.1	-5.5 ± 0.1	10.7***	-7.7 ± 0.1	-7.4 ± 0.1	2.1*
	Ki(4mut)/ Ki(wt)	0.3***	8.8***		0.2***	5.2***		0.8**	5.4***	
Fold selectivity of β_2AR-wt over β_1AR-wt		3.3*	891.3***		0.1***	15.6***		NS	43.9***	
Fold selectivity of β_2AR-4mut over β_1AR-4mut		26.8***	24.5***		8.0***	4.3***		3.5**	4.6***	

Supplementary Table 4 | Inhibition of [^{3}H]DHA binding by non-constrained and constrained catecholamines . Summary of the competition of [^{3}H]DHA by non-constrained and constrained catecholamines in membranes prepared from *Sf*9 cells infected with baculoviruses. Ki values were determined using the IC_{50} values determined by a single site logistics curve using Prism and adjusted for Kd values for [^{3}H]DHA for β_1AR and β_2AR (and mutants thereof) and the concentration used in the assay, according to Cheng-Prusoff equation (GraphPad, La Jolla, CA). Data are representative of n=3-6 individual experiments. Statistics represent paired comparisons of either non-constrained vs constrained catecholamines on either β_1AR, β_2AR (shaded) or comparisons with the corresponding vestibule mutants (4mut) (relative to non-constrained). Additional comparison of βAR(WT) vs βAR(4mut) of either β_1AR or β_2AR, by either non-constrained or constrained catecholamines are also included. P values were determined using the Extra Sum of Squares F test (GraphPad Prism 9, CA) and are indicated for the Log (K_i) (*p<0.05, ** p<0.001, *** p<0.0001, or NS not statistically different).

β_1AR	K_{on} ($M^{-1}min^{-1}$)	n	K_{off} (min^{-1})	n	K_{off}/K_{on} (nM)	n
NorEpi*	$2.0 \pm 0.1 \times 10^5$	7	$6.4 \pm 1.2 \times 10^{-2}$	7	$3.1 \pm 0.5 \times 10^2$	7
c-NorEpi	$1.3 \pm 0.3 \times 10^4$	5	$1.7 \pm 0.4 \times 10^{-1}$	5	$1.3 \pm 0.1 \times 10^4$	5
Epi*	$6.2 \pm 0.6 \times 10^4$	5	$5.1 \pm 0.9 \times 10^{-2}$	5	$8.2 \pm 1 \times 10^2$	5
c-Epi	$1.1 \pm 0.1 \times 10^4$	5	$1.3 \pm 0.4 \times 10^{-1}$	5	$1.1 \pm 0.2 \times 10^4$	5

β_2AR	K_{on} ($M^{-1}min^{-1}$)	n	K_{off} (min^{-1})	n	K_{off}/K_{on} (nM)	n
NorEpi*	$8.5 \pm 1.5 \times 10^3$	4	$4.1 \pm 1.2 \times 10^{-2}$	4	$4.7 \pm 0.6 \times 10^3$	4
c-NorEpi	$1.7 \pm 0.2 \times 10^4$	4	$1.9 \pm 0.1 \times 10^{-2}$	4	$1.2 \pm 0.2 \times 10^3$	4
Epi*	$1.2 \pm 0.2 \times 10^5$	7	$4.7 \pm 1.1 \times 10^{-2}$	7	$3.8 \pm 0.6 \times 10^2$	7
c-Epi	$3.6 \pm 0.8 \times 10^5$	8	$4.1 \pm 0.6 \times 10^{-2}$	8	$1.3 \pm 0.2 \times 10^2$	8

$\beta_1AR_{in}/\beta_2AR_{out}$	K_{on} ($M^{-1}min^{-1}$)	n	K_{off} (min^{-1})	n	K_{off}/K_{on} (nM)	n
NorEpi*	$6.7 \pm 0.8 \times 10^3$	3	$1.0 \pm 0.1 \times 10^{-1}$	3	$1.4 \pm 0.2 \times 10^4$	3
c-NorEpi	$1.9 \pm 0.1 \times 10^4$	3	$1.2 \pm 0.5 \times 10^{-1}$	3	$4.3 \pm 1.1 \times 10^3$	3
Epi*	$6.3 \pm 1.1 \times 10^4$	4	$2.5 \pm 0.4 \times 10^{-1}$	4	$4.1 \pm 0.5 \times 10^3$	4
c-Epi	$8.7 \pm 3.5 \times 10^4$	4	$1.7 \pm 0.6 \times 10^{-1}$	4	$2.2 \pm 0.5 \times 10^3$	4

$\beta_2AR_{in}/\beta_1AR_{out}$	K_{on} ($M^{-1}min^{-1}$)	n	K_{off} (min^{-1})	n	K_{off}/K_{on} (nM)	n
NorEpi*	$4.9 \pm 1.3 \times 10^5$	8	$9.4 \pm 2.1 \times 10^{-2}$	8	$2.5 \pm 0.5 \times 10^2$	8
c-NorEpi	$3.2 \pm 0.9 \times 10^4$	5	$2.2 \pm 0.7 \times 10^{-1}$	5	$7.1 \pm 1.1 \times 10^3$	5
Epi*	$1.3 \pm 0.3 \times 10^5$	4	$3.8 \pm 1.2 \times 10^{-2}$	4	$2.8 \pm 0.5 \times 10^2$	4
c-Epi	$1.3 \pm 0.4 \times 10^4$	4	$8.7 \pm 4.4 \times 10^{-2}$	4	$5.5 \pm 1.9 \times 10^3$	4

Supplementary Table 5 | The binding kinetics of non-constrained and constrained NorEpi or Epi to the β_1AR, β_2AR as well as the $\beta_1AR_{in}/\beta_2AR_{out}$ and $\beta_2AR_{in}/\beta_1AR_{out}$ chimeras. * The parameters of NorEpi and Epi are derived from a previous publication of our group[1].

a

Log(τ/Ka)		Epi	c-Epi	NorEpi	c-NorEpi	ISO	c-ISO
cAMP accumulation	β_1AR	8.4 ± 0.2	7.0 ± 0.2***	9.8 ± 0.6	6.4 ± 0.4***	8.3 ± 0.2	8.1 ± 0.2
	β_2AR	8.0 ± 0.2	8.6 ± 0.2*	6.5 ± 0.5	7.8 ± 0.4**	8.0 ± 0.2	8.2 ± 0.2
β-arrestin recruitment	β_1AR	6.2 ± 0.05	4.9 ± 0.01*	6.9 ± 0.05	5.2 ± 0.09***	7.8 ± 0.05	5.6 ± 0.08
	β_2AR	6.4 ± 0.02	7.7 ± 0.03***	5.0 ± 0.04	6.9 ± 0.02***	7.7 ± 0.05	7.6 ± 0.04

b

		c-Epi vs Epi		c-NorEpi vs NorEpi		c-ISO vs ISO	
		β_2AR	β_1AR	β_2AR	β_1AR	β_2AR	β_1AR
cAMP accumulation	ΔLog(τ/Ka)	0.6 ± 0.2	-1.4 ± 0.2	1.3 ± 0.6	-3.4 ± 0.7	0.2 ± 0.3	-0.7 ± 0.2
	Relative Effectiveness	3.84	0.04	20.23	0.00	1.56	0.57
β-arrestin recruitment	ΔLog(τ/Ka)	1.3 ± 0.03	-1.3 ± 0.1	1.8 ± 0.0	-1.7 ± 0.1	-0.06 ± 0.06	-2.1 ± 0.1
	Relative Effectiveness	20.84	0.05	65.01	0.02	0.86	0.01
	ΔΔLog(τ/Ka)	0.7 ± 0.2	0.1 ± 0.3	0.5 ± 0.6	1.7 ± 0.7	-0.3 ± 0.3	-1.9 ± 0.3
	Bias Factor	5.43	1.20	3.21	51.17	0.55	0.01

Supplementary Table 6 | Relative effectiveness and Bias of constrained catecholamines on β_1AR vs β_2AR on cAMP accumulation and β_2-arrestin recruitment. a. Log(τ/Ka) values for non- and constrained-catecholamines in cAMP and β-arrestin recruitment assays. (*p<0.05, ** p<0.001, or *** p<0.0001). P values were determined using the Extra Sum of Squares F test (GraphPad Prism 9, CA). **b**. Bias factors of constrained catecholamines in cAMP and β-arrestin recruitment assays. The ΔLog(τ/Ka), relative effectiveness and ΔΔLog(τ/Ka) values were determined from Log(τ/Ka) values for each ligand using the Operational Model for Bias[2] (using GraphPad Prism 9, CA) from cAMP accumulation and β-arrestin recruitment data described in Figure 2 and Supplementary Figure 1.

	T4L-β2AR/ Nb6B9/ cEpi	T4L-β2AR/ Nb6B9/ cISO
**Data collection**		
Space group	P 21 21 21	P 21 21 21
Cell dimensions		
a, *b*, *c* (Å)	49.6, 66.56, 302.72	49.6, 66.56, 302.72
a, b, g (°)	90, 90, 90	90, 90, 90
Resolution (Å)	50 – 3.1 (3.2 -3.1)	50 – 3.4 (3.5-3.4)
R_{sym} or R_{merge}	0.36 (1.37)	0.43 (1.30)
I / s*I*	6.73 (1.10)	6.76□ 1.18□
Completeness (%)	99.5 (97.2)	99.5 (97.4)
Redundancy	11.38 (5.32)	12.70□ 5.55□
**Refinement**		
Resolution (Å)	20-3.1	20-3.4
No. reflections (test set)	18885 (1858)	14444(1445)
R_{work} / R_{free}	0.23 /0.27	0.23/0.26
No. atoms		
Protein	4507	4507
Ligand	16	18
Others (Lipids, ions, water)	2	1
B-factors		
Receptor	58.77	55.99
T4L	64.64	64.93
Nb 6B9	68.68	66.05
Ligand	50.08	55.58
Others (Lipids, ions, water)	44.61	47.77
R.m.s. deviations		
Bond lengths (Å)	0.003	0.003
Bond angles (°)	0.54	0.52
Ramachandran favored (%)	97.18	97.18
Ramachandran allowed (%)	2.82	2.82
Ramachandran outliers (%)	0.00	0.00

*Values in parentheses are for highest-resolution shell.

Supplementary Table 7 | Data collection and refinement statistics.

Supplementary Notes

Chemical Synthesis (see also Supplementary Fig 16-19)

General: All chemicals and solvents were purchased from Sigma Aldrich, Acros Organics, Alfa Aesar, or Activate Scientific and were used without additional purification. (*R*)-(+)-α-methylbenzyl isocyanate was bought from TCI (*ee* > 98%) and used as soon as possible after delivery. Anhydrous solvents were of the highest commercially available grade and were stored over molecular sieves under a nitrogen atmosphere. Flash chromatography was performed on Merck silica gel 60 (40-63 µm) as stationary phase under positive pressure of dry nitrogen gas. Automated flash column chromatography was performed with a Biotage SP1 Flash Chromatography Purification System using either Biotage® SNAP KP-Sil or Biotage® SNAP KP-C18-HS columns (10 g or 50 g loading). Preparative RP-HPLC was performed on an Agilent 1100 Preparative Series, using a ZORBAX ECLIPSE XDB-C8 PrepHT (21.5 x 150 mm, 5 µm, flow rate 8-12 mL/min) column with the solvent systems indicated. Analytical RP-HPLC was conducted on an Agilent 1200 HPLC system employing a DAD detector and a ZORBAX ECLIPSE XDB-C8 (4.6 x 150 mm, 5 µm) column with the following binary solvent systems: System 1: eluent, acetonitrile/0.1% aq. TFA, 4% acetonitrile at 0 min, to 25% at 24 min, 40% at 26 min, 40% until 28 min, 95% at 30 min, 95% until 32 min, 4% at 34 min, 4% until 37 min. Flow rate 0.5 mL/min, λ = 254 or 280 nm; System 2: eluent, methanol/0.1% aq. TFA, 5% methanol at 0 min, to 30% at 24 min, 50% at 26 min, 50% until 28 min, 95% at 30 min, 95% until 32 min, 5% at 34 min, 5% until 37 min. Flow rate 0.5 mL/min, λ = 254 or 280 nm. Preparative chiral HPLC was performed on an Agilent 1100 HPLC systems employing a VWL detector using a Daicel semi-preparative CHIRALPAK® IC column (10 x 250 mm, 5 µm particle size, flow rate 5-7 mL/min). Analytical chiral HPLC was performed on an Agilent series 1200 system using a DAD and a Daicel CHIRALPAK® IC column (4.6 x 250 mm, 5 µm particle size) with CHIRALPAK® IC guard cartridge (4 x 10 mm, 5 µm particle size) with following isocratic conditions: *n*-hexane/isopropanol + 0.1% ethylenediamine 9:1, 0.7 mL/min flowrate, 20 °C column temperature (system Ch1). Optical

rotation was measured with a JASCO C-2000 polarimeter (cylindrical glass cuvette with a path of 100 mm and a volume of 1 mL). Melting points were determined using a MEL-TEMP II apparatus (Laboratory Devices, U.S.) and are uncorrected. TLC analyses were conducted using Merck 60 F254 aluminum plates in combination with UV detection (254 nm) or staining reagents. HR-MS was run on a AB Sciex Triple TOF660 SCiex, source type ESI, or on a Bruker maXis MS in the laboratory of the Chair of Organic Chemistry, FAU, or on a Bruker maXis MS in the laboratory of the Chair of Bioinorganic Chemistry, FAU. Mass detection was conducted with a Bruker Esquire 2000 ion trap mass spectrometer using APCI or ESI ionization source or with Bruker amaZon SL mass spectrometer in combination with a Agilent 1100 or Dionex Ultimate 3000 UHPLC system; respectively ^{1}H, and ^{13}C spectra were recorded on a Bruker Avance 360, Avance 400 or a Bruker Avance 600 FT-NMR spectrometer. Chemical shifts were calculated as ppm relative to TMS or solvent signal as internal standards.

6,7-Bis(benzyloxy)-3,4-dihydronaphthalen-1(2H)-one **(9)**

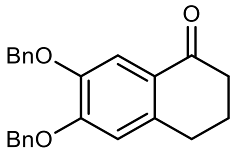

6,7-Dimethoxy-1-tetralone (2.18 g, 10.6 mmol) was dissolved in toluene (60 mL), and nitrogen was bubbled through the solution for 15 min. Anhydrous $AlCl_3$ (7.05 g, 52.9 mmol) was added, and the solution was heated to reflux. After 1 h, stirring was continued at r.t.. After 16 h, the reaction was cooled on ice, and water (20 mL) and 2 M HCl (20 mL) were added. After 15 min, the precipitate was collected by filtration and washed with water. The solid was dissolved in MeOH (30 mL) and filtered through a sintered filter. The filtrate was evaporated to dryness, and the remaining olid was dissolved in acetone (60 mL). To the solution was added benzyl bromide (3.02 mL, 25.4 mmol), potassium carbonate (5.86 g, 42.4 mmol) and sodium iodide (254 mg, 1.69 mmol), and the reaction was heated to reflux for 16 h. The solid was removed by filtration through sinter and washed with acetone (40 mL). The

filtrate was evaporated to dryness and the resulting product was purified by recrystallization from *n*-hexane/CH_2Cl_2 to give **9** as pale yellow needles (2.02 g, 53%).

^{1}H NMR (600 MHz, $CDCl_3$): δ 7.64 (s, 1H), 7.50 – 7.43 (m, 4H), 7.41 – 7.29 (m, 6H), 6.74 (s, 1H), 5.22 (s, 2H), 5.19 (s, 2H), 2.85 (t, *J* = 6.1 Hz, 2H), 2.61 – 2.55 (m, 2H), 2.14 – 2.06 (m, 2H); ^{13}C NMR (151 MHz, $CDCl_3$): δ 197.3, 153.5, 147.7, 139.7, 137.1, 136.6, 128.7, 128.6, 128.1, 128.0, 127.5, 127.2, 126.4, 113.0, 111.8, 71.1, 70.9, 38.7, 29.6, 23.7.

6,7-Bis(benzyloxy)-2,2-dibromo-3,4-dihydronaphthalen-1(2H)-one (**10**)

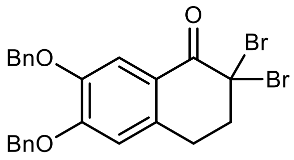

Compound **9** (2.00 g, 5.58 mmol) was dissolved in Et_2O (30 mL) and CH_2Cl_2 (30 mL). To the stirred solution was dropwise added a solution of bromine (572 µL, 11.2 mmol) in Et_2O (30 mL) at r.t. After 5 min, 50% $NaHCO_3$ solution (20 mL) was slowly added, and the product was extracted with CH_2Cl_2 (2 × 30 mL). The combined organic layers were washed with $Na_2S_2O_3$ (10% aq. solution, 30 mL), brine, dried (Na_2SO_4) and evaporated. The residue was purified by recrystallization (*n*-hexane/CH_2Cl_2) to give **10** as white needles (2.26 g, 93%).

^{1}H NMR (360 MHz, $CDCl_3$): δ 7.71 (s, 1H), 7.50 – 7.28 (m, 10H), 6.71 (s, 1H), 5.23 (s, 2H), 5.19 (s, 2H), 3.07 – 3.00 (m, 2H), 3.00 – 2.94 (m, 2H); ^{13}C NMR (91 MHz, $CDCl_3$): δ 183.4, 154.6, 148.6, 137.6, 136.7, 136.2, 128.8, 128.7, 128.3, 128.2, 127.5, 127.2, 120.5, 113.8, 112.4, 77.4, 71.2, 71.0, 67.5, 53.6, 46.5, 31.7, 29.3, 22.8, 14.3.

6,7-Bis(benzyloxy)-2-bromo-3,4-dihydronaphthalen-1(2H)-one (**11**)

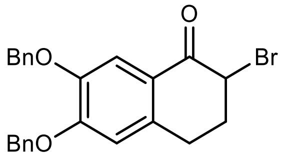

Compound **10** (2.70 g, 5.23 mmol) was dissolved in dry THF (20 mL) and cooled on ice. To the solution was dropwise added a solution of diethyl phosphite (743 µL, 5.75 mmol) and triethylamine (802 µL, 5.75 mmol) in THF (10 mL). After 3 d, water (30 mL) was added, and the product was extracted with EtOAc (3 × 40 mL). The combined organic layers were

washed with brine, dried (Na_2SO_4), evaporated, and the product was purified by recrystallization (*n*-hexane/CH_2Cl_2) to give **11** as pale orange needles (1.51 g, 66%).

^{1}H NMR (360 MHz, $CDCl_3$): δ 7.66 (s, 1H), 7.49 – 7.27 (m, 10H), 6.75 (s, 1H), 5.23 (s, 2H), 5.18 (s, 2H), 4.67 (t, *J* = 4.2 Hz, 1H), 3.21 (ddd, *J* = 16.4, 9.8, 4.9 Hz, 1H), 2.77 (dt, *J* = 16.9, 4.3 Hz, 1H), 2.53 – 2.36 (m, 2H); ^{13}C NMR (91 MHz, $CDCl_3$): δ 189.4, 154.1, 148.0, 138.2, 136.6, 136.2, 128.6, 128.5, 128.0, 127.9, 127.3, 127.0, 123.2, 112.4, 112.4, 70.9, 70.7, 50.2, 32.2, 25.8.

2-Azido-6,7-bis(benzyloxy)-3,4-dihydronaphthalen-1(2H)-one **(<u>12</u>)**

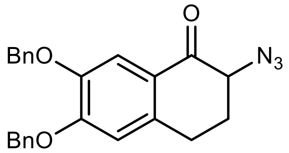

Compound **11** (1.78 g, 4.07 mmol) was dissolved in dry DMF (30 mL). Glacial acetic acid (279 µL, 4.88 mmol) was added, followed by sodium azide (529 mg, 8.14 mmol) in water (2 mL) at r.t. After 1 h, water (50 mL) was added, followed by CH_2Cl_2 (40 mL) and the product was extracted with further CH_2Cl_2 (2 × 30 mL). The combined organic layers were washed with brine, dried (Na_2SO_4) and concentrated in vacuo. The residue was dissolved in Et_2O (30 mL) and washed with water (3 × 50 mL), brine, dried ($MgSO_4$) and evaporated to dryness, to give **12** as a solid (1.46 g, 90%) which was used as a crude material for the next step.

^{1}H NMR (360 MHz, $CDCl_3$): δ 7.63 (s, 1H), 7.50 – 7.29 (m, 10H), 6.71 (s, 1H), 5.22 (s, 2H), 5.19 (s, 2H), 4.15 (dd, *J* = 11.7, 4.7 Hz, 1H), 2.93 (dd, *J* = 7.7, 4.7 Hz, 2H), 2.36 – 2.23 (m, 1H), 2.14 – 2.02 (m, 1H); ^{13}C NMR (91 MHz, $CDCl_3$) δ 192.6, 154.2, 148.1, 138.7, 136.8, 136.3, 128.8, 128.7, 128.3, 128.1, 127.5, 127.2, 124.6, 112.6, 112.0, 71.1, 70.9, 64.0, 29.7, 27.4.

2-Amino-6,7-bis(benzyloxy)-1,2,3,4-tetrahydronaphthalen-1-ol **(<u>13</u>)**

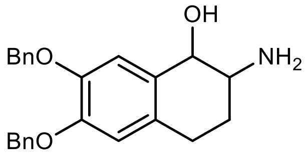

Compound **12** (440 mg, 1.10 mmol) was dissolved in 1,2-DCE (20 mL), and to the solution was dropwise added $LiAlH_4$ 1 M in THF (4.41 mL, 4.41 mmol, 4 eq.). After 16 h, water (5 mL) was added dropwise. The mixture was diluted with CH_2Cl_2 (20 mL), and extracted twice with CH_2Cl_2 (2 × 20 mL). The combined organic layers were washed with brine, dried (Na_2SO_4) and evaporated to give **13** required no further purification (325 mg, 76%, *cis*/*trans* approx. 1:1).

cis: ^{1}H NMR (600 MHz, $CDCl_3$): δ 7.47 – 7.43 (m, 4H), 7.39 – 7.33 (m, 4H), 7.33 – 7.29 (m, 2H), 7.04 (s, 1H), 6.68 (s, 1H), 5.19 – 5.11 (m, 4H), 4.44 (d, *J* = 3.7 Hz, 1H), 3.12 (m, 1H), 2.77 (dt, *J* = 16.8, 5.1 Hz, 1H), 2.70 (ddd, *J* = 16.5, 9.8, 6.0 Hz, 1H), 1.92 – 1.83 (m, 1H), 1.76 – 1.67 (m, 1H); *trans*: ^{1}H NMR (600 MHz, $CDCl_3$): δ 7.44 – 4.40 (m, 4H), 7.36 – 7.25 (m, 6H), 7.18 (s, 1H), 6.60 (s, 1H), 5.14 – 5.03 (m, 4H), 4.42 (d, *J* = 8.6 Hz, 1H), 2.97 (t, *J* = 8.7 Hz, 1H), 2.78 (ddd, *J* = 17.0, 11.6, 5.7 Hz, 1H), 2.70 – 2.61 (m, 1H), 2.06 – 1.99 (m, 1H), 1.77 – 1.64 (m, 1H); ESI-MS: *m/z* 376.2 $[M+H]^+$.

6,7-Bis(benzyloxy)-2-(isopropylamino)-1,2,3,4-tetrahydronaphthalen-1-ol **(14)**

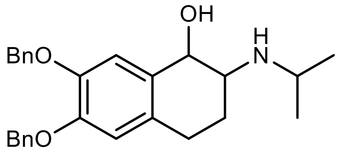

Compound **13** (80 mg, 213 µmol) was dissolved in 1,2-DCE (10 mL) in a dried flask, and to the solution was added acetone (1 mL), followed by $NaBH(OAc)_3$ (181 mg, 852 µmol). After 4 d, the reaction was diluted with CH_2Cl_2 (20 mL), washed with 1 M K_2CO_3 (2 × 20 mL) and brine, dried (Na_2SO_4) and evaporated to give a brown solid (85 mg, 96%). The crude compound was purified by preparative HPLC (Solvent A: 0.1% 0.1% TFA in H_2O, Solvent B: MeCN; gradient 5 – 80% Solvent B over 17 min, product eluted at 13.9 min) and lyophilized to give a white solid (120 mg, 89%). The mixture of siomers was further purified by semi-preparative chiral HPLC (MeCN + 0.1% ethylenediamine) resulting in elution of four fractions at t_R = 3.8 min (*trans*-1), 4.1 min (*cis*-2), 4.4 min (*cis*-1) and 4.8 min (*trans*-2). Assignment of the absolute configurations was done preliminarily based on HPLC comparison with the 5,6-dihydroxy-substituted analogs.

trans: ^{1}H NMR (600 MHz, $CDCl_3$): δ 7.49 – 7.40 (m, 4H), 7.39 – 7.27 (m, 6H), 7.19 (s, 1H), 6.64 (s, 1H), 5.18 – 5.09 (m, 4H), 4.46 (d, *J* = 8.8 Hz, 1H), 3.25 – 3.17 (m, 1H), 3.00 (br s, 1H), 2.88 – 2.73 (m, 3H), 2.23 – 2.18 (m, 1H), 1.68 – 1.58 (m, 1H), 1.21 (d, *J* = 6.3 Hz, 3H), 1.14 (d, *J* = 6.2 Hz, 3H); ^{13}C NMR (91 MHz, $CDCl_3$): δ 148.5, 148.1, 137.6, 137.5, 130.7, 128.6, 128.6, 128.1, 127.9, 127.8, 127.6, 127.4, 114.9, 113.2, 71.9, 71.5 (d), 58.9, 46.9, 27.9, 26.0, 23.0, 21.4; *cis*: ^{1}H NMR (600 MHz, $CDCl_3$) δ 7.49 – 7.41 (m, 4H), 7.39 – 7.34 (m, 4H), 7.32 – 7.28 (m, 2H), 7.07 (s, 1H), 6.68 (s, 1H), 5.20 – 5.10 (m, 4H), 4.50 (d, *J* = 4.0 Hz, 1H), 3.06 – 2.99 (m, 2H), 2.80 – 2.69 (m, 2H), 1.97 – 1.84 (m, 1H), 1.70 – 1.62 (m, 1H), 1.13 (d, *J* = 6.3 Hz, 3H), 1.12 (d, *J* = 6.3 Hz, 3H); ^{13}C NMR (91 MHz, $CDCl_3$): δ 148.9, 147.9, 137.6, 137.5, 130.1, 129.2, 128.6, 128.6, 127.8, 127.8, 127.5, 127.4, 116.6, 115.0, 71.5, 71.5, 67.0, 54.5, 46.0, 27.4, 24.5, 23.8, 23.5; ESI-MS: *m/z* 418.3 $[M+H]^+$.

(1R,2S)-2-(isopropylamino)-1,2,3,4-tetrahydronaphthalene-1,6,7-triol trifluoroacetate **(1)**

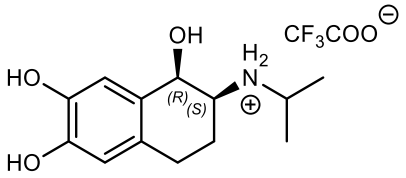

Compound **14** (*cis*-1, 7.52 mg, 0.018 mmol) was dissolved in methanol (8 mL). To this solution, 10% Pd/C (3.00 mg) was added and the formed suspension stirred under hydrogen atmosphere for 16 h. After filtration, and the filtrate was evaporated. The residue was purified by prep. HPLC (5-15% MeCN in water + 0.1% TFA over 15 min), to give **14** as a yellow oil (0.95 mg, 15% yield).

^{1}H NMR (600 MHz, CD_3OD): δ 6.79 (s, 1H), 6.56 (s, 1H), 4.69 (d, *J* = 2.6 Hz, 1H), 3.66 (sept, *J* = 6.5 Hz, 1H), 3.50 (dt, *J* = 12.6, 3.2 Hz, 1H), 2.87 – 2.75 (m, 2H), 2.12 (qd, *J* = 12.3, 6.3 Hz, 1H), 1.96 – 1.89 (m, 1H), 1.39 (d, *J* = 6.5 Hz, 3H), 1.37 (d, *J* = 6.5 Hz, 3H); ESI-HRMS: (*m/z*) $[MH]^+$ calcd. for $C_{13}H_{20}NO_3$, 238.1438; found, 238.1439.

(1S,2R)-2-(isopropylamino)-1,2,3,4-tetrahydronaphthalene-1,6,7-triol trifluoroacetate **(2)**

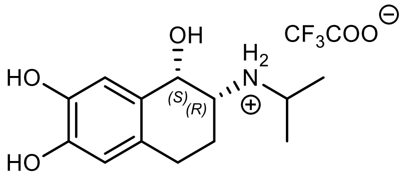

Compound **14** (*cis*-2, 12.0 mg, 0.029 mmol) was dissolved in methanol (8 mL). 10% Pd/C (3.00 mg) was added and the suspension was stirred under hydrogen atmosphere for 2 h. The reaction mixture was filtered, and the filtrate was evaporated. Pure product was obtained after purification by prep. HPLC (5-15% MeCN in water + 0.1% TFA over 15 min) as a white, amorphous solid (0.95 mg, 9% yield). For analytical data, see compound **1**.

(1S,2S)-2-(isopropylamino)-1,2,3,4-tetrahydronaphthalene-1,6,7-triol trifluoroacetate **(3)**

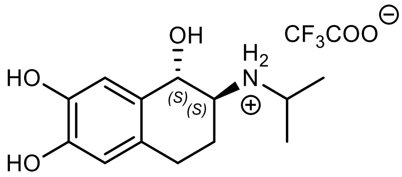

Compound **14** (*trans*-1, 5.00 mg, 0.012 mmol) was dissolved in methanol (4 mL). To the solution, 10% Pd/C (1.30 mg) was added and the formed suspension was stirred under hydrogen atmosphere for 2 h. The mixture was filtered, and the filtrate was evaporated. The pure product (**3**) was obtained after purification by prep. HPLC (5-15% MeCN in water + 0.1% TFA over 15 min) as a colorless oil (1.06 mg, 25% yield).

^{1}H NMR (600 MHz, CD_3OD): δ 6.96 (s, 1H), 6.53 (s, 1H), 4.57 (d, *J* = 8.7 Hz, 1H), 3.71 (sept, *J* = 6.5 Hz, 1H), 3.26 (ddd, *J* = 12.1, 8.9, 3.2 Hz, 1H), 2.86 (ddd, *J* = 16.8, 11.8, 5.2 Hz, 1H), 2.77 (ddd, *J* = 16.6, 5.1, 3.2 Hz, 1H), 2.32 – 2.23 (m, 1H), 1.82 (qd, *J* = 12.2, 5.5 Hz, 1H), 1.42 (d, *J* = 6.5 Hz, 3H), 1.37 (d, *J* = 6.5 Hz, 3H); ESI-HRMS: (*m*/*z*) $[MH]^+$ calcd. for $C_{13}H_{20}NO_3$, 238.1438; found, 238.1439.

(1R,2R)-2-(isopropylamino)-1,2,3,4-tetrahydronaphthalene-1,6,7-triol trifluoroacetate **(4)**

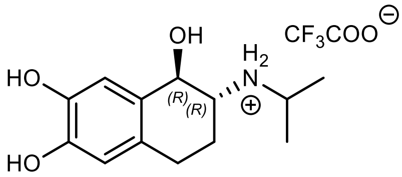

Compound **14** (*trans*-2) was deprotected as described for **3**. The mixture was filtered, and the filtrate was evaporated. The pure product (**4**) was obtained after purification by prep. HPLC (5-15% MeCN in water + 0.1% TFA over 15 min) as a clear oil. NMR data were identical to those observes for compound **3**.

(2-Carboxyethyl)triphenylphosphonium bromide **(15)**

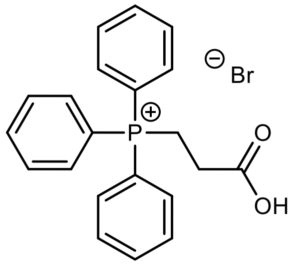

3-Bromopropionic acid (14.5 g, 94.8 mmol) was dissolved in acetonitrile (200 mL), and to the solution was added triphenylphosphine (27.4 g, 104 mmol). The solution was heated to reflux for 12 h. It was cooled to -5 °C on an ice/salt bath, and Et_2O (200 mL) was added which caused a precipitate to emerge. After stirring for 1 h, the solid was collected by filtration and washed with Et_2O (50 mL), to give pure **15** (33.2 g, 84%).

^{1}H NMR (600 MHz, DMSO-d_6): δ 12.73 (br s, 1H), 7.94 – 7.88 (m, 3H), 7.87 – 7.81 (m, 6H), 7.81 – 7.73 (m, 6H), 3.87 – 3.78 (m, 2H), 2.62 – 2.55 (m, 2H); ^{13}C NMR (151 MHz, DMSO-d_6): δ 171.5, 171.4, 134.98, 134.96, 133.7, 133.6, 130.3, 130.2, 118.3, 117.8, 26.78, 26.77, 17.0, 16.7.

4-(2,3-Dimethoxyphenyl)but-3-enoic acid **(16)**

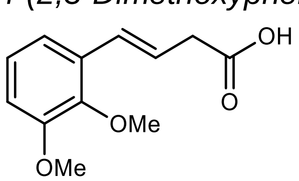

Sodium hydride (60% dispersion, 823 mg, 20.6 mmol) was added to a dried 2-neck flask, and with constant N_2 flow, was washed with hexane (2 × 5 mL). The remaining solid was dissolved in dry THF (15 mL) and dry DMSO (15 mL), To the mixture was slowly added compound **15** (4.27 g, 10.3 mmol), which caused foaming. After 5 min, 2,3-dimethoxybenzaldehyde (1.14 g, 6.86 mmol) was slowly added, and the mixture was allowed to stir under N_2 atmosphere

at r.t. After 2 h, the mixture was cooled on ice, and water was slowly added (40 mL. The precipitation was extracted with CH_2Cl_2 (3 × 20 mL), and the remaining aqueous layer was acidified with conc. HCl. The product was extracted with Et_2O (3 × 20 mL), and the combined organic layers were washed with brine, dried (Na_2SO_4) and evaporated to give a yellow oil. The product was purified by automated chromatography (gradient, *n*-hexane to 3:1 *n*-hexane/EtOAc) to give **16** as a white solid (930 mg, 61%).

^{1}H NMR (360 MHz, $CDCl_3$): δ 7.26 (s, 1H), 7.10 (dd, *J* = 7.9, 1.4 Hz, 1H), 7.00 (dd, *J* = 10.3, 5.7 Hz, 1H), 6.87 – 6.79 (m, 2H), 6.31 (dt, *J* = 16.0, 7.2 Hz, 1H), 3.86 (s, 3H), 3.81 (s, 3H), 3.33 (dd, *J* = 7.2, 1.4 Hz, 2H); ^{13}C NMR (151 MHz, $CDCl_3$): δ 177.7, 153.1, 146.7, 131.0, 128.3, 124.2, 122.4, 118.3, 111.6, 61.1, 55.9, 38.5; ESI-MS: *m/z* 245.0 $[M+Na]^+$.

4-(2,3-Dimethoxyphenyl)butanoic acid **(17)**

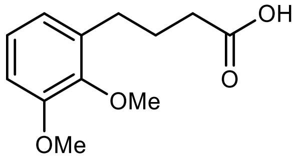

Compound **16** (4.80 g, 21.6 mmol) was dissolved in MeOH (80 mL) and added to a flask containing 10% Pd/C (2.30 g) in MeOH (10 mL). The mixture was stirred at r.t. under H_2 atmosphere. After 2 h, the mixture was filtered through celite, washed with MeOH (50 mL), and the filtrate was concentrated in vacuo to give **17** as a white solid (4.54 g, 94%).

^{1}H NMR (600 MHz, $CDCl_3$): δ 11.29 (br s, 1H), 6.99 (t, *J* = 7.9 Hz, 1H), 6.81 – 6.77 (m, 2H), 3.87 (s, 3H), 3.83 (s, 3H), 2.74 – 2.68 (m, 2H), 2.40 (t, *J* = 7.5 Hz, 2H), 2.00 – 1.90 (m, 2H); ^{13}C NMR (151 MHz, $CDCl_3$): δ 180.0, 152.9, 147.3, 135.2, 124.0, 122.1, 110.6, 60.7, 55.8, 33.6, 29.2, 25.6; ESI-MS: *m/z* 247.0 $[M+Na]^+$.

5,6-Dimethoxy-3,4-dihydronaphthalen-1(2H)-one **(18)**

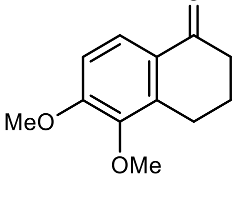

A 250 mL flask was charged with polyphosphoric acid (100 mL) and heated to 70 °C with mechanical stirring. Portions of compound **17** were added (4.62 g, 20.6 mmol) over 15 min.

After 1 h, the reaction was allowed to cool to r.t.. After 12 h, water (400 mL) was added and the mixture was extracted using EtOAc (3 × 100 mL). The combined organic layers were washed with brine, dried (Na_2SO_4) and evaporated to give **18** as a yellow solid (4.21 g, 99%).
^{1}H NMR (360 MHz, $CDCl_3$): δ 7.85 (d, *J* = 8.7 Hz, 1H), 6.87 (d, *J* = 8.7 Hz, 1H), 3.92 (s, 3H), 3.81 (s, 3H), 2.95 (t, *J* = 6.1 Hz, 2H), 2.58 (dd, *J* = 7.2, 5.9 Hz, 2H), 2.15 – 2.04 (m, 2H); ^{13}C NMR (151 MHz, $CDCl_3$): δ 197.6, 156.9, 145.5, 138.8, 126.8, 124.6, 110.2, 60.4, 55.9, 38.8, 23.4, 23.0; ESI-MS: *m/z* 207.0 $[M+H]^+$.

5,6-Dihydroxy-3,4-dihydronaphthalen-1(2H)-one **(19)**

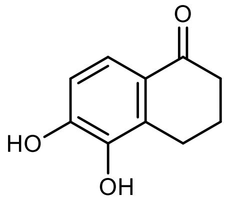

Compound **18** (1.90 g, 9.21 mmol) was dissolved in dry toluene (100 mL) which was degassed with N_2 for 15 min. To the solution was added $AlCl_3$ (6.14 g, 46.1 mmol). The mixture was heated to reflux for 1 h and subsequently cooled on ice. Then, water (30 mL) and 2 M HCl (30 mL) were sequentially added. The precipitate was collected by filtration and washed with water (30 mL). The solid was dried under vacuum to give pure **19** as a pale brown solid (1.15 g, 70%).
^{1}H NMR (600 MHz, DMSO-d_6): δ 10.14 (s, 1H), 8.57 (s, 1H), 7.31 (d, *J* = 8.4 Hz, 1H), 6.75 (d, *J* = 8.4 Hz, 1H), 2.78 (t, *J* = 6.1 Hz, 2H), 2.47 – 2.41 (m, 2H), 2.00 – 1.90 (m, 2H); ^{13}C NMR (151 MHz, $CDCl_3$): δ 196.5, 149.9, 141.4, 132.3, 125.0, 113.2, 94.8, 38.2, 22.9, 22.5; ESI-MS: *m/z* 179.0 $[M+H]^+$.

5,6-Bis(benzyloxy)-3,4-dihydronaphthalen-1(2H)-one **(20)**

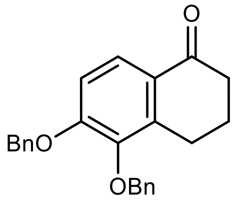

Benzyl bromide (2.30 mL, 19.4 mmol) was dissolved in acetone (80 mL) and NaI (2.13 g, 14.2 mmol) was added. After stirring at r.t. for 15 min, K_2CO_3 was added (4.46 g, 32.3 mmol),

followed by addition of **19** (1.15 g, 6.45 mmol). The mixture was heated to reflux for 2 h. Water (100 mL) was added, the product was extracted with EtOAc (3 × 50 mL) and the combined layers were washed with brine, dried (Na_2SO_4) and evaporated. The residue was purified by recrystallization from methanol (40 mL), and residual mother liquor was purified by flash column chromatography (4:1 *n*-hexane/EtOAc) to give **20** as a solid (2.09 g, 90%).

^{1}H NMR (600 MHz, $CDCl_3$): δ 7.86 (d, *J* = 8.7 Hz, 1H), 7.47 (d, *J* = 7.2 Hz, 2H), 7.43 – 7.32 (m, 8H), 6.99 (d, *J* = 8.7 Hz, 1H), 5.22 (s, 2H), 5.03 (s, 2H), 2.88 (t, *J* = 6.1 Hz, 2H), 2.60 – 2.52 (m, 2H), 2.07 – 1.98 (m, 2H); ^{13}C NMR (91 MHz, $CDCl_3$) δ 197.6, 156.1, 144.5, 139.4, 137.5, 136.4, 128.8, 128.6, 128.5, 128.4, 128.3, 127.6, 127.1, 124.6, 111.7, 74.7, 70.8, 38.8, 23.8, 23.0; ESI-MS: *m/z* 359.2 $[M+H]^+$.

5,6-Bis(benzyloxy)-2-bromo-3,4-dihydronaphthalen-1(2H)-one **(<u>21</u>)**

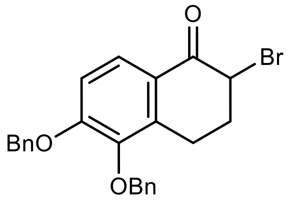

Compound **20** (410 mg, 1.14 mmol) was dissolved in Et_2O (20 mL) and a solution of bromine (117 µL, 2.29 mmol) in Et_2O (10 mL) was added to the stirred solution. After 1 h, 50% $NaHCO_3$ solution (20 mL) was slowly added, and the product was extracted with further Et_2O (2 × 20 mL). The combined organic layers were washed with $Na_2S_2O_3$ (10% aq. solution, 30 mL), brine, dried with Na_2SO_4 and concentrated in vacuo to give a mixture of the mono- and α,α-dibromo compounds. The crude product was dissolved in dry THF (10 mL) and cooled on ice. To this solution was dropwise added a solution of triethyl amine (167 µL, 1.20 mmol) and diethyl phosphite (154 µL, 1.20 mmol) in THF (10 mL) over a period of 10 min. After stirring for 16 h, water (20 mL) was added, and the product was extracted with EtOAc (2 × 20 mL). The combined organic layers were washed with brine, dried (Na_2SO_4), concentrated, and the residue was purified by flash column chromatography (5:1 *n*-hexane/EtOAc) to give **21** as a yellow oil (485 mg, 97%).

^{1}H NMR (360 MHz, $CDCl_3$): δ 7.91 (d, *J* = 8.8 Hz, 1H), 7.50 – 7.29 (m, 10H), 7.03 (d, *J* = 8.8 Hz, 1H), 5.23 (s, 2H), 5.06 (s, 2H), 4.64 (t, *J* = 4.2 Hz, 1H), 3.07 – 2.85 (m, 2H), 2.42 – 2.28

(m, 2H); ^{13}C NMR (91 MHz, $CDCl_3$) δ 189.8, 156.7, 144.4, 138.1, 137.3, 136.1, 128.9, 128.6, 128.6, 128.5, 128.4, 127.6, 126.2, 124.0, 112.3, 74.7, 70.9, 50.4, 31.6, 20.7; ESI-MS: *m/z* 437.1 $[M+H]^+$.

2-Azido-5,6-bis(benzyloxy)-3,4-dihydronaphthalen-1(2H)-one **(22)**

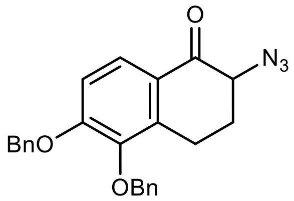

Compound **21** (1.44 g, 3.29 mmol) was dissolved in DMF (50 mL) and cooled on ice. To the stirred solution was added glacial acetic acid (226 µL, 3.95 mmol), then after 5 min, a solution of sodium azide (428 mg, 6.59 mmol) in water (3 mL). After 3 h stirring at 0 °C, water (50 mL) was added, followed by CH_2Cl_2 (40 mL), and the product was extracted with further CH_2Cl_2 (2 × 30 mL). The combined organic layers were washed with brine, dried ($MgSO_4$) and concentrated in vacuo. The oil was then dissolved in Et_2O (30 mL) and the solution was washed with water (3 × 50 mL), brine, dried (Na_2SO_4) and evaporated to crude **22** (1.22 g, 93%), which was could be immediately used for the next reaction step.

^1H NMR (360 MHz, $CDCl_3$): δ 7.88 (d, *J* = 8.7 Hz, 1H), 7.49 – 7.29 (m, 10H), 7.02 (d, *J* = 8.8 Hz, 1H), 5.23 (s, 2H), 5.07 – 4.97 (m, 2H), 4.12 (dd, *J* = 12.1, 4.7 Hz, 1H), 3.14 (dt, *J* = 17.6, 4.4 Hz, 1H), 2.75 – 2.62 (m, 1H), 2.25 (dq, *J* = 13.4, 4.5 Hz, 1H), 2.04 – 1.85 (m, 1H); ^{13}C NMR (91 MHz, $CDCl_3$): δ 192.8, 156.7, 144.5, 138.3, 137.3, 136.1, 128.9, 128.6, 128.6, 128.5, 128.4, 127.6, 125.4, 125.3, 112.4, 74.8, 71.0, 64.1, 28.9, 22.1; ESI-MS: *m/z* 400.2 $[M+H]^+$.

2-Amino-5,6-bis(benzyloxy)-1,2,3,4-tetrahydronaphthalen-1-ol **(23)**

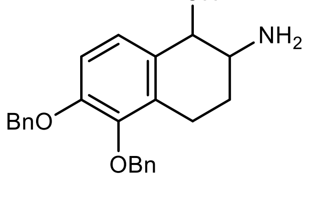

Compound **22** (550 mg, 1.38 mmol) was dissolved in 1,2-DCE (20 mL) and $LiAlH_4$ (1 M solution in THF, 4.13 mL, 4.13 mmol) was added over a period of 1 h. After 4 h, the reaction

was cooled on ice and quenched with water (30 mL). The mixture was further diluted with CH_2Cl_2 (50 mL), then filtered to remove solids. The product was further extracted with CH_2Cl_2 (3 × 30 mL), and the combined organic layers were washed with brine, dried (Na_2SO_4) and concentrated to give **23** as a yellow oil (485 mg, 94%), in approximately 2:3 *cis*/*trans* ratio.
^1H NMR (600 MHz, CD_3OD): δ 7.51 – 7.45 (m, 4H), 7.40 – 7.27 (m, 16H), 7.24 (dd, *J* = 8.5, 0.9 Hz, 1H), 7.11 (d, *J* = 8.5 Hz, 1H), 7.07 – 7.01 (m, 2H), 5.16 (m, 4H), 5.02 – 4.95 (m, 4H), 4.55 (d, *J* = 3.4 Hz, 1H), 4.30 (d, *J* = 8.2 Hz, 1H), 3.06 – 2.97 (m, 2H), 2.93 – 2.83 (m, 2H), 2.69 – 2.53 (m, 2H), 2.04 – 1.99 (m, 1H), 1.90 – 1.83 (m, 1H), 1.83 – 1.76 (m, 1H), 1.65 – 1.56 (m, 1H); ESI-MS: m/z 376.1 $[M+H]^+$.

5,6-Bis(benzyloxy)-2-(isopropylamino)-1,2,3,4-tetrahydronaphthalen-1-ol **(<u>24</u>)**

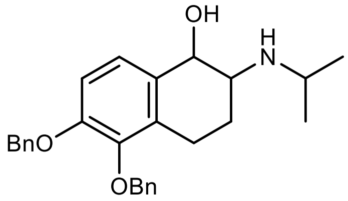

To a solution of **23** (100 mg, 266 µmol) in 1,2-DCE (10 mL), acetone (1 mL) and $NaBH(OAc)_3$ (226 mg, 1.07 mmol) were added. After 16 h, water (20 mL) was added, and the product was extracted with CH_2Cl_2 (3 × 20 mL). The combined organic layers were washed with brine, dried (Na_2SO_4) and evaporated. The isomers were then isolated by semi-preparative chiral HPLC (80:20 *n*-hexane/*i*-PrOH + 0.1% EDA). Three peaks eluted at t_R = 8.7 min for (*S*,*S*)-enantiomer (*trans*-1), 13.2 min for mixture of (*R*,*R*)- and (*R*,*S*)-enantiomers (*trans*-2, *cis*-1) and 16.5 min for (*S*,*R*)-enantiomer (*cis*-2), respectively.
trans: ^1H NMR (600 MHz, $CDCl_3$): δ 7.46 (dd, *J* = 7.9, 0.8 Hz, 2H), 7.43 – 7.35 (m, 4H), 7.35 – 7.30 (m, 4H), 7.29 (d, *J* = 8.6 Hz, 1H), 6.94 (d, *J* = 8.6 Hz, 1H), 5.14 (s, 2H), 5.04 – 4.99 (m, 2H), 4.37 (d, *J* = 8.7 Hz, 1H), 3.09 (sept, *J* = 6.5 Hz, 1H), 2.97 (ddd, *J* = 17.7, 5.7, 3.2 Hz, 1H), 2.74 – 2.65 (m, 2H), 2.40 (br s, 3H), 2.17 (ddt, *J* = 12.7, 6.2, 3.1 Hz, 1H), 1.48 (dtd, *J* = 12.9, 11.2, 5.9 Hz, 1H), 1.16 (d, *J* = 6.3 Hz, 3H), 1.12 – 1.06 (m, 3H); ^{13}C NMR (91 MHz, $CDCl_3$): δ 150.8, 145.5, 138.1, 137.3, 132.0, 130.7, 128.7, 128.5, 128.4, 128.0, 128.0, 127.6, 122.8, 112.9, 74.3, 72.7, 71.1, 58.1, 45.9, 26.1, 25.5, 24.3, 23.3, 22.5; *cis*: ^1H NMR (600 MHz,

CDCl3): δ 7.46 (d, *J* = 7.2 Hz, 2H), 7.44 – 7.29 (m, 8H), 7.19 (d, *J* = 8.4 Hz, 1H), 6.95 (d, *J* = 8.4 Hz, 1H), 5.15 (s, 2H), 5.05 (d, *J* = 10.9 Hz, 1H), 5.01 (d, *J* = 10.9 Hz, 1H), 4.56 (d, *J* = 3.8 Hz, 1H), 3.05 – 2.92 (m, 3H), 2.64 (ddd, *J* = 17.2, 10.4, 6.1 Hz, 1H), 1.87 (dtd, *J* = 12.7, 10.5, 5.7 Hz, 1H), 1.68 – 1.62 (m, 1H), 1.12 (t, *J* = 6.7 Hz, 6H); ^{13}C NMR (91 MHz, CDCl3): δ 151.2, 145.6, 138.1, 137.3, 131.1, 131.0, 128.7, 128.5, 128.4, 128.0, 128.0, 127.6, 126.3, 113.0, 74.3, 71.1, 67.1, 54.3, 46.0, 24.0, 23.9, 23.5, 22.6; ESI-MS: *m/z* 418.3 $[M+H]^+$.

(1S,2R)-6-(Isopropylamino)-5,6,7,8-tetrahydronaphthalene-1,2,5-triol trifluoroacetate **(6)**

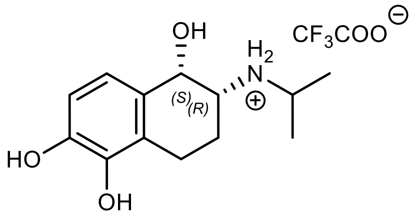

Compound **24** (*cis*-2, 15 mg, 36 µmol) was dissolved in methanol (4 mL). To this solution 10% Pd/C (1.5 mg) was added and the formed suspension was stirred under hydrogen atmosphere for 1 h. The mixture was filtered, and the filtrate was evaporated. The pure product was obtained after purification by prep. HPLC (5-15% MeCN in water + 0.1% TFA over 15 min) as a white, amorphous solid (4.60 mg, 54% yield). The absolute configuration was determined by derivatization of the benzyl-protected precursor with Mosher's reagent (*O*-acylation) and subsequent ^{1}H NMR studies[10].

(*1S,2S)-6-(Isopropylamino)-5,6,7,8-tetrahydronaphthalene-1,2,5-triol trifluoroacetate* **(7)**

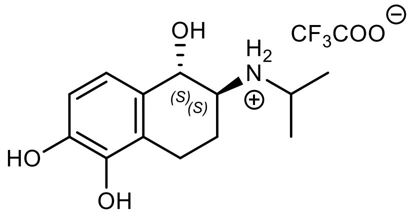

Compound **24** (*trans-1*, 12 mg, 28.7 µmol) was dissolved in methanol (4 mL). To this solution 10% Pd/C (1.2 mg) was added and the formed suspension stirred under hydrogen atmosphere for 1 h. The mixture was filtered, and the filtrate was evaporated. Pure **7** was

obtained after purification by prep. HPLC (5-15% MeCN in water + 0.1% TFA over 15 min) as a white, amorphous solid (3.20 mg, 47% yield).

^{1}H NMR (600 MHz, CD_3OD): δ 6.91 (dd, *J* = 8.4, 0.7 Hz, 1H), 6.74 (d, *J* = 8.3 Hz, 1H), 4.61 (d, *J* = 9.0 Hz, 1H), 3.72 (sept, *J* = 6.6 Hz, 1H), 3.27 (ddd, *J* = 12.1, 9.0, 3.2 Hz, 1H), 3.02 (ddd, *J* = 17.6, 5.6, 3.0 Hz, 1H), 2.73 (ddd, *J* = 17.5, 11.6, 5.8 Hz, 1H), 2.35 (ddt, *J* = 12.4, 6.0, 3.1 Hz, 1H), 1.81 (qd, *J* = 12.0, 5.7 Hz, 1H), 1.43 (d, *J* = 6.6 Hz, 3H), 1.38 (d, *J* = 6.6 Hz, 3H); ^{13}C NMR (150 MHz, CD_3OD): δ 143.2, 129.9, 123.7, 118.9, 114.6, 71.3, 60.2, 50.1, 24.6, 23.0, 19.9, 19.0; ESI-HRMS (*m/z*): $[MH]^+$ calcd. for $C_{13}H_{19}NO_3$, 238.1438; found, 238.1435.

(1R,2R)-6-(Isopropylamino)-5,6,7,8-tetrahydronaphthalene-1,2,5-triol trifluoroacetate (**8**) *and (1R,2S)-6-(isopropylamino)-5,6,7,8-tetrahydronaphthalene-1,2,5-triol trifluoroacetate* (**5**)

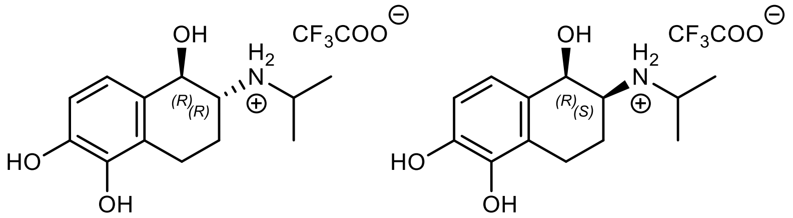

Compound **24** (mixture of *trans*-2 and *cis*-1, 77 mg, 0.18 mmol) was dissolved in ethanol (10 mL). To this solution was added 10% Pd/C (8.00 mg) and the formed suspension stirred under hydrogen atmosphere for 90 min. The mixture was filtered, and the filtrate was evaporated. The products were obtained after preparative HPLC (5-15% MeCN in water + 0.1% TFA over 15 min, two peaks, *trans* eluted at t_R = 9.1 min, *cis* eluted at t_R = 10.1 min) as white solids (37.0 mg, 61% for **8**, 8.00 mg, 13% for **5**).

5: ^{1}H NMR (600 MHz, CD_3OD): δ 6.76 (d, *J* = 8.3 Hz, 1H), 6.73 (d, *J* = 8.2 Hz, 1H), 4.76 (dd, *J* = 3.2, 1.0 Hz, 1H), 3.67 (sept, *J* = 6.5 Hz, 1H), 3.50 (dt, *J* = 12.7, 3.3 Hz, 1H), 3.09 (ddd, *J* = 17.6, 5.8, 1.8 Hz, 1H), 2.68 – 2.60 (m, 1H), 2.12 (qd, *J* = 12.5, 5.9 Hz, 1H), 2.04 – 1.96 (m, 1H), 1.40 (d, *J* = 6.5 Hz, 3H), 1.38 (d, *J* = 6.5 Hz, 3H); ^{13}C NMR (150 MHz, CD_3OD): δ 146.0, 128.5, 124.0, 122.7, 114.6, 65.6, 56.7, 48.3, 23.2, 21.2, 20.1, 19.0; ESI-HRMS (*m/z*): $[MH]^+$ calcd. for $C_{13}H_{19}NO_3$, 238.1438; found, 238.1435; **8**: see, compound **7**.

tert-butyl-((1RS,2RS)-5,6-bis(benzyloxy)-1-hydroxy-1,2,3,4-tetrahydronaphthalen-2-yl) carbamate **(25)**

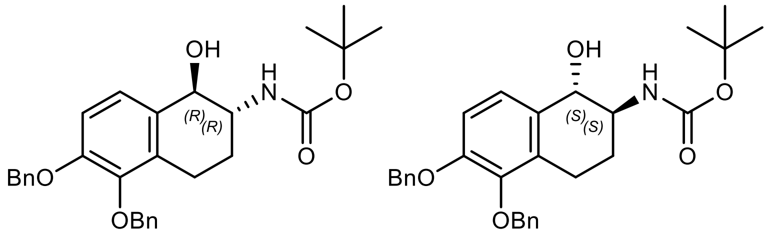

Compound **23** *cis*/*trans*-mixture (4.00 g, 10.6 mmol, approx. 70% *trans*) was dissolved in anhydrous CH_2Cl_2 (100 mL). After addition of *N*,*N*-diisopropylethylamine (3.62 mL, 21.3 mmol), Boc_2O (4.65 g, 21.3 mmol) was added under a stream of nitrogen and the reaction mixture was thereafter stirred overnight (18 h). It was evaporated and the residue was purified by flash column chromatography (isohexane/acetone 5:1 to 2:1), yielding a product enriched with the *trans*-isomers (>90%). After recrystallization of the beige-pink solid (toluene/ isohexane 2:1), a white, diastereomerically pure powder was obtained (3.01 g, 60% yield). Small amounts of *trans*-compound can be separated on chiral, preparative HPLC (ChiralPak IC) with acetonitrile as eluent, giving first (*R*,*R*)- and second (*S*,*S*)-enantiomer.

mp: 164–168 °C; TLC: R_f = 0.41 for *cis*, 0.35 for trans (*iso*-hexane:acetone 2:1 v/v); $[\alpha]_D^{23}$: +49.8 (*c* 0.46 in $CHCl_3$) for *R*,*R*-enantiomer; ^1H NMR (600 MHz, $CDCl_3$): δ 7.45 (m, 2H), 7.40 – 7.36 (m, 4H), 7.35 – 7.30 (m, 4H), 7.24 (d, *J* = 8.5 Hz, 1H), 6.95 (d, *J* = 8.6 Hz, 1H), 5.14 (s, 2H), 5.05 (d, *J* = 11.0 Hz, 1H), 5.00 (d, *J* = 11.0 Hz, 1H), 4.64 (br s, 1H), 4.52 (d, *J* = 7.1 Hz, 1H), 3.80 – 3.73 (m, 1H), 2.82 (ddd, *J* = 17.7, 5.5, 5.5 Hz, 1H), 2.73 (ddd, *J* = 17.9, 9.0, 6.0 Hz, 1H), 2.12 – 2.05 (m, 1H), 1.67 (dddd, *J* = 13.1, 9.4, 9.4, 5.9 Hz, 1H), 1.46 (s, 9H).; ^{13}C NMR (151 MHz, $CDCl_3$): δ 156.8, 151.0, 145.2, 137.9, 137.2, 131.2, 130.6, 128.7, 128.5, 128.1, 128.1, 127.6, 124.3, 113.1, 80.2, 74.4, 73.4, 71.0, 53.5, 28.5, 25.8, 22.1 (Carbonyl signal not visible); ESI-MS: *m/z* 498.0 $[M+Na]^+$.

tert-butyl-((1R,2R)-5,6-bis(benzyloxy)-1-((((R)-1-phenylethyl)carbamoyl)oxy)-1,2,3,4-tetrahydronaphthalen-2-yl)carbamate **(26)**

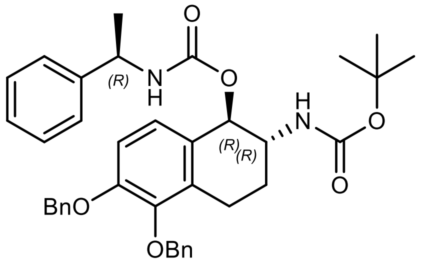

To a solution of compound **25** (7.00 g, 14.7 mmol) in absolute CH_2Cl_2 (150 mL) were added 2-3 drops of dibutyltin dilaurate and subsequently (*R*)-methylbenzyl isocyanate (2.49 mL, 17.7 mmol, *ee* >98%). The clear solution was stirred under nitrogen atmosphere at r.t. for 7 d. It was quenched with 2 M NaOH solution (50 mL, stirring for 30 min), the organic layer was separated and the aqueous layer was extracted again with CH_2Cl_2. The pooled, organic fractions were washed with water (2x), dried ($MgSO_4$) and evaporated, to give a beige powder in quantitative yield. The crude mixture of isomers was recrystallized from toluene/isohexane (1:1), allowing the hot and clear solution to cool down slowly over the course of several hours. After complete precipitation, the white powder was filtered under vacuum, washed with isohexane/toluene (4:1), followed by pure isohexane, yielding a residue consisting of 90% (*R,R,R*)-isomer (5.47 g). After a second recrystallization (toluene/isohexane 5:1, ~240 mL of solvent), analytically pure (*R,R,R*)-compound was obtained as a white powder (3.90 g, 85%, yield calcd. for single diastereomer).

mp: 174–176 °C; TLC: R_f = 0.42 (*iso*-hexane:acetone 2:1 v/v), diastereomer separation visible after 4x development of plate with toluene/ MTBE 7:1; $[\alpha]_D^{24}$: - 9.4 (*c* 0.85 in CH_2Cl_2); ^{1}H NMR (400 MHz, DMSO-*d*$_6$): δ 7.76 (d, *J* = 8.3 Hz, 1H), 7.48 (d, *J* = 6.8 Hz, 2H), 7.43 – 7.29 (m, 12H), 7.27 – 7.20 (m, 1H), 6.99 (d, *J* = 8.6 Hz, 1H), 6.85 (d, *J* = 8.2 Hz, 1H), 6.78 (d, *J* = 8.6 Hz, 1H), 5.60 (d, *J* = 6.9 Hz, 1H), 5.15 (s, 2H), 5.01 – 4.88 (m, 2H), 4.71 (m, 1H), 3.77 – 3.66 (m, 1H), 2.90 – 2.78 (m, 1H), 2.63 (ddd, *J* = 17.7, 6.5, 6.5 Hz, 1H), 1.95 – 1.85 (m, 1H), 1.80 – 1.67 (m, 1H), 1.39 (s, 9H), 1.33 (d, *J* = 7.1 Hz, 3H); ^{13}C NMR (151 MHz, DMSO-*d*$_6$): δ 155.6, 155.1, 150.5, 145.1, 144.4, 137.7, 137.1, 131.3, 128.4, 128.2, 128.2, 128.0, 127.8, 127.6, 126.5, 125.7, 124.2, 112.3, 77.7, 73.3, 71.7, 69.9, 50.2, 49.8, 28.2, 25.2, 22.9, 21.0; Chiral HPLC: System Ch1: t_R = 12.6 min, *de* >99.5%; ESI-MS: *m/z* 645.3 $[M+Na]^+$.

(1R,2R)-5,6-bis(benzyloxy)-2-(methylamino)-1,2,3,4-tetrahydronaphthalen-1-ol **(<u>27</u>)**

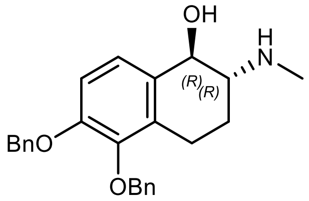

To a solution of compound **26** (60 mg, 0.096 mmol) in THF (2 mL) was added 4 M $LiAlH_4$ solution in Et_2O (145 µL, 0.58 mmol, 6 eq.) and the resulting reaction mixture was heated to 85 °C for 1 h. After careful addition of water and extraction with CH_2Cl_2 (3x), the combined organic layers were washed with brine, dried over $MgSO_4$ and evaporated. The resulting crude solid was purified by flash column chromatography (gradient, CH_2Cl_2 to CH_2Cl_2/MeOH 9:1) to yield a beige powder (23.1 mg, 62% yield).

<u>Alternative:</u> **27** can also be synthesized from (*R*,*R*)-**25** using 3 eq. of $LiAlH_4$-solution, obtaining yields of >80% of *N*-methylamine, after column chromatography.

TLC: R_f = 0.08 (CH_2Cl_2:MeOH 9:1 v/v + 0.1% 25% $NH_{3(aq)}$); $[\alpha]_D^{23}$**:** +14.1 (*c* 0.47 in $CHCl_3$); ^{1}H NMR (600 MHz, CD_3OD): δ 7.48 (m, 2H), 7.38 – 7.34 (m, 4H), 7.34 – 7.28 (m, 4H), 7.22 (d, *J* = 8.6 Hz, 1H), 7.03 (d, *J* = 8.6 Hz, 1H), 5.15 (s, 2H), 4.99 (d, *J* = 10.8 Hz, 1H), 4.97 (d, *J* = 10.8 Hz, 1H), 4.40 (d, *J* = 7.9 Hz, 1H), 2.86 (ddd, *J* = 17.6, 5.1, 5.1 Hz, 1H), 2.65 – 2.58 (m, 2H), 2.46 (s, 3H), 2.16 – 2.09 (m, 1H), 1.50 (dddd, *J* = 13.2, 10.4, 10.4, 5.6 Hz, 1H); ^{13}C NMR (101 MHz, CD_3OD): δ 152.0, 146.0, 139.1, 138.6, 133.0, 132.1, 129.7, 129.5, 129.3, 129.0, 129.0, 128.8, 124.7, 113.7, 75.2, 72.5, 71.8, 63.0, 33.3, 24.8, 23.1; Chiral HPLC: System Ch1: t_R = 29.3 min, *ee* > 99.5%; ESI-MS: *m/z* 390.2 $[M+H]^+$.

(5R,6R)-6-(methylamino)-5,6,7,8-tetrahydronaphthalene-1,2,5-triol trifluoroacetate **(*(R,R)*-c-Epi)**

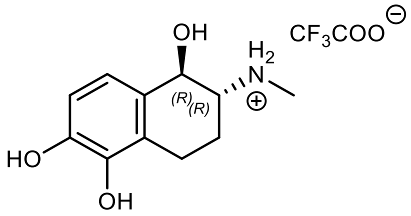

To a solution of **27** (230 mg, 0.59 mmol) in ethanol (15 mL) was added 10% Pd/C (23.0 mg) and the resulting suspension was stirred under hydrogen atmosphere for 2 h. The mixture was filtered through a syringe filter into 0.3% aqueous TFA (50 mL), and the formed solution was frozen and lyophilized. The crude TFA salt was purified by prep. HPLC (0.1% TFA in water + 3% acetonitrile to 10% acetonitrile in 10 min., 12 mL/min. flowrate, peak eluted at 5.0 min) to give ***(R,R)*-c-Epi** as a white powder (142 mg, 74% yield).

^{1}H NMR (600 MHz, CD_3OD): δ 6.89 (d, *J* = 8.3 Hz, 1H), 6.73 (d, *J* = 8.3 Hz, 1H), 4.63 (d, *J* = 8.9 Hz, 1H), 3.15 (ddd, *J* = 11.8, 8.8, 3.0 Hz, 1H), 3.00 (ddd, *J* = 17.7, 5.9, 3.4 Hz, 1H), 2.79 (s, 3H), 2.73 (ddd, *J* = 17.5, 11.0, 6.1 Hz, 1H), 2.36 (dddd, *J* = 12.8, 6.3, 3.2, 3.2 Hz, 1H), 1.82 (dddd, *J* = 12.0, 12.0, 12.0, 5.8 Hz, 1H); ^{13}C NMR (151 MHz, CD_3OD): δ 145.2, 143.2, 129.6, 123.5, 118.9, 114.6, 70.5, 63.3, 31.1, 23.2, 22.8; ESI-HRMS (*m*/*z*): $[MH]^+$ calcd. for $C_{11}H_{16}NO_3$, 210.1125; found, 210.1124; HPLC: System 1: t_R = 4.1 min, purity 99%, System 2: t_R = 4.8 min, purity 98%.

(1R,2R)-2-amino-5,6-bis(benzyloxy)-1,2,3,4-tetrahydronaphthalen-1-ol (**28**)

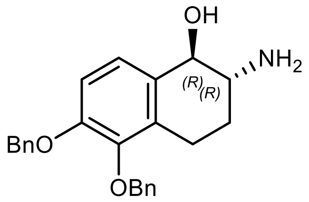

To a solution of **26** (1.08 g, 1.74 mmol) in THF (20 mL) was added 1 M TBAF solution (8.00 mL, 8.00 mmol). The mixture was heated to 90 °C for 4 h. Another portion of 1 M TBAF solution was added (6.00 mL, 6.00 mmol) and heating was continued for 6 h. The reaction was diluted with water (200 mL) and extracted with CH_2Cl_2 (3x). The organic layers were washed with water (3x), dried ($MgSO_4$) and evaporated. The resulting crude solid was purified by flash column chromatography (gradient, CH_2Cl_2 to CH_2Cl_2/MeOH 9:1) to yield **28** as a beige powder (592 mg, 91% yield). The absolute configuration was determined by derivatization with Mosher's reagent (*N*-acylation) and subsequent ^{1}H NMR studies[10].

Alternatively, **28** can be synthesized from (*R*,*R*)-**25** using 3-5 eq. of TBAF solution in almost quantitative yield.

TLC: R_f = 0.05 (CH_2Cl_2:MeOH 9:1 v/v + 0.1% 25% $NH_{3(aq)}$); ^{1}H NMR (400 MHz, CD_3OD): δ 7.51 – 7.45 (m, 2H), 7.41 – 7.28 (m, 8H), 7.26 (dd, *J* = 8.6, 1.0 Hz, 1H), 7.07 (d, *J* = 8.6 Hz, 1H), 5.16 (s, 2H), 5.01 (d, *J* = 10.8 Hz, 1H), 4.97 (d, *J* = 10.8 Hz, 1H), 4.47 (d, *J* = 8.9 Hz, 1H), 3.06 (ddd, *J* = 12.0, 8.9, 3.3 Hz, 1H), 2.95 (ddd, *J* = 17.8, 5.7, 3.1 Hz, 1H), 2.67 (ddd, *J* = 17.6, 11.3, 6.0 Hz, 1H), 2.12 (dddd, *J* = 12.7, 6.2, 3.2, 3.2 Hz, 1H), 1.73 (dddd, *J* = 12.9, 11.5, 11.5, 5.8 Hz, 1H); ESI-MS: *m/z* 376.2 $[M+H]^+$.

(5R,6R)-6-amino-5,6,7,8-tetrahydronaphthalene-1,2,5-triol trifluoroacetate **(*(R,R)*-c-NorEpi)**

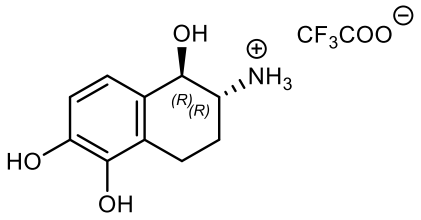

To a solution of **28** (50.0 mg, 0.13 mmol) in ethanol (3 mL) was added 10% Pd/C (5.00 mg) and the resulting suspension was stirred under hydrogen atmosphere for 1 h. The mixture was filtered through a syringe filter into 0.3% aqueous TFA (30 mL), and the formed solution was frozen and lyophilized. The crude TFA salt was purified by prep. HPLC (0.1% TFA in water + 2% acetonitrile, 12 mL/min. flowrate, peak eluted at 3.8 min) to give ***(R,R)*-c-NorEpi** as a beige powder (13.8 mg, 33% yield).

^{1}H NMR (400 MHz, CD_3OD): δ 6.89 (d, *J* = 8.3 Hz, 1H), 6.73 (d, *J* = 8.3 Hz, 1H), 4.55 (d, *J* = 8.9 Hz, 1H), 3.20 (ddd, *J* = 12.0, 8.9, 3.0 Hz, 1H), 2.97 (ddd, *J* = 17.8, 6.0, 2.9 Hz, 1H), 2.72 (ddd, *J* = 17.6, 11.3, 6.0 Hz, 1H), 2.24 (dddd, *J* = 12.6, 6.2, 3.1, 3.1 Hz, 1H), 1.86 (dddd, *J* = 12.8, 11.5, 11.5, 6.1 Hz, 1H); ^{13}C NMR (151 MHz, CD_3OD): δ 145.2, 143.2, 129.8, 123.6, 118.8, 114.5, 71.6, 55.7, 26.0, 22.8; ESI-HRMS (*m/z*): $[MH]^+$ calcd. for $C_{10}H_{14}NO_3$, 196.0968; found, 196.0967; HPLC: System 1: t_R = 4.0 min, purity 98%, System 2: t_R = 4.1 min, purity 95%.

(5R,6R)-6-(isopropylamino)-5,6,7,8-tetrahydronaphthalene-1,2,5-triol trifluoroacetate **(8, *(R,R)*-c-ISO))**

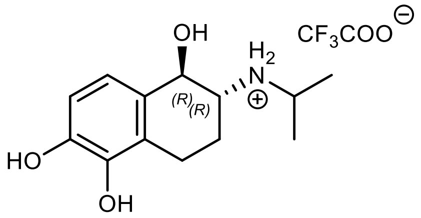

Alternative procedure: To a solution of **28** (40.0 mg, 0.106 mmol) in CH_2Cl_2 (2 mL) was added acetone (100 µL, 1.34 mmol) and sodium triacetoxyborohydride (67.0 mg, 0.32 mmol). The suspension was stirred for 1 h at room temperature. After addition of sat. $NaHCO_3$ solution and extraction with CH_2Cl_2 (3x), the combined, organic layers were washed with brine, dried ($MgSO_4$) and evaporated. The crude compound was purified by flash column chromatography (CH_2Cl_2 to CH_2Cl_2/ methanol 100:1 to 25:1) to give a white powder (35.1 mg, 79% yield). To a solution of this intermediate (26.0 mg, 0.062 mmol) in ethanol (3 mL) was added Pd/C 10% (3.0 mg) and the resulting suspension was stirred under hydrogen atmosphere for 2 h. After filtration and addition of 0.1% TFA, the solution was frozen and lyophilized. The residue was purified by preparative HPLC (10 mL/min, 0.1% TFA + 5% acetonitrile to 30% at 6 min, peak eluted at 4.5 min) to give **(*R*,*R*)-c-ISO** (**8**) as a white powder (10.2 mg, 48% yield).

Supplementary References:

1 Xu, X. *et al.* Binding pathway determines norepinephrine selectivity for the human beta1AR over beta2AR *Cell Res* **31**, 569-579, doi:10.1038/s41422-020-00424-2 (2021).

2 van der Westhuizen, E. T., Breton, B., Christopoulos, A. & Bouvier, M. Quantification of ligand bias for clinically relevant beta2-adrenergic receptor ligands: implications for drug taxonomy. *Mol Pharmacol* **85**, 492-509, doi:10.1124/mol.113.088880 (2014).

3 Zhang, F., Yao, D., Hua, Y., van Breemen, R. B. & Bolton, J. L. Synthesis and Reactivity of the Catechol Metabolites from the Equine Estrogen, 8,9-Dehydroestrone. *Chemical Research in Toxicology* **14**, 754-763, doi:10.1021/tx010049y (2001).

4 Macchia, B. *et al.* Conformational Effects on the Activity of Drugs. 13. A Revision of Previously Proposed Models for the Activation of α- and β-Adrenergic Receptors *J. Med. Chem.* **35**, 1009-1018 (1992).

5 Bonner, L. A. *et al.* Mapping the Catechol Binding Site in Dopamine D1 Receptors: Synthesis and Evaluation of Two Parallel Series of Bicyclic Dopamine Analogues. *ChemMedChem* **6**, 1024-1040, doi:https://doi.org/10.1002/cmdc.201100010 (2011).

6 Itoh, K. *et al.* The Syntheses and &beta-Adrenoceptor Activities of N-Substituted 2-Amino-5, 6-dihydroxy-1, 2, 3, 4-tetrahydro-1-naphthalenols. *CHEMICAL & PHARMACEUTICAL BULLETIN* **25**, 2917-2928, doi:10.1248/cpb.25.2917 (1977).

7 Oka, Y. *et al.* Syntheses of conformationally Rigid Catecholamine Derivatives. *CHEMICAL & PHARMACEUTICAL BULLETIN* **25**, 632-639, doi:10.1248/cpb.25.632 (1977).

8 Labafzadeh, S. R., Kavakka, J. S., Vyavaharkar, K., Sievänen, K. & Kilpeläinen, I. Preparation of cellulose and pulp carbamates through a reactive dissolution approach. *RSC Advances* **4**, 22434-22441, doi:10.1039/C4RA02316A (2014).

9 Routier, S., Saugé, L., Ayerbe, N., Coudert, G. & Mérour, J.-Y. A mild and selective method for N-Boc deprotection. *Tetrahedron Letters* **43**, 589-591, doi:https://doi.org/10.1016/S0040-4039(01)02225-0 (2002).

10 Hoye, T. R., Jeffrey, C. S. & Shao, F. Mosher ester analysis for the determination of absolute configuration of stereogenic (chiral) carbinol carbons. *Nature Protocols* **2**, 2451-2458, doi:10.1038/nprot.2007.354 (2007).
