# Supplementary material for: Constrained catecholamines gain β2AR selectivity through allosteric effects on pocket dynamics
Source: Nat Commun. 2023 Apr 14;14:2138. doi: 10.1038/s41467-023-37808-y (PMC10104803; doi:10.1038/s41467-023-37808-y)
Supplement: Supplementary file 3 — Reporting Summary [file 41467_2023_37808_MOESM3_ESM.pdf]

nature research

Corresponding author(s): Brian K. Kobilka, Roger K. Sunahara, Xiangyu Liu, Peter Gmeiner

Last updated by author(s): May 25, 2022

Reporting Summary

Nature Research wishes to improve the reproducibility of the work that we publish. This form provides structure for consistency and transparency in reporting. For further information on Nature Research policies, see our Editorial Policies and the Editorial Policy Checklist.

Statistics

For all statistical analyses, confirm that the following items are present in the figure legend, table legend, main text, or Methods section.

n/a	Confirmed	
☐	☒	The exact sample size (*n*) for each experimental group/condition, given as a discrete number and unit of measurement
☐	☒	A statement on whether measurements were taken from distinct samples or whether the same sample was measured repeatedly
☐	☒	The statistical test(s) used AND whether they are one- or two-sided<br>*Only common tests should be described solely by name; describe more complex techniques in the Methods section.*
☒	☐	A description of all covariates tested
☒	☐	A description of any assumptions or corrections, such as tests of normality and adjustment for multiple comparisons
☐	☒	A full description of the statistical parameters including central tendency (e.g. means) or other basic estimates (e.g. regression coefficient) AND variation (e.g. standard deviation) or associated estimates of uncertainty (e.g. confidence intervals)
☐	☒	For null hypothesis testing, the test statistic (e.g. *F*, *t*, *r*) with confidence intervals, effect sizes, degrees of freedom and *P* value noted<br>*Give P values as exact values whenever suitable.*
☒	☐	For Bayesian analysis, information on the choice of priors and Markov chain Monte Carlo settings
☒	☐	For hierarchical and complex designs, identification of the appropriate level for tests and full reporting of outcomes
☒	☐	Estimates of effect sizes (e.g. Cohen's *d*, Pearson's *r*), indicating how they were calculated

Our web collection on statistics for biologists contains articles on many of the points above.

Software and code

Policy information about availability of computer code

Data collection	AMBER 16,18
Data analysis	phenix-1.12-2829, Coot 0.8.9.1, CCP4 7.0.004, Pymol version 2.1.1, matplotlib 3.0.2, GraphPad Prism 6.0 and 8.0, Molprobity 4.0

For manuscripts utilizing custom algorithms or software that are central to the research but not yet described in published literature, software must be made available to editors and reviewers. We strongly encourage code deposition in a community repository (e.g. GitHub). See the Nature Research guidelines for submitting code & software for further information.

Data

Policy information about availability of data

All manuscripts must include a data availability statement. This statement should provide the following information, where applicable:

- Accession codes, unique identifiers, or web links for publicly available datasets
- A list of figures that have associated raw data
- A description of any restrictions on data availability

Atomic coordinates and structure factors have been deposited in the Protein Data Bank (PDB) under accession codes 7XKA and 7XK9. All the other relevant structures referenced in this work are available under the accession codes 3SN6,4LDO, 6K41,7BU7

Field-specific reporting

Please select the one below that is the best fit for your research. If you are not sure, read the appropriate sections before making your selection.

☒ Life sciences ☐ Behavioural & social sciences ☐ Ecological, evolutionary & environmental sciences

For a reference copy of the document with all sections, see nature.com/documents/nr-reporting-summary-flat.pdf

Life sciences study design

All studies must disclose on these points even when the disclosure is negative.

Sample size	A sample size of n=3 is commonly used in biological studies. All experiments were performed in at least three independent biologial replicates.
Data exclusions	No data were excluded.
Replication	All experiments were repeated at least three times to ensure reproducibility.
Randomization	Cells placed in different positions on 96-well signaling assay plate and randomly allocated into control and treatment groups.
Blinding	Blinding is not relevant to this study, all experiments were performed based on standardized protocols and readouts and are not influenced by the investigator.

Reporting for specific materials, systems and methods

We require information from authors about some types of materials, experimental systems and methods used in many studies. Here, indicate whether each material, system or method listed is relevant to your study. If you are not sure if a list item applies to your research, read the appropriate section before selecting a response.

Materials & experimental systems

n/a	Involved in the study	
☒	☐	Antibodies
☐	☒	Eukaryotic cell lines
☒	☐	Palaeontology and archaeology
☒	☐	Animals and other organisms
☒	☐	Human research participants
☒	☐	Clinical data
☒	☐	Dual use research of concern

Methods

n/a	Involved in the study	
☒	☐	ChIP-seq
☒	☐	Flow cytometry
☒	☐	MRI-based neuroimaging

Eukaryotic cell lines

Policy information about cell lines

Cell line source(s)	Sf9 cells were purchased from Expression Systems, Cat 94-0015, .HEK293T cells (ATCC CRL-3216) , The deltaGNAS cell line was
Authentication	generously provided by Dr. Asuka Inoue.
Mycoplasma contamination	HEK293T and HEK 293 have been validated by the supplier through STR analysis and cytogenetic studies. Cell lines are
Commonly misidentified lines (See ICLAC register)	tested by manufacturer for contamination and no additional testing was conducted by authors of this study. no commonly

misidentified cell lines were used.
